# Supplementary material for: Neuroprotective and Antiherpetic Properties of Polyphenolic Compounds from Maackia amurensis Heartwood
Source: Molecules. 2023 Mar 13;28(6):2593. doi: 10.3390/molecules28062593 (PMC10056899; doi:10.3390/molecules28062593)
Supplement: Supplementary file 1 [file molecules-28-02593-s001.zip › molecules-2250603-supplementary.pdf]

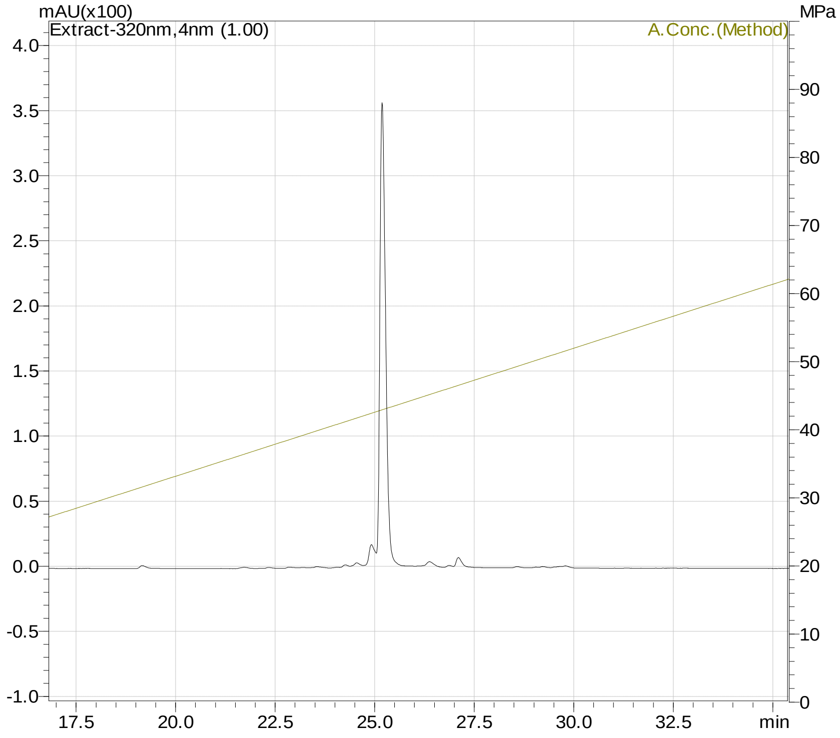


Figure S1. HPLC profile of compounds **1a and 1b**.

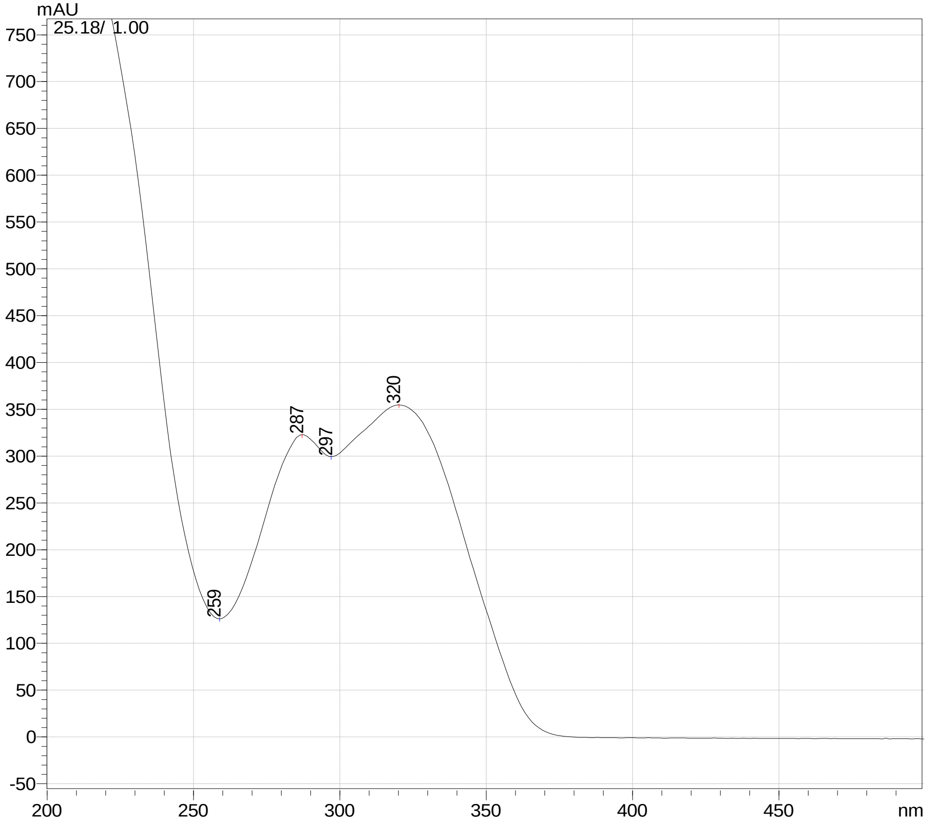


Figure S2. UV spectrum of compounds **1a and 1b**.

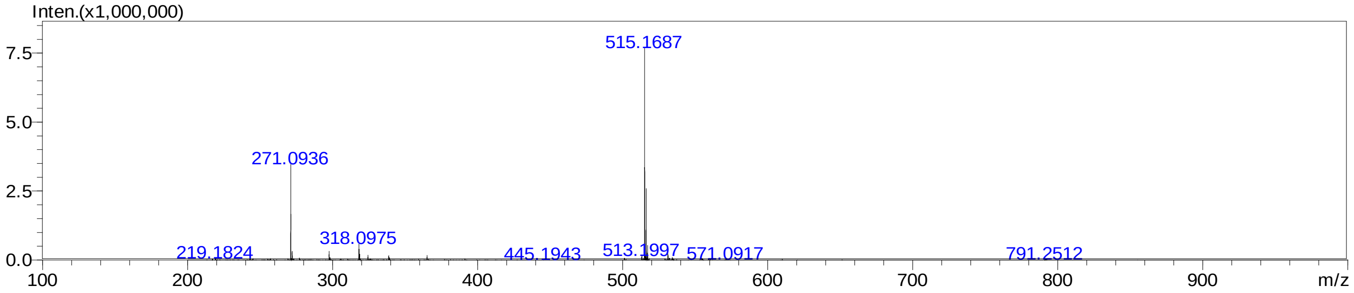


Figure S3. HR-MS spectrum of compounds **1a and 1b** (positive ion mode).

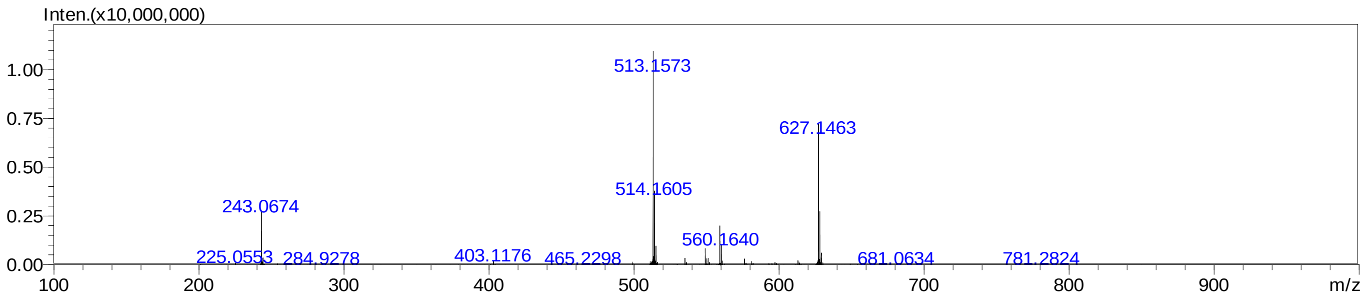


Figure S4. HR-MS spectrum of compounds **1a and 1b** (negative ion mode).

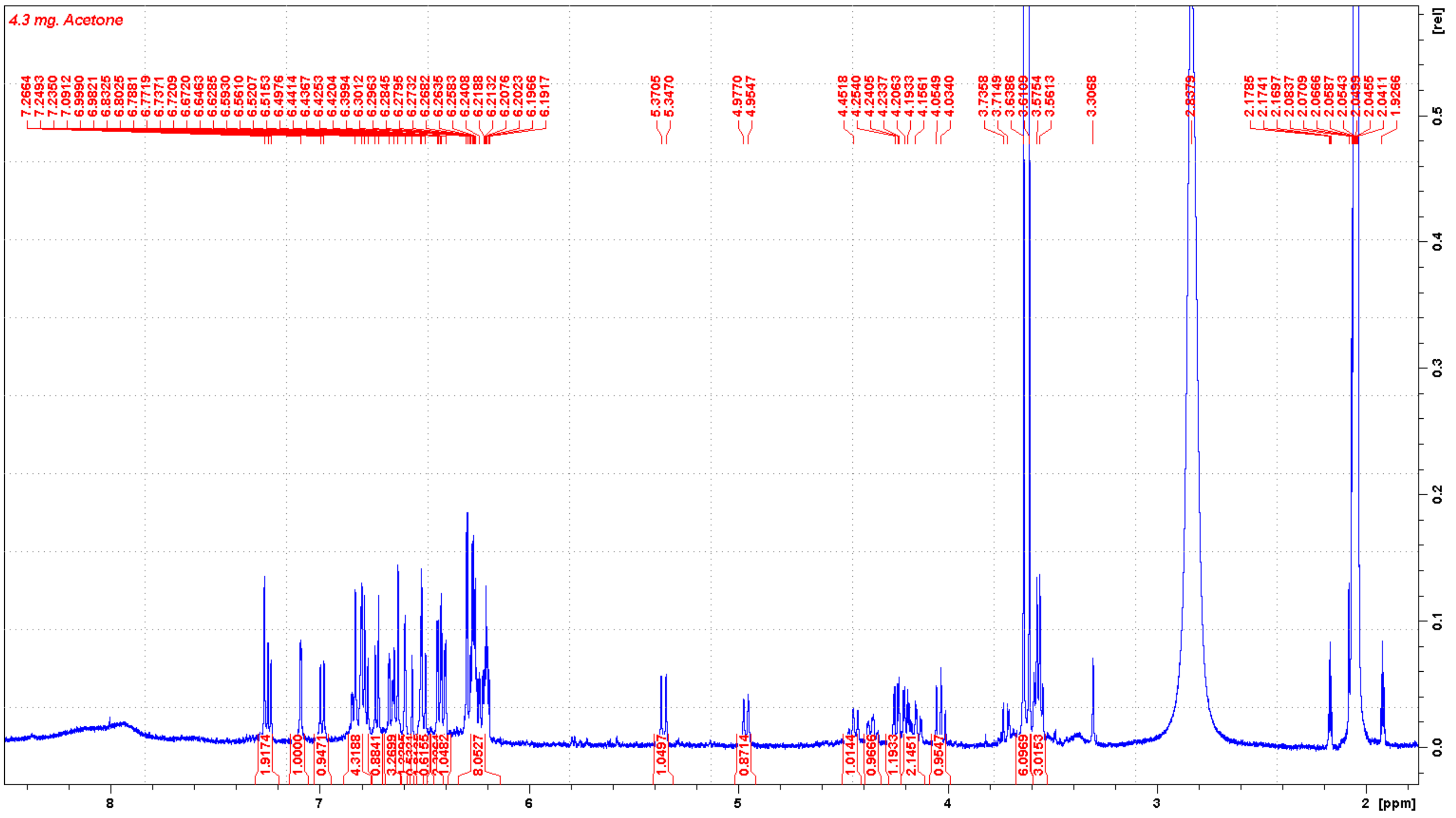


Figure S5. $^{1}$H NMR spectrum of compounds **1a and 1b**.

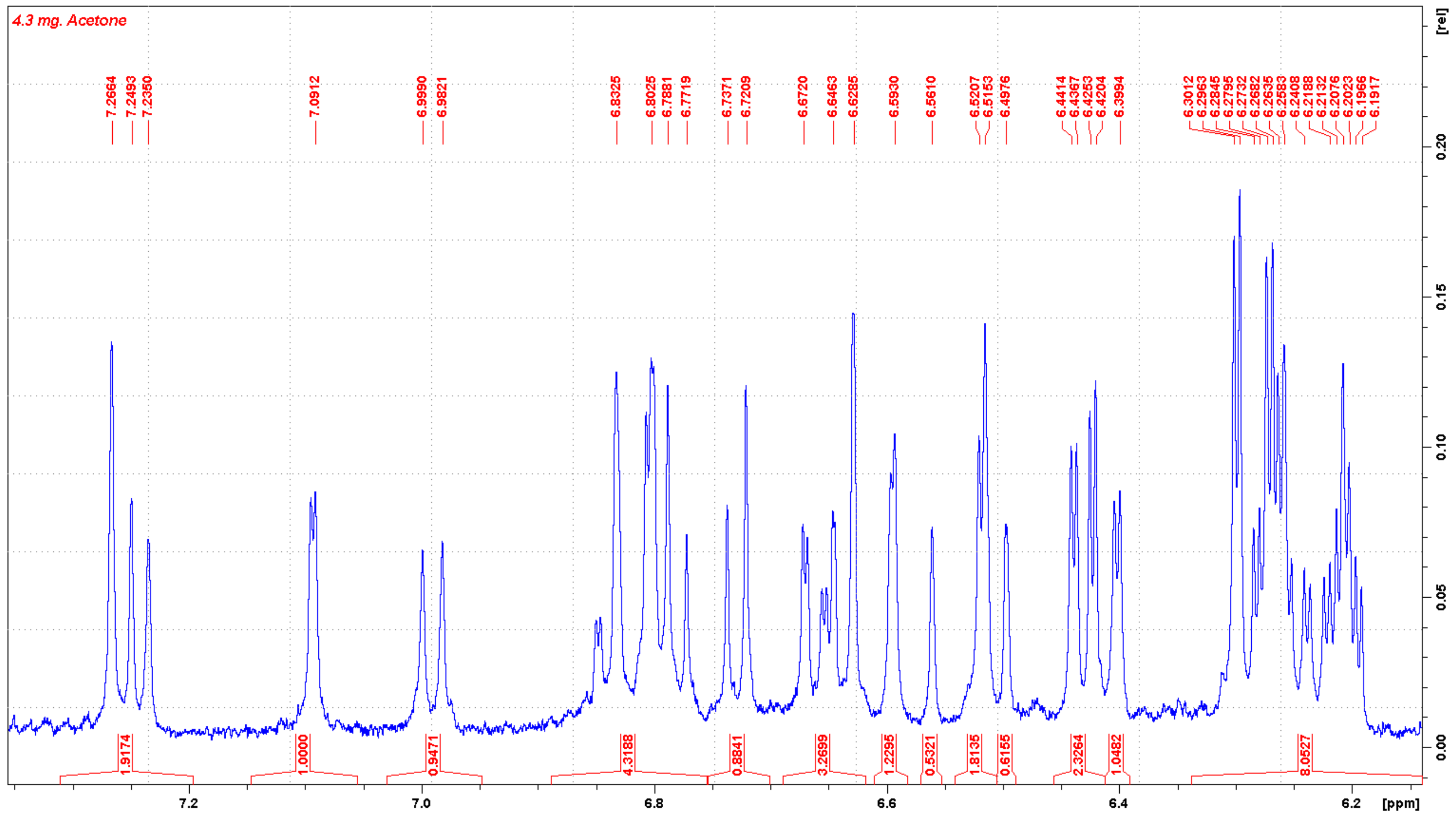


Figure S6. $^{1}$H NMR spectrum of compounds **1a and 1b** (enlarged).

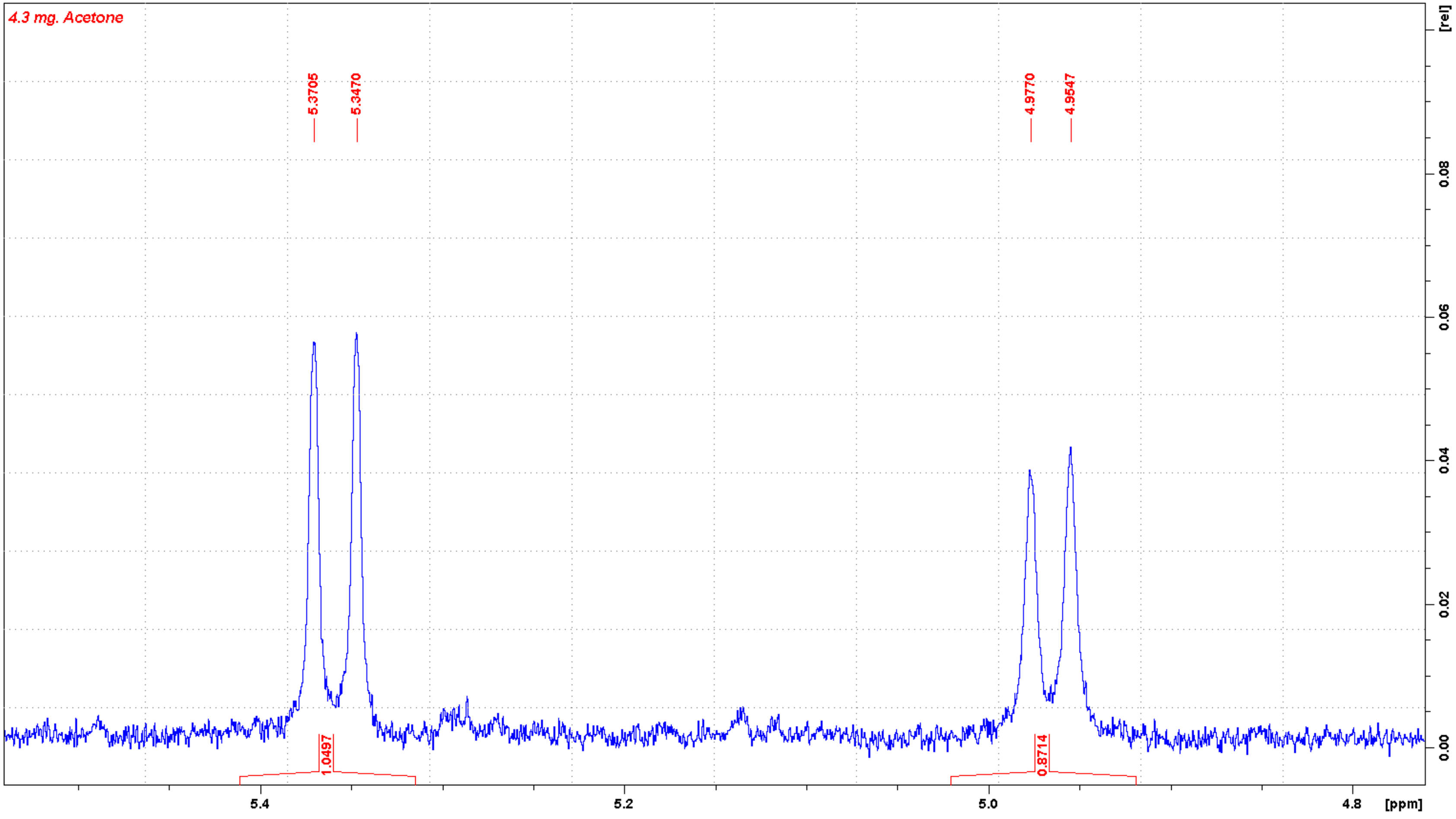


Figure S7. $^{1}$H NMR spectrum of compounds **1a and 1b** (enlarged).

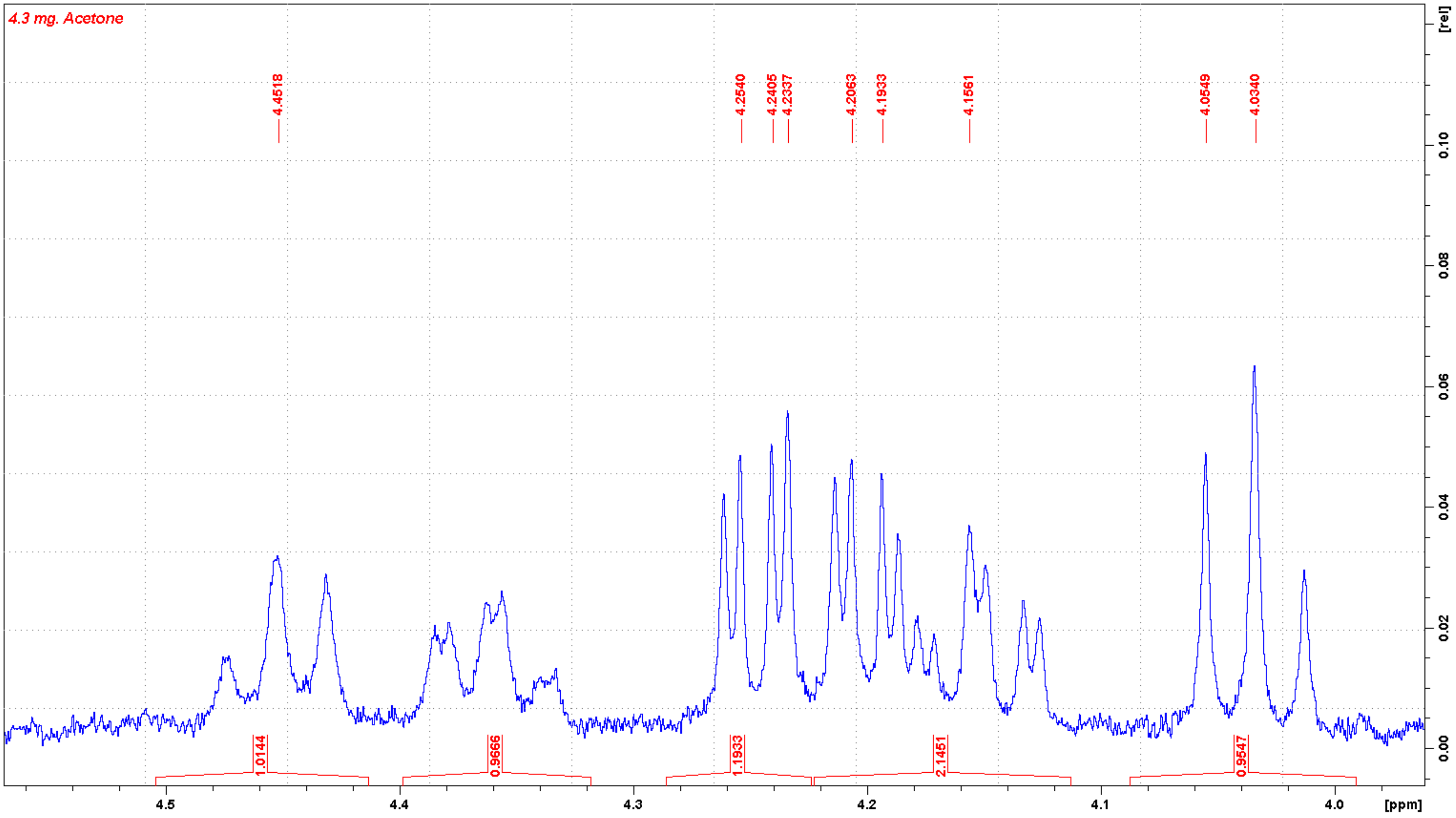


Figure S8. $^{1}$H NMR spectrum of compounds **1a and 1b** (enlarged).

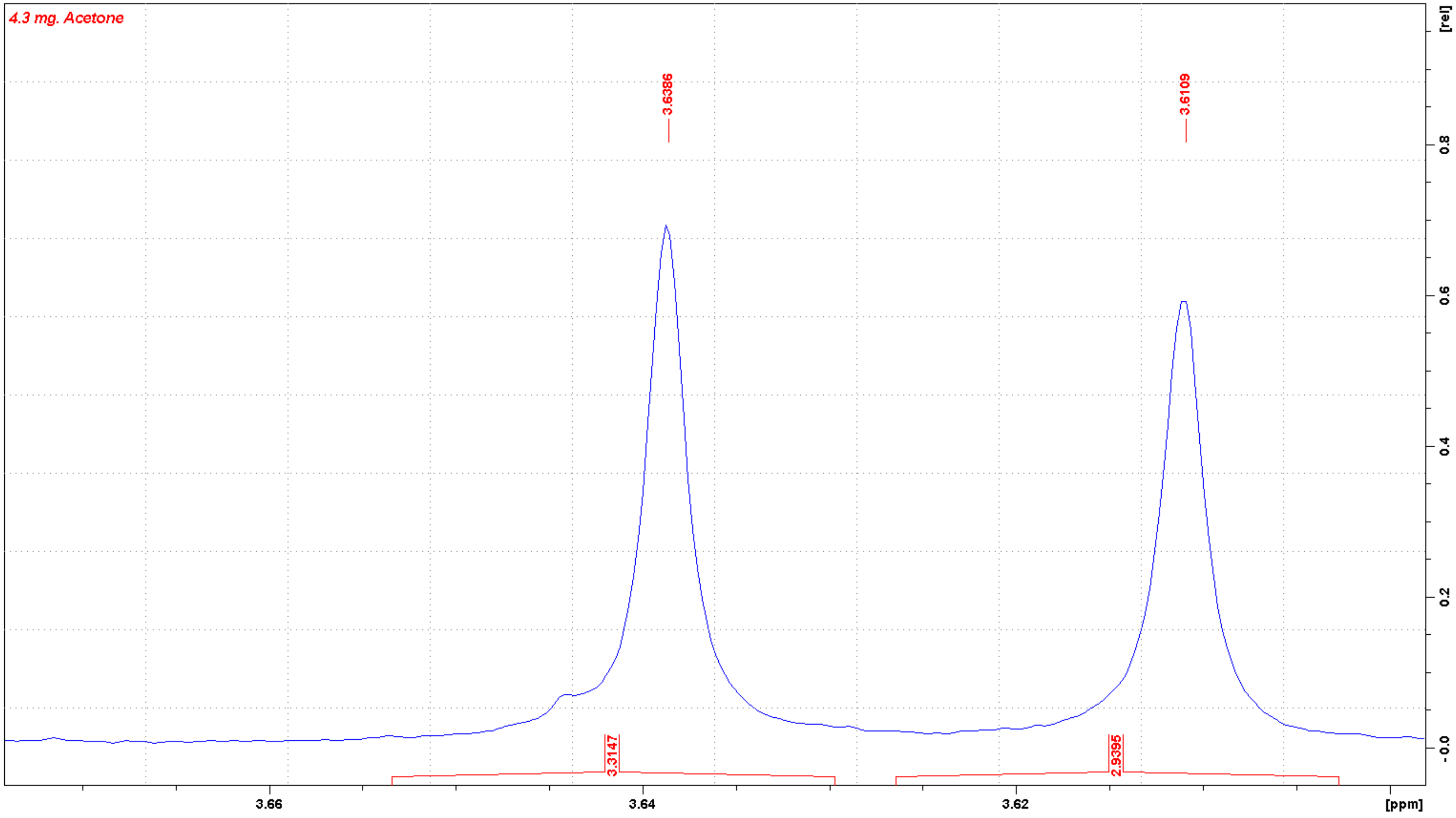


Figure S9. $^{1}H$ NMR spectrum of compounds **1a and 1b** (enlarged).

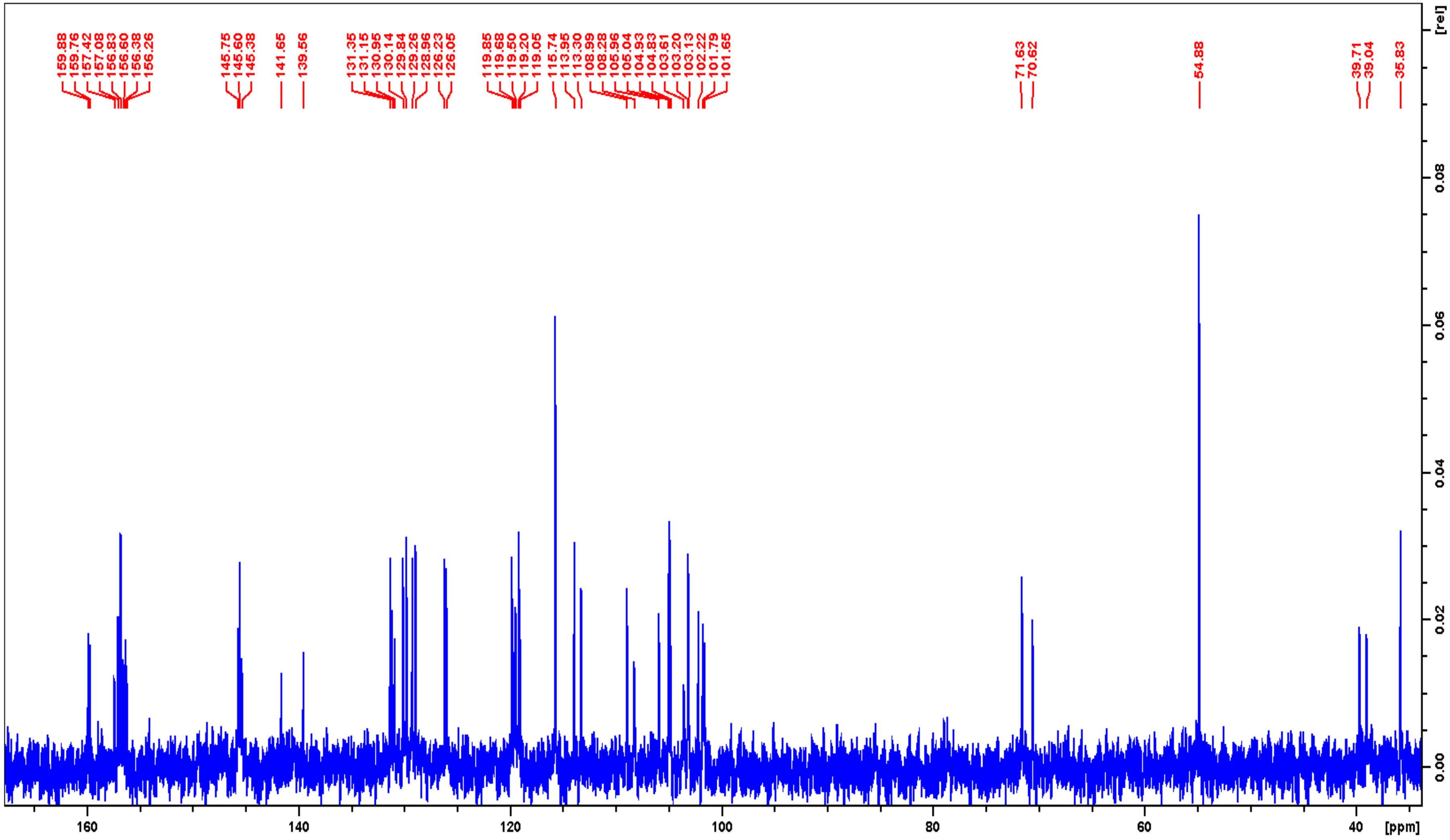


Figure S10. $^{13}C$ NMR spectrum of compounds **1a and 1b**.

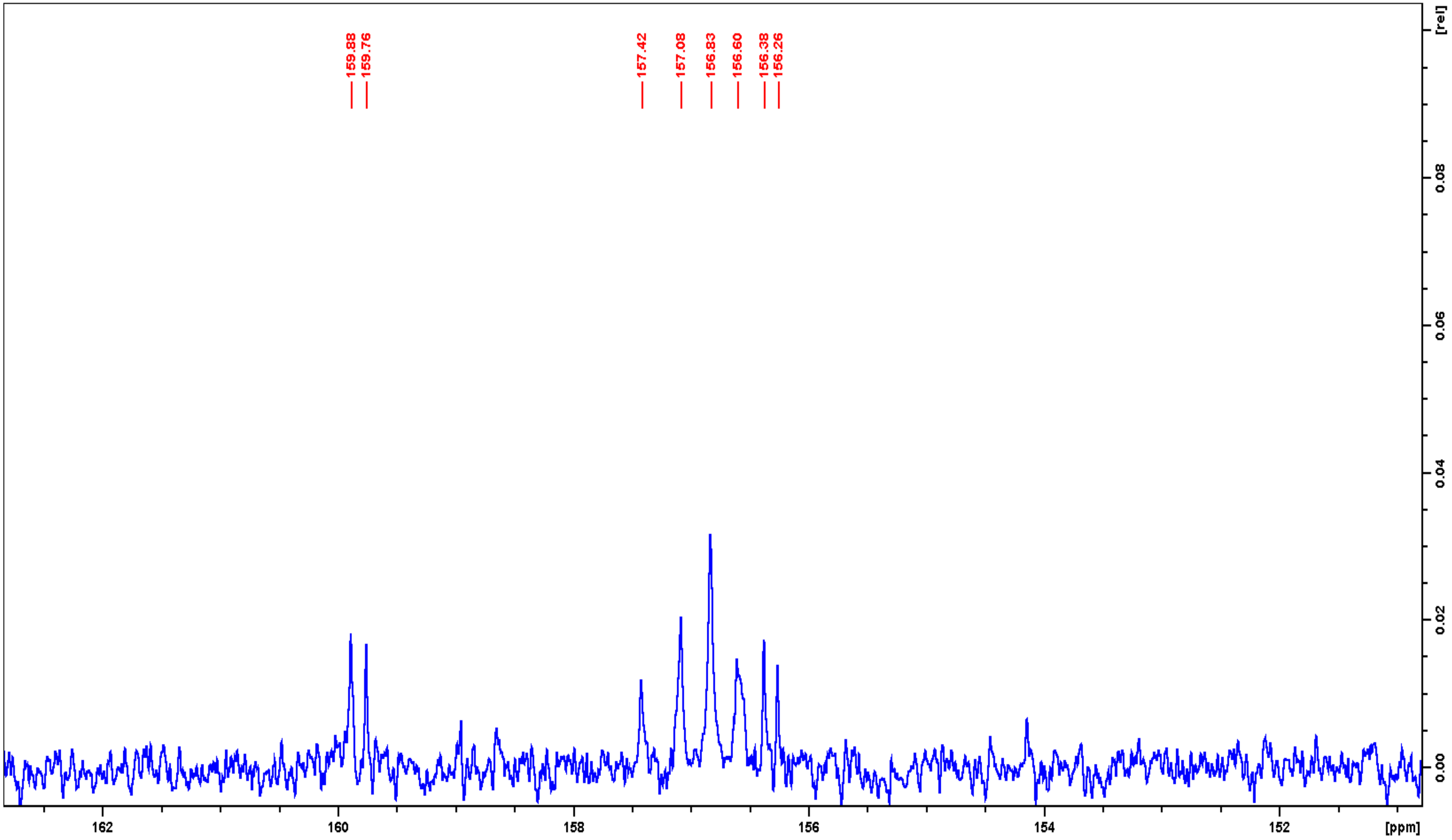


Figure S11. $^{13}$C NMR spectrum of compounds **1a and 1b** (enlarged).

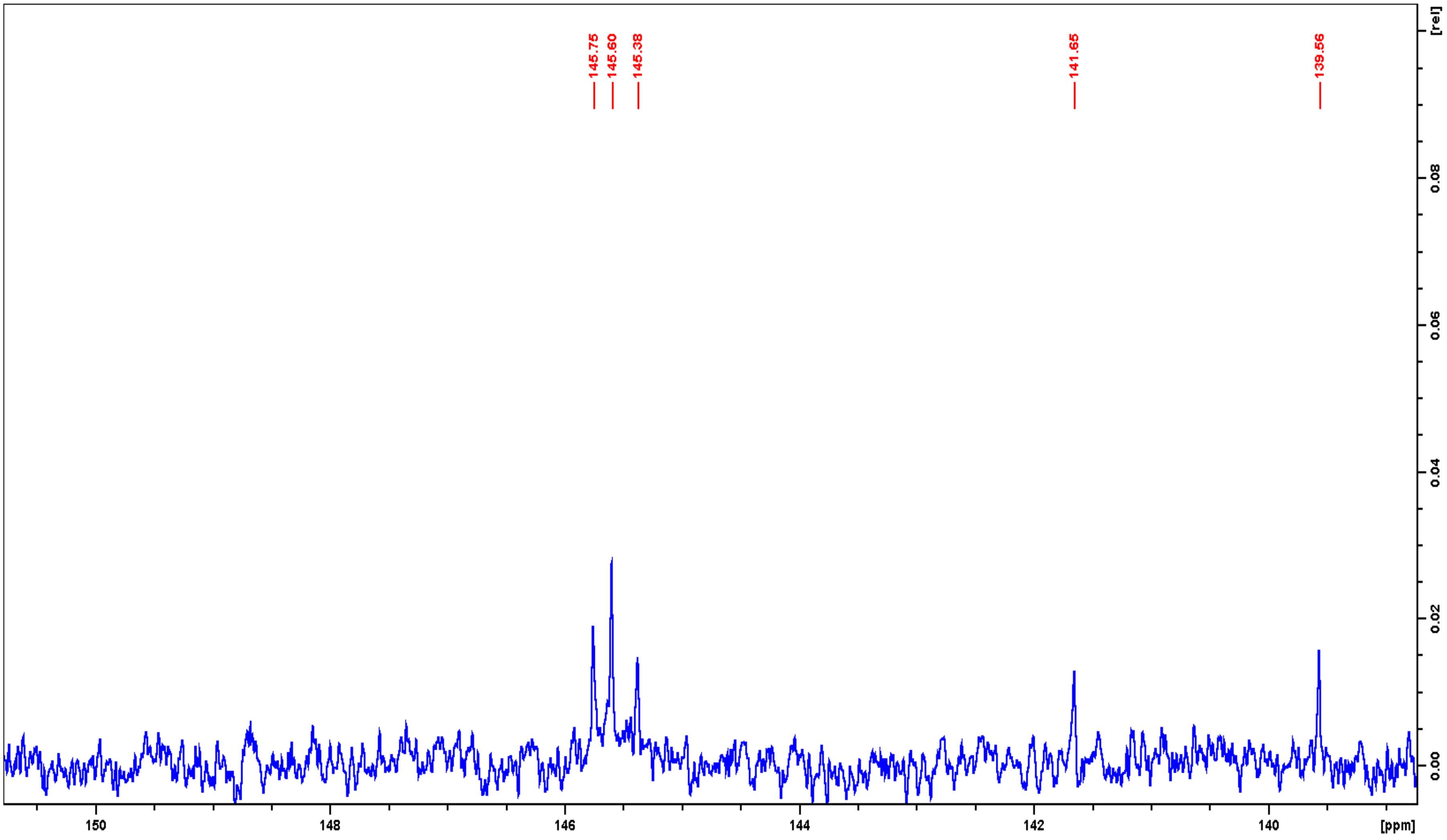


Figure S12. $^{13}$C NMR spectrum of compounds **1a and 1b** (enlarged).

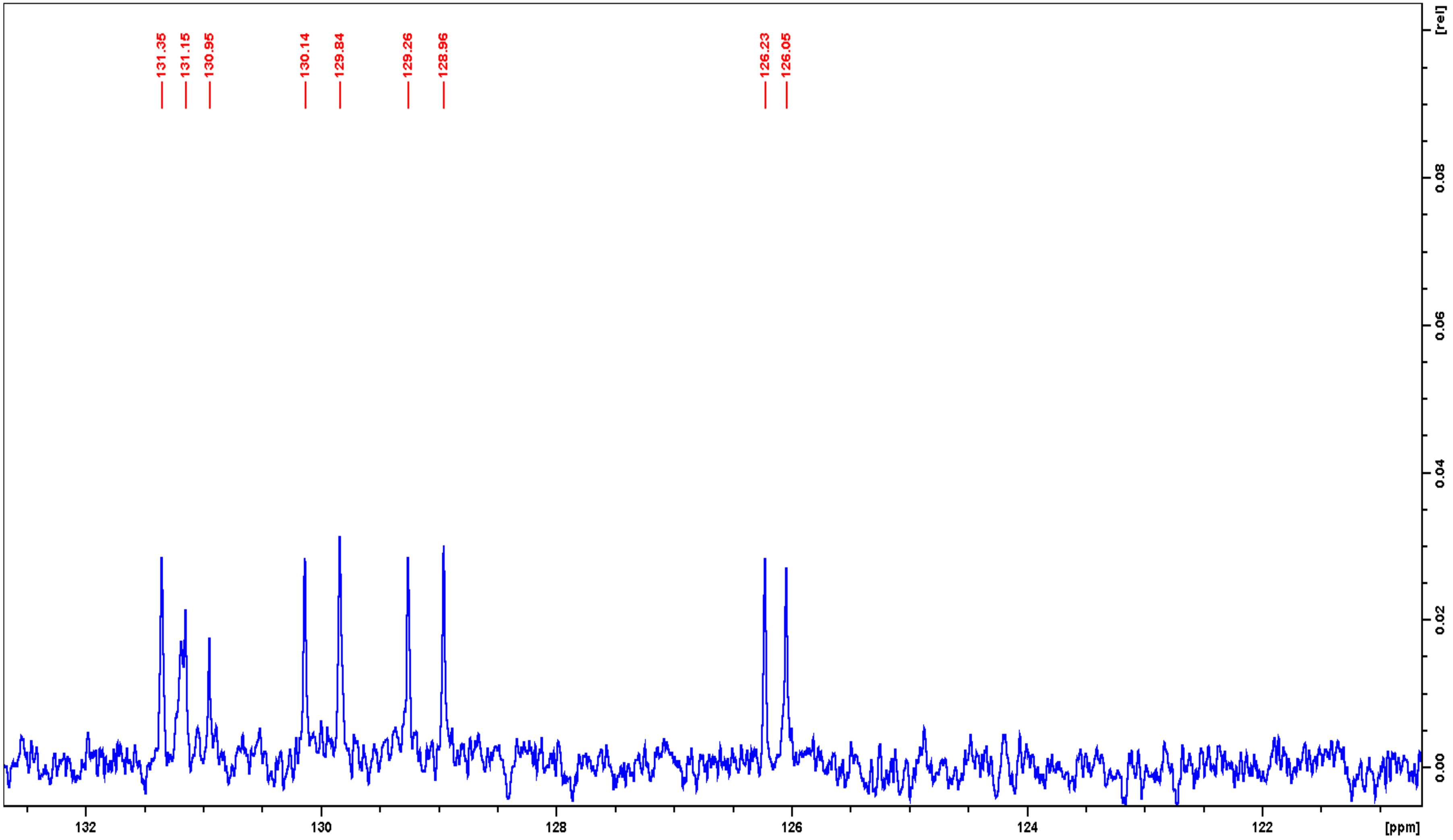


Figure S13. $^{13}C$ NMR spectrum of compounds **1a and 1b** (enlarged).

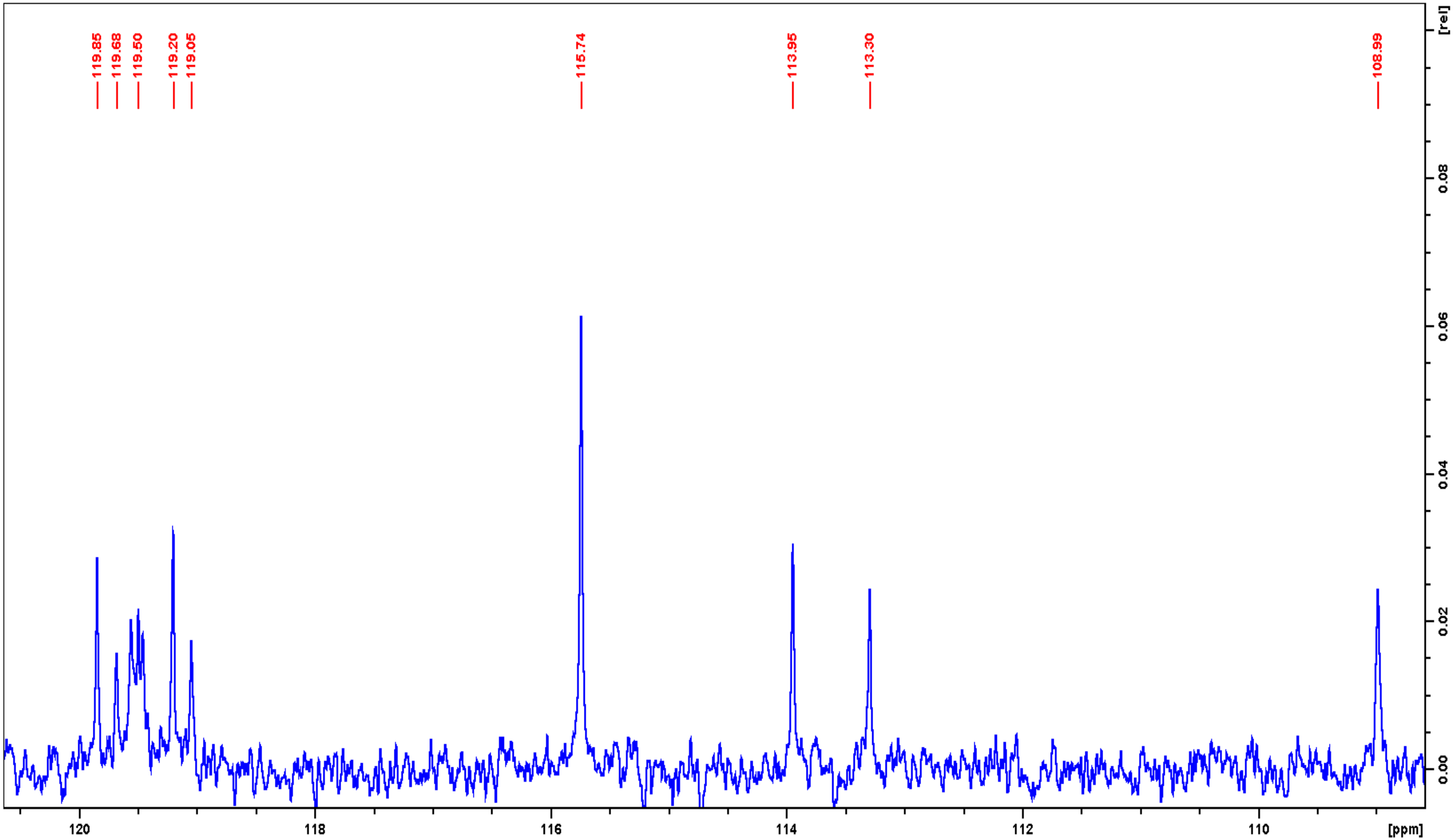


Figure S14. $^{13}$C NMR spectrum of compounds **1a and 1b** (enlarged).

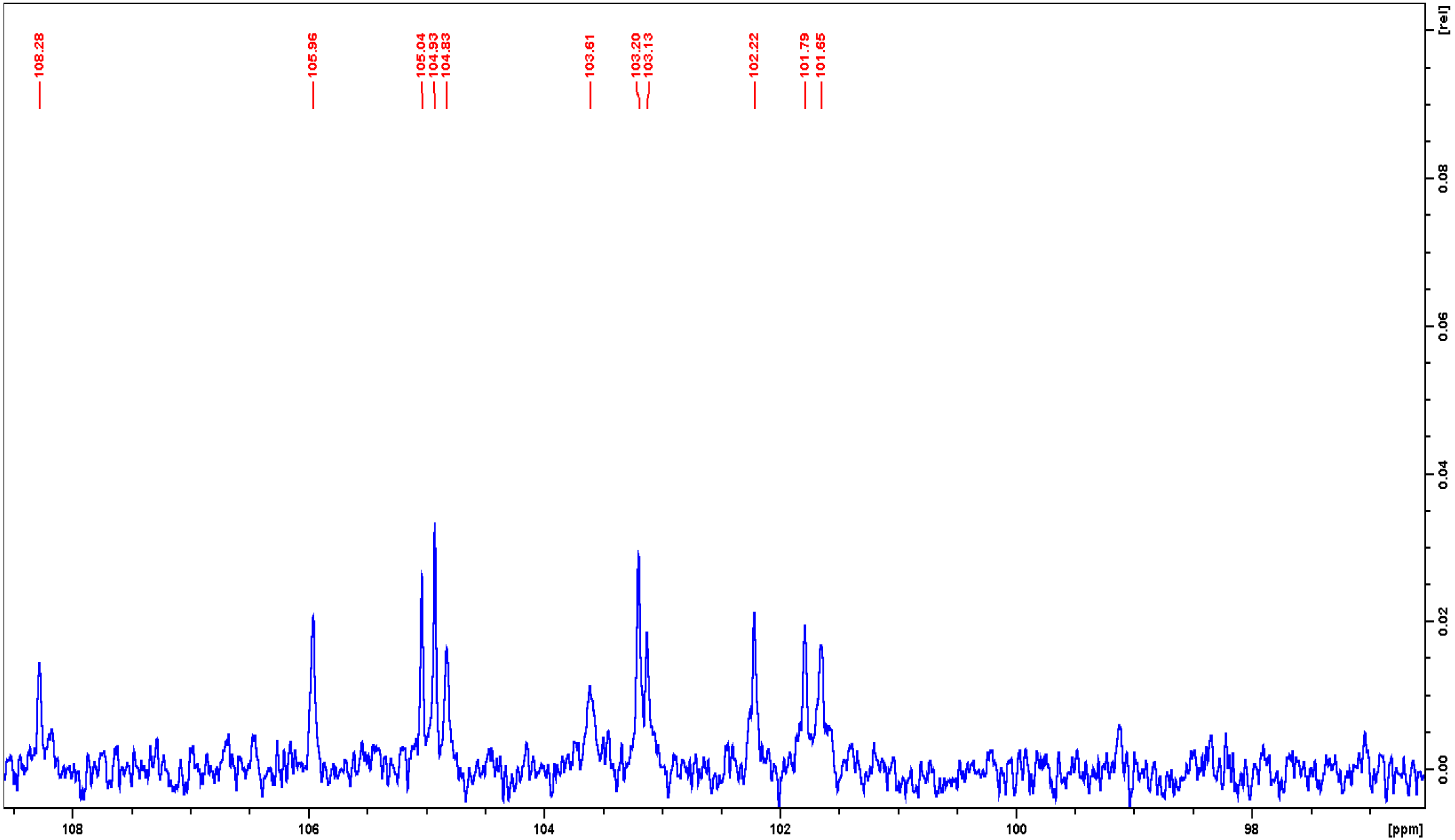


Figure S15. $^{13}$C NMR spectrum of compounds **1a and 1b** (enlarged).

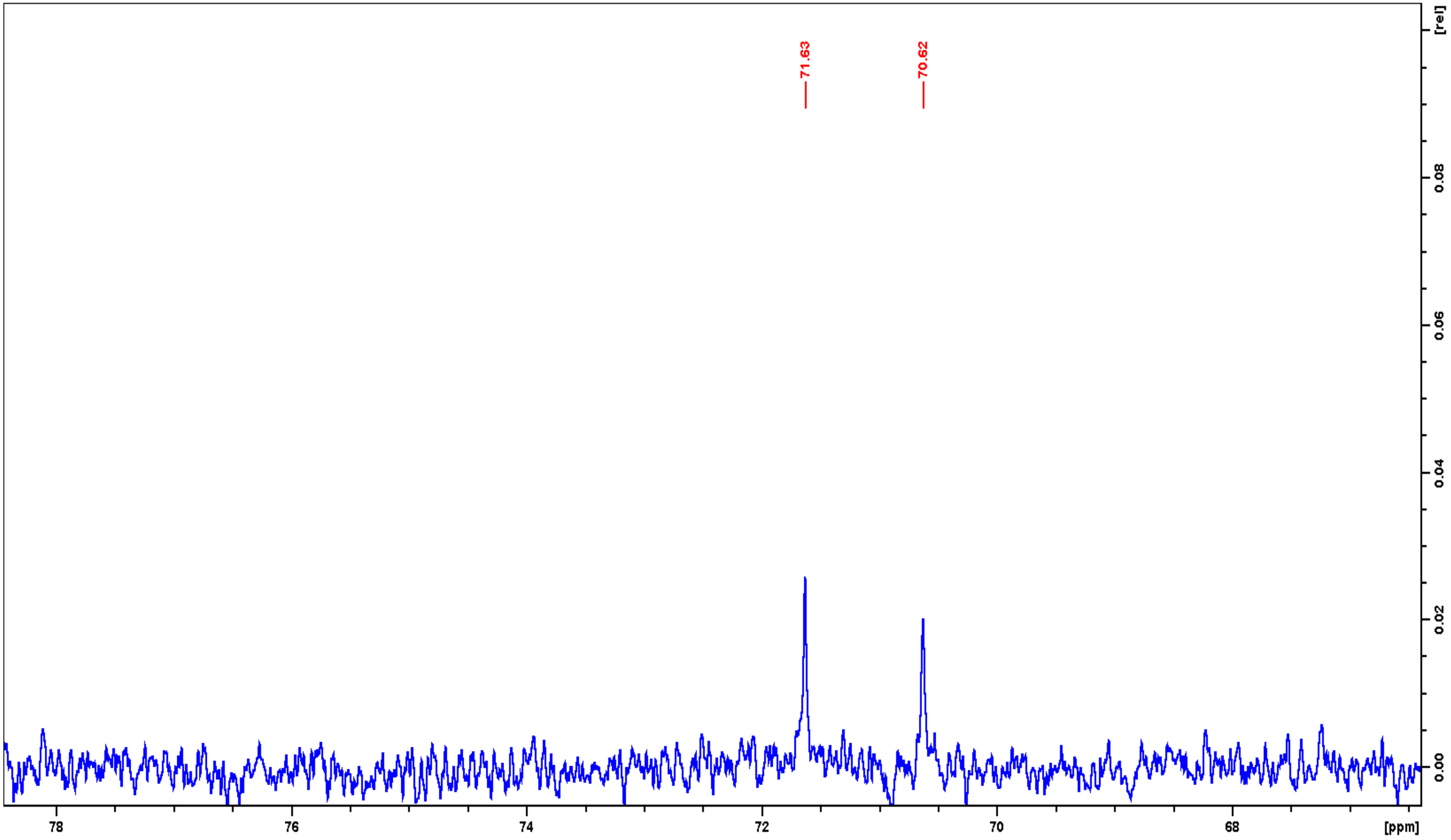


Figure S16. $^{13}$C NMR spectrum of compounds **1a and 1b** (enlarged).

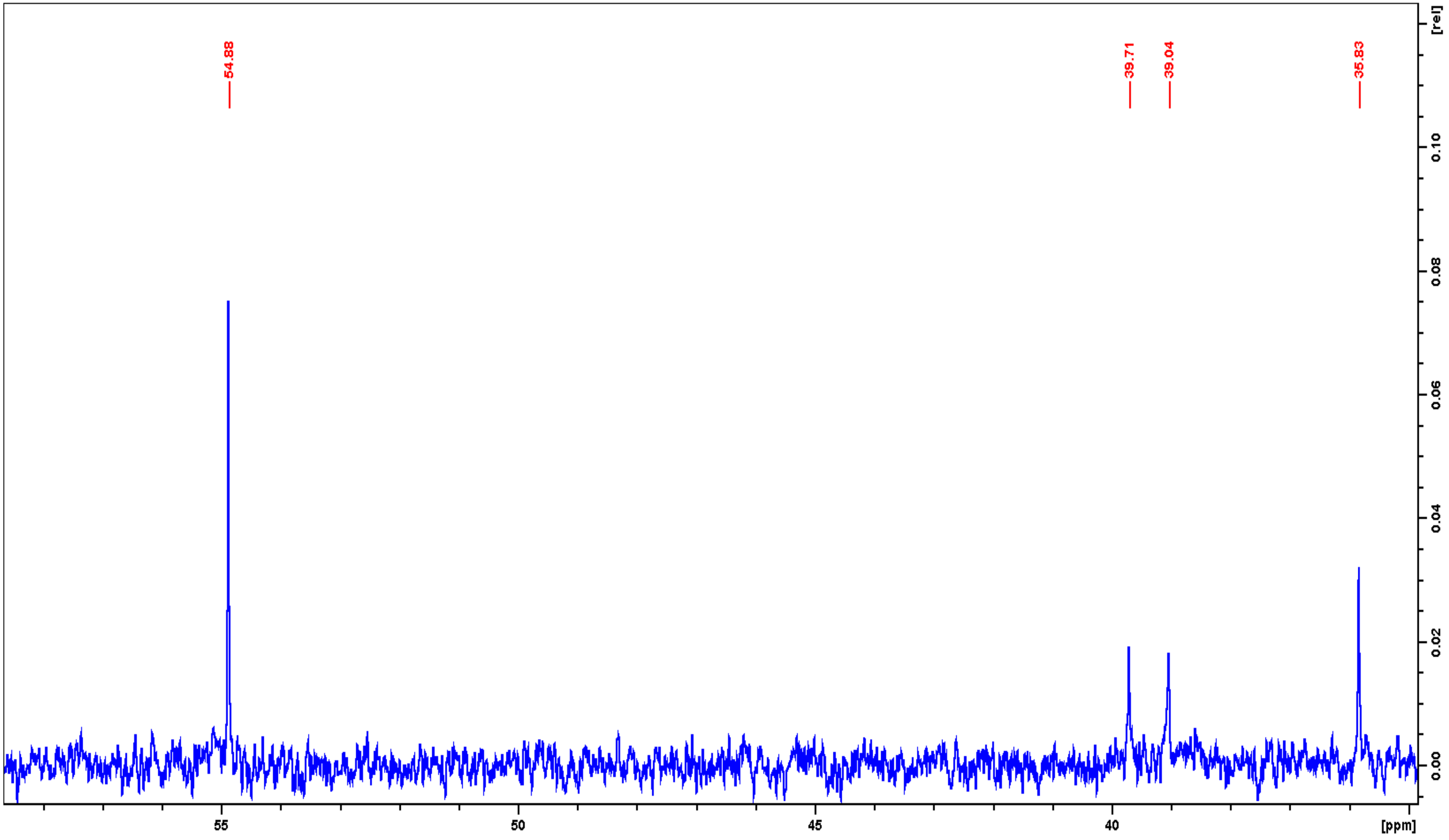


Figure S17. $^{13}$C NMR spectrum of compounds **1a and 1b** (enlarged).

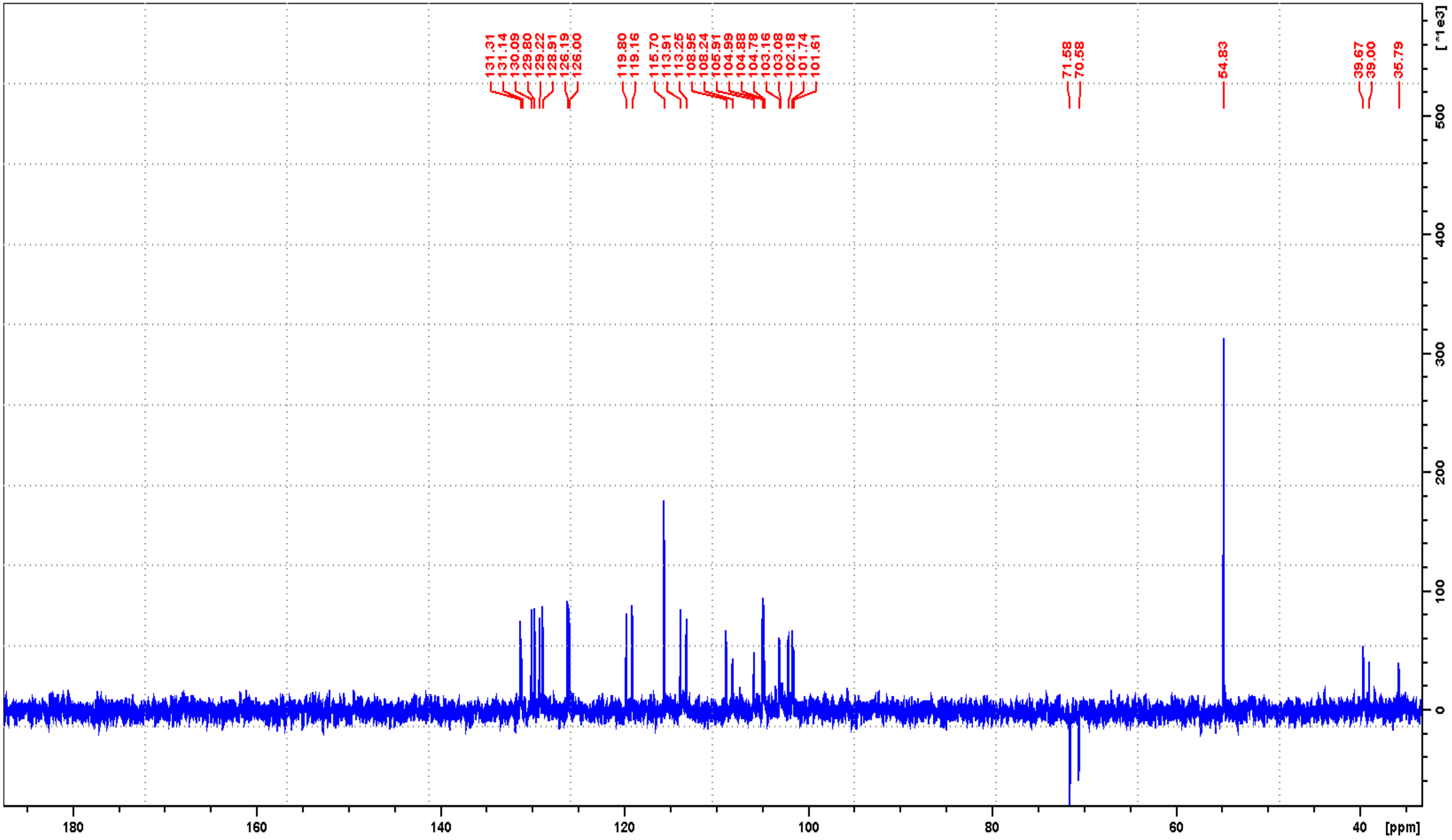


Figure S18. $^{13}$C dept135 NMR spectrum of compounds **1a and 1b**.

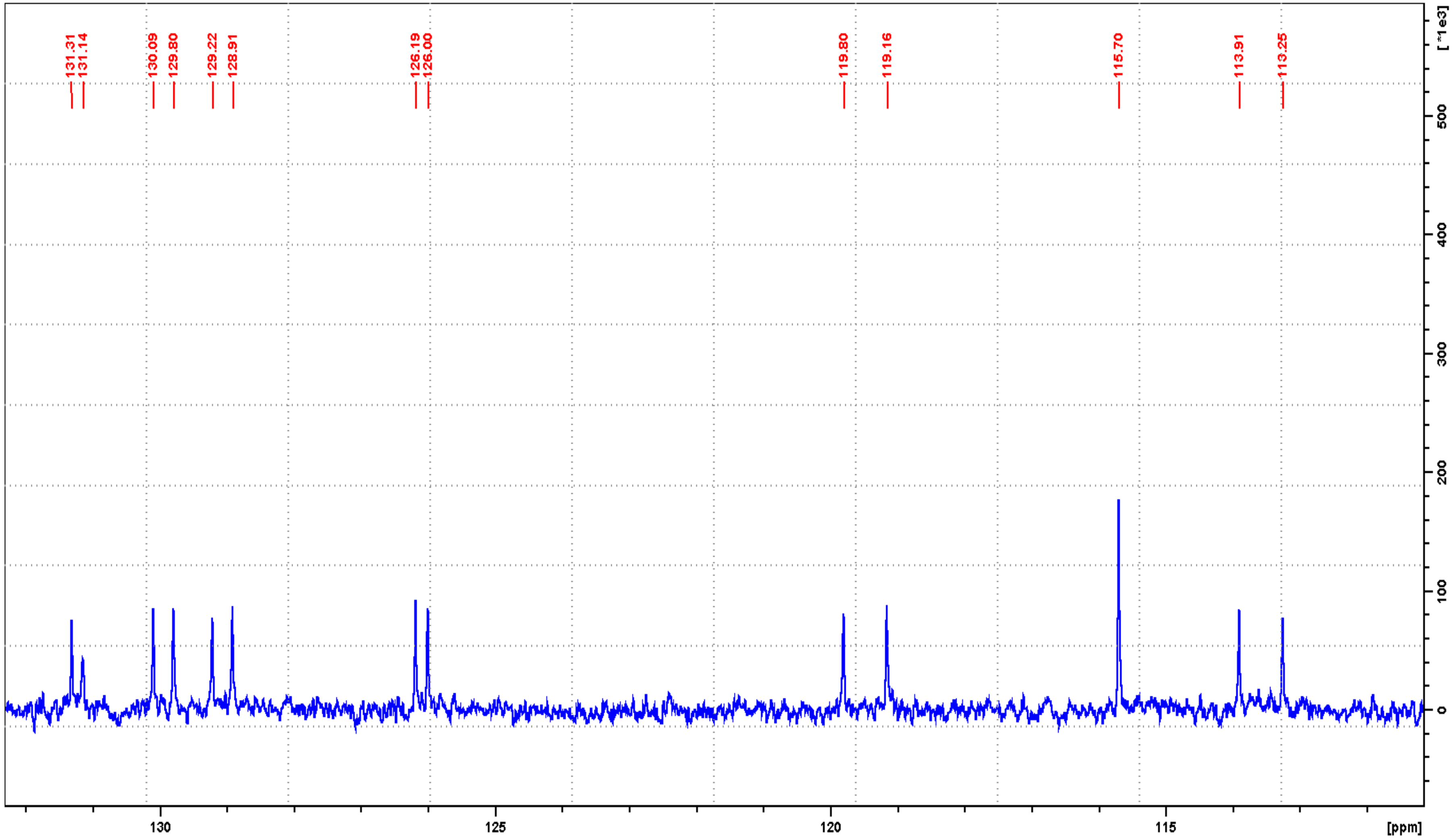


Figure S19. $^{13}C$ dept135 NMR spectrum of compounds **1a and 1b** (enlarged).

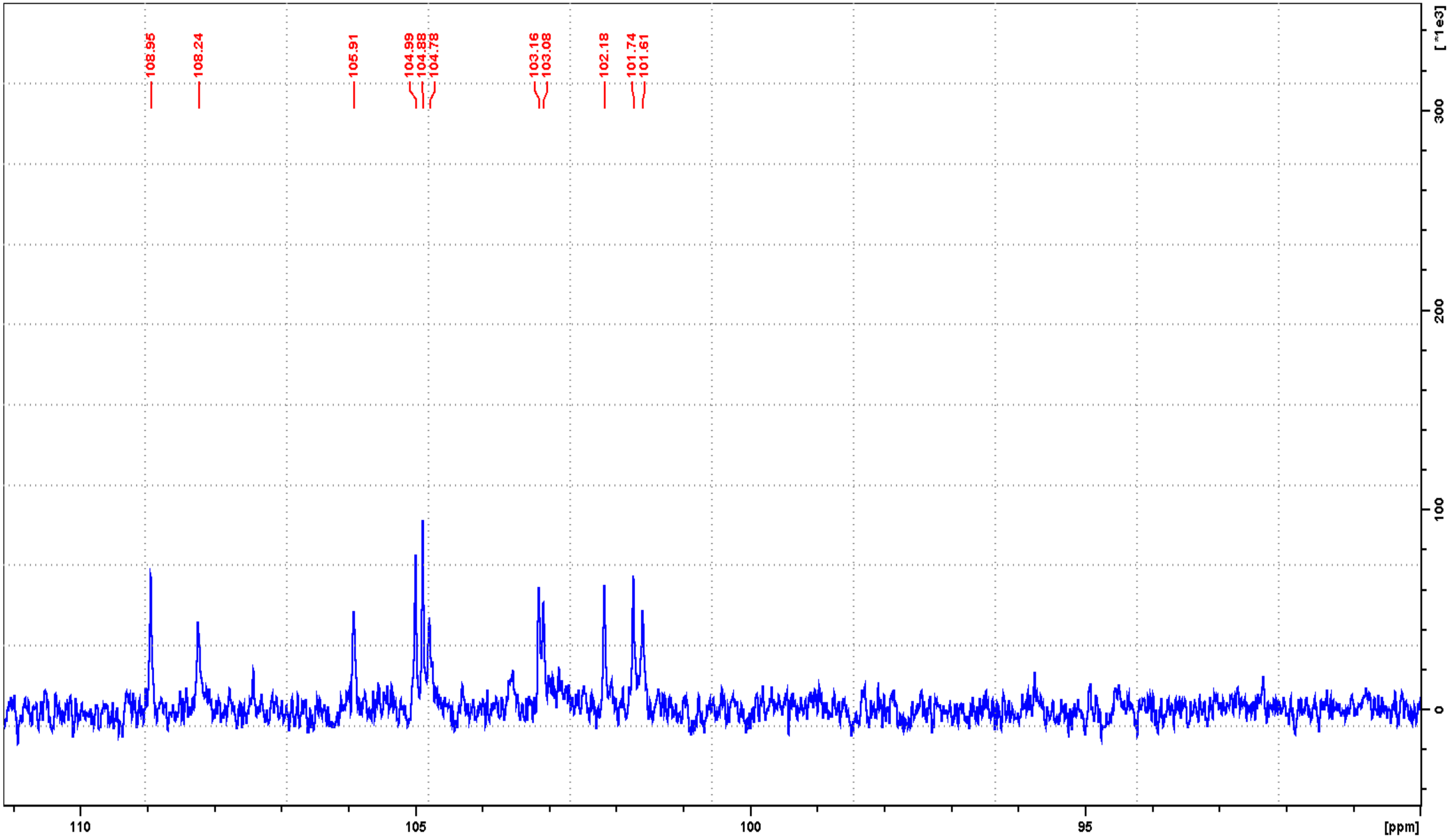


Figure S20. $^{13}$C dept135 NMR spectrum of compounds **1a and 1b** (enlarged).

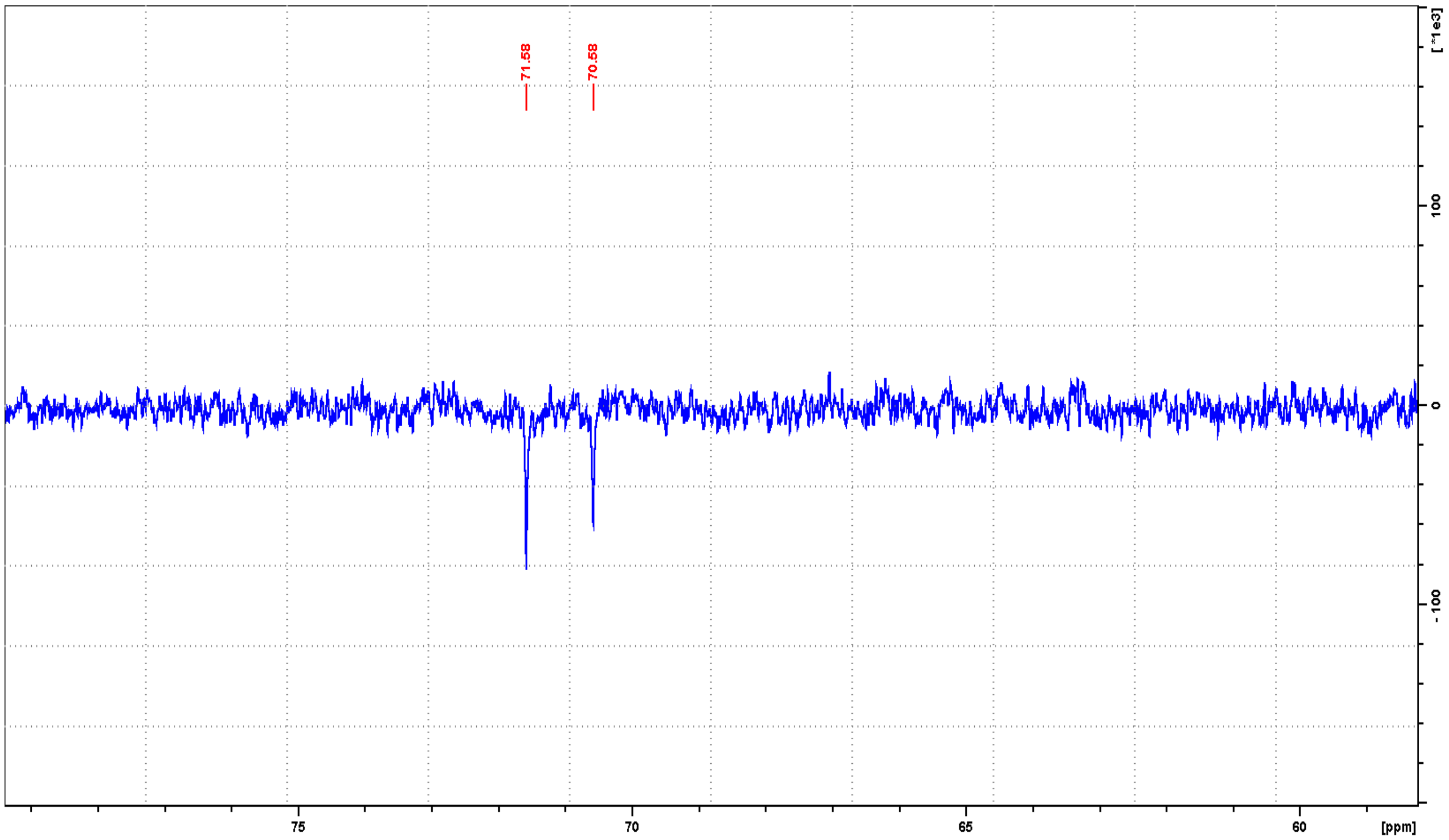


Figure S21. $^{13}C$ dept135 NMR spectrum of compounds **1a and 1b** (enlarged).

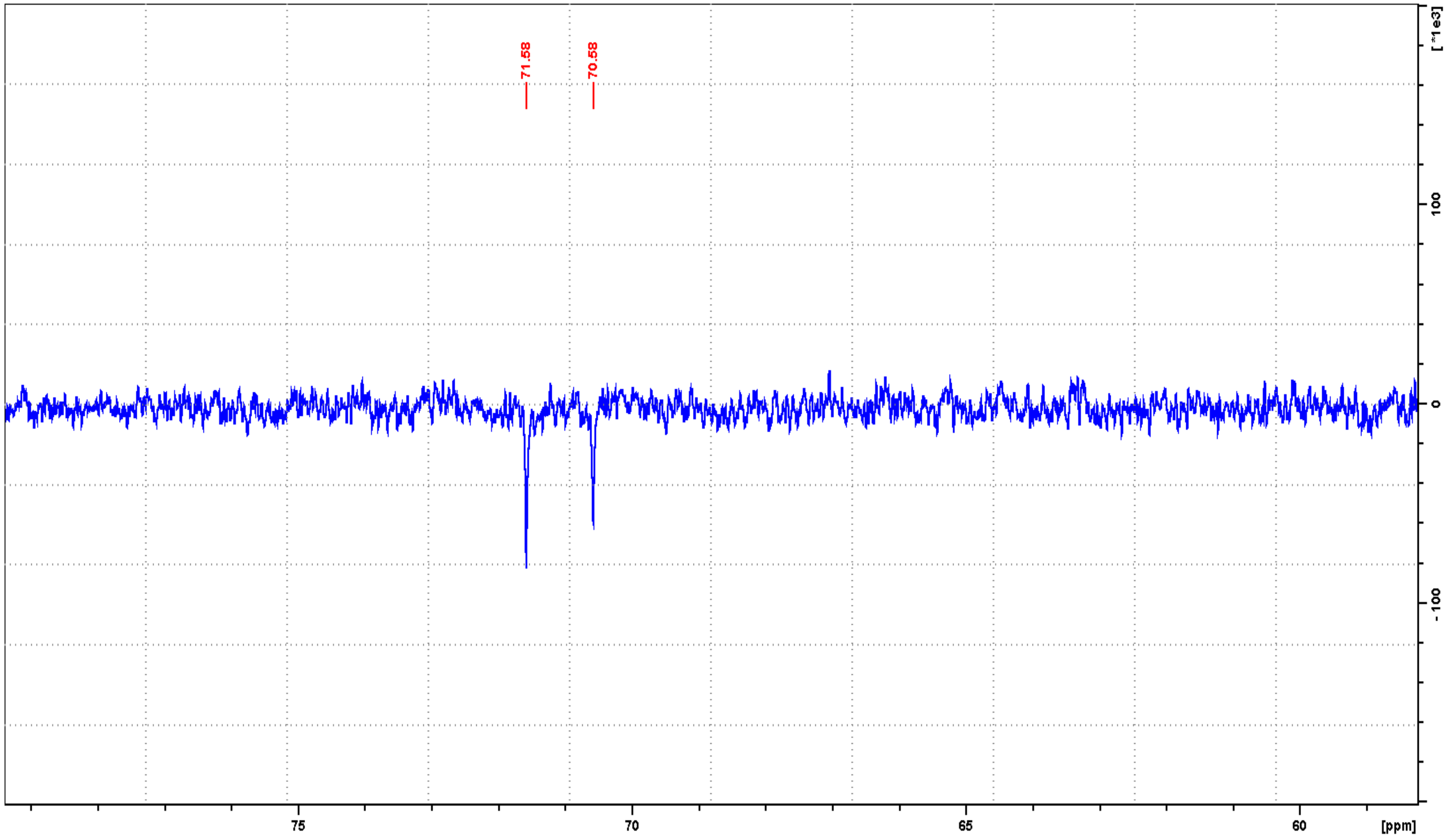


Figure S22. $^{13}C$ dept135 NMR spectrum of compounds **1a and 1b** (enlarged).

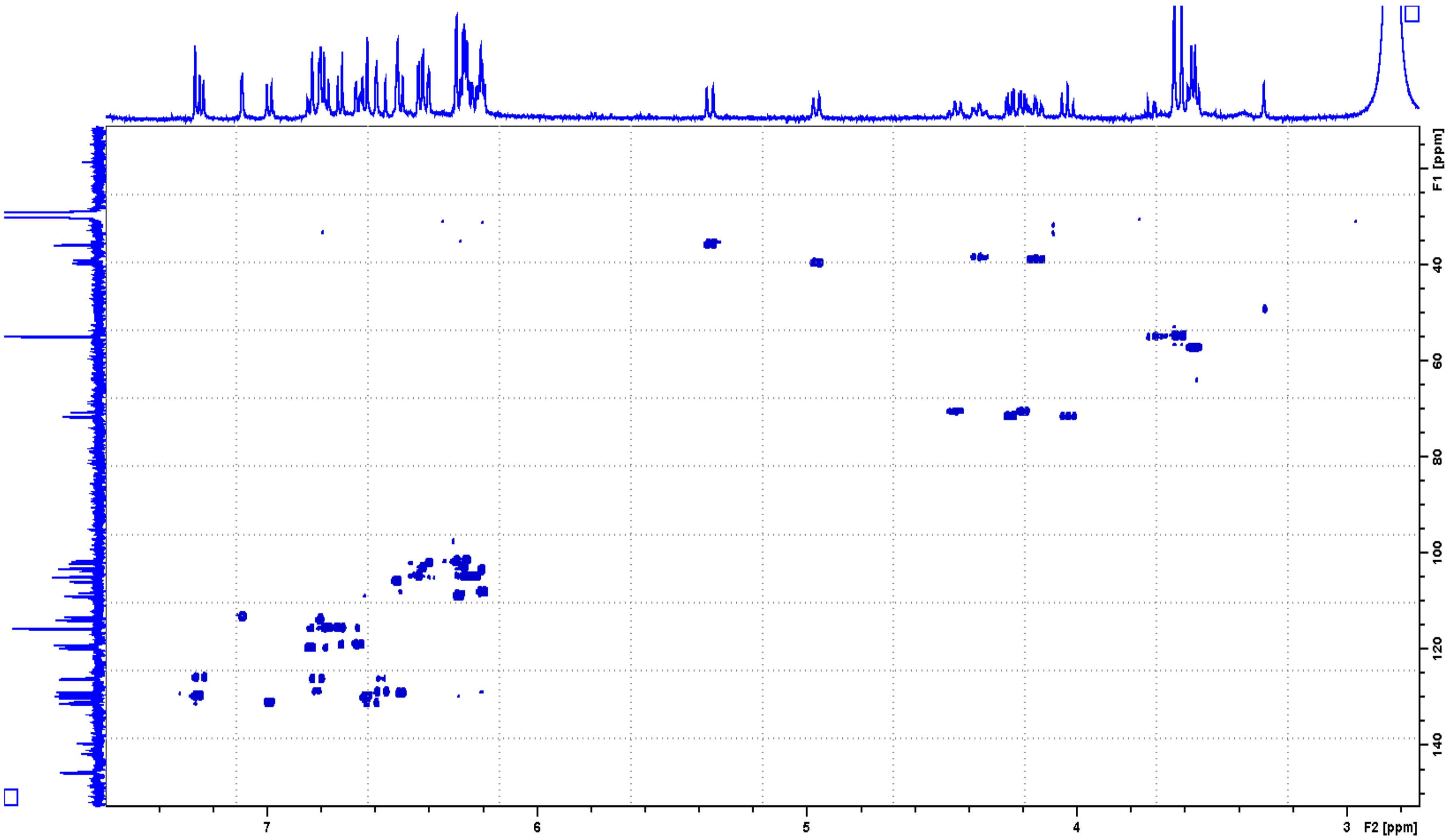


Figure S23. HSQC spectrum of compounds **1a and 1b**.

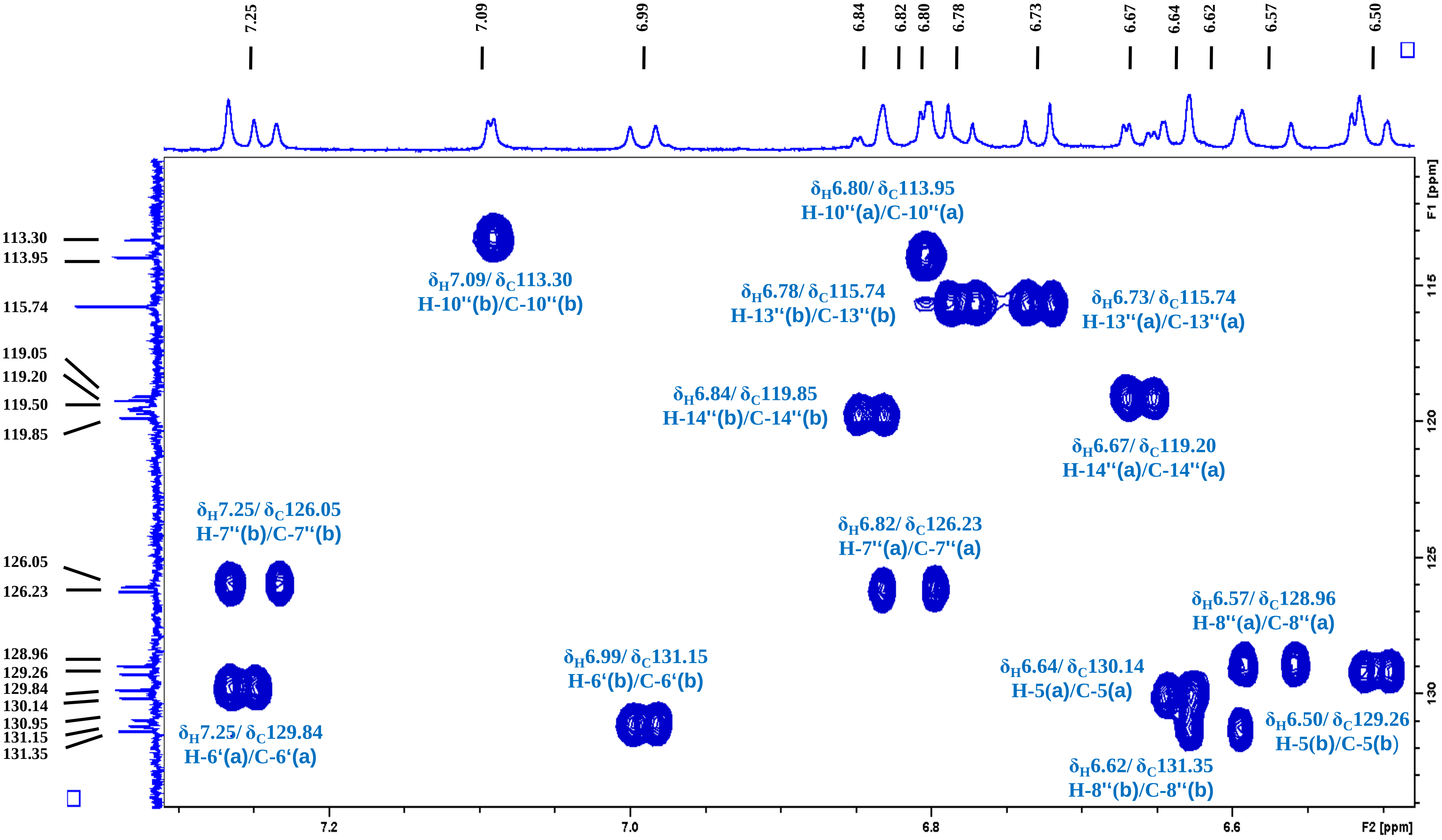


Figure S24. HSQC spectrum of compounds **1a and 1b** (enlarged).

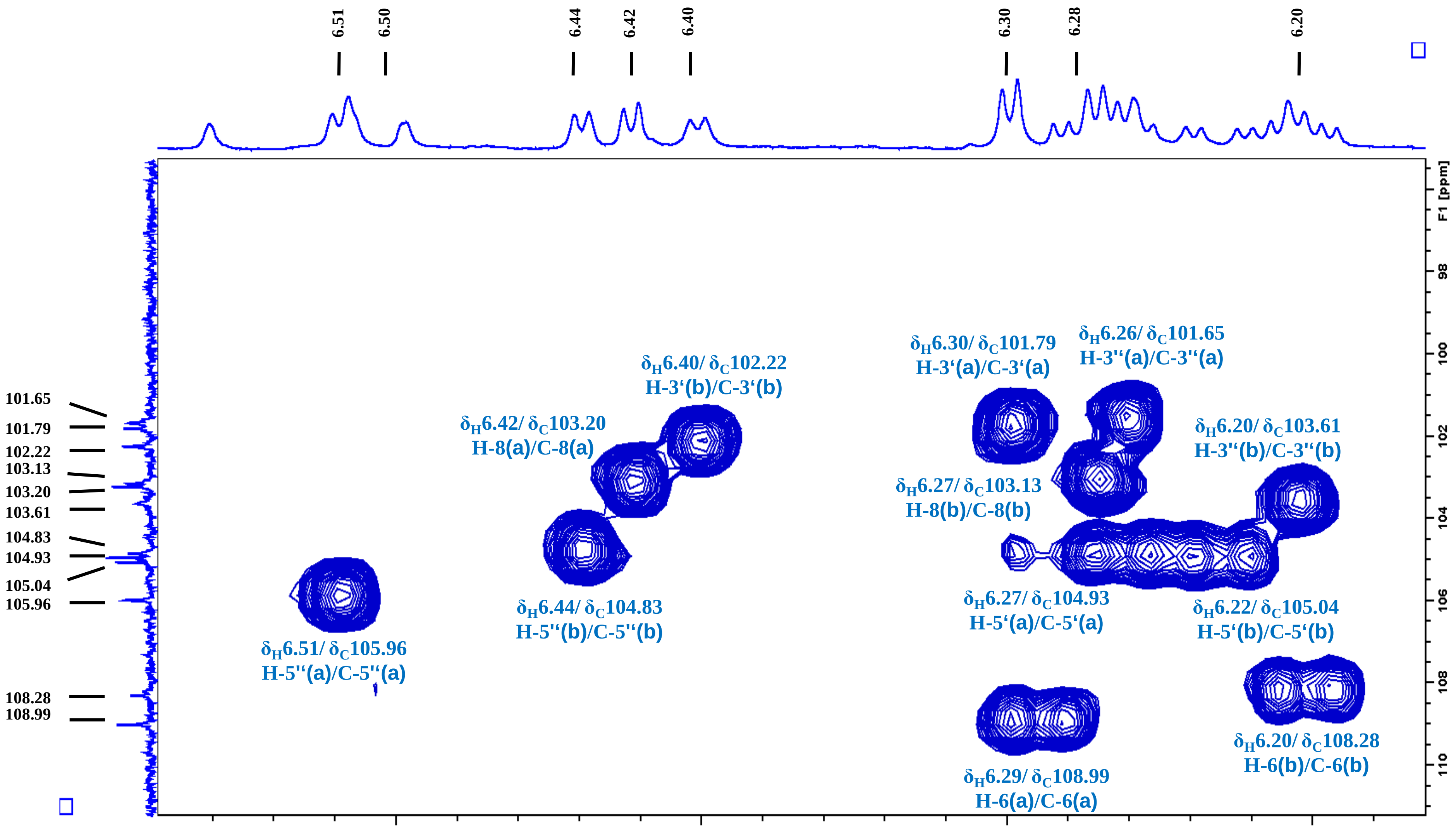


Figure S25. HSQC spectrum of compounds **1a and 1b** (enlarged).

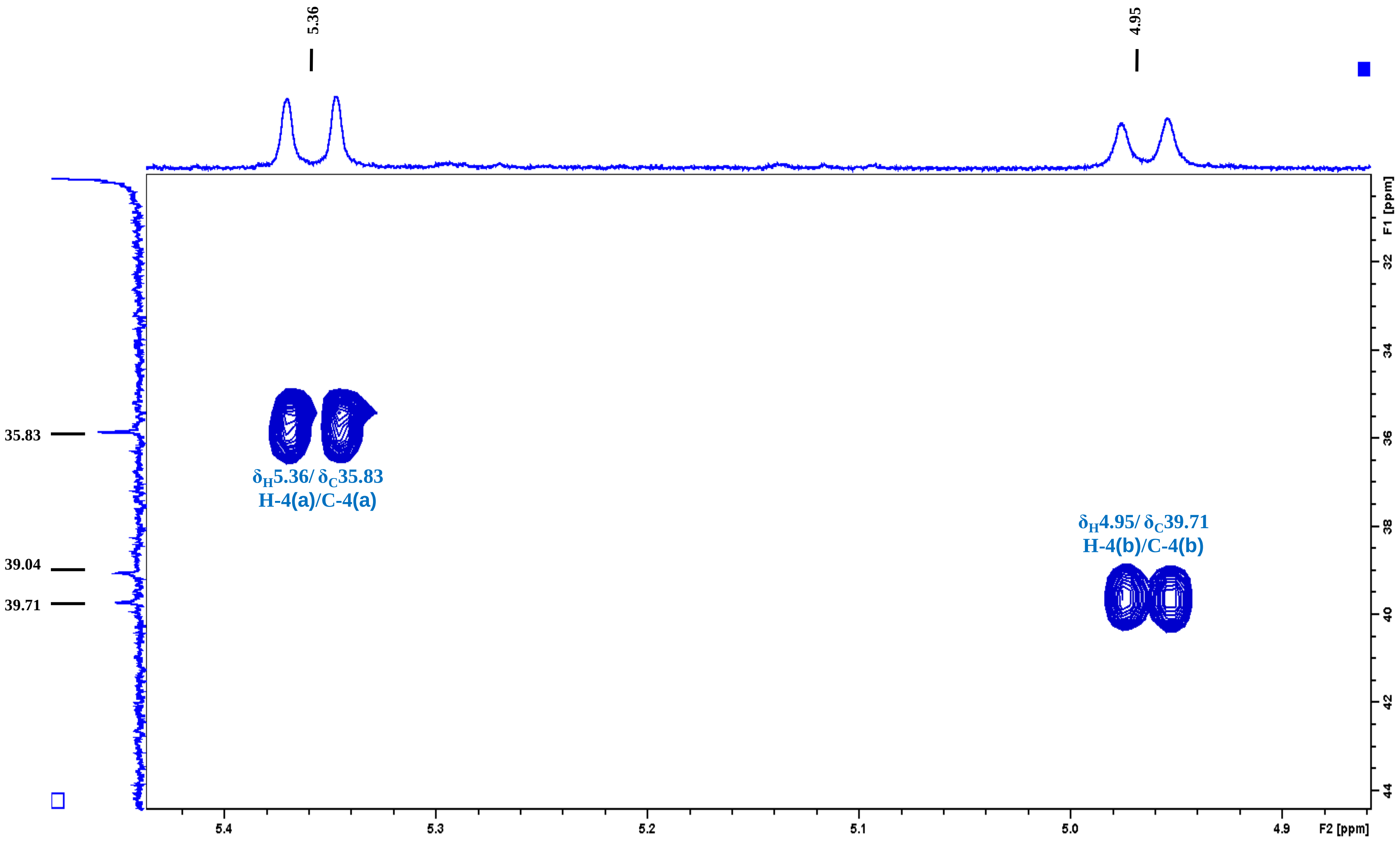


Figure S26. HSQC spectrum of compounds **1a and 1b** (enlarged).

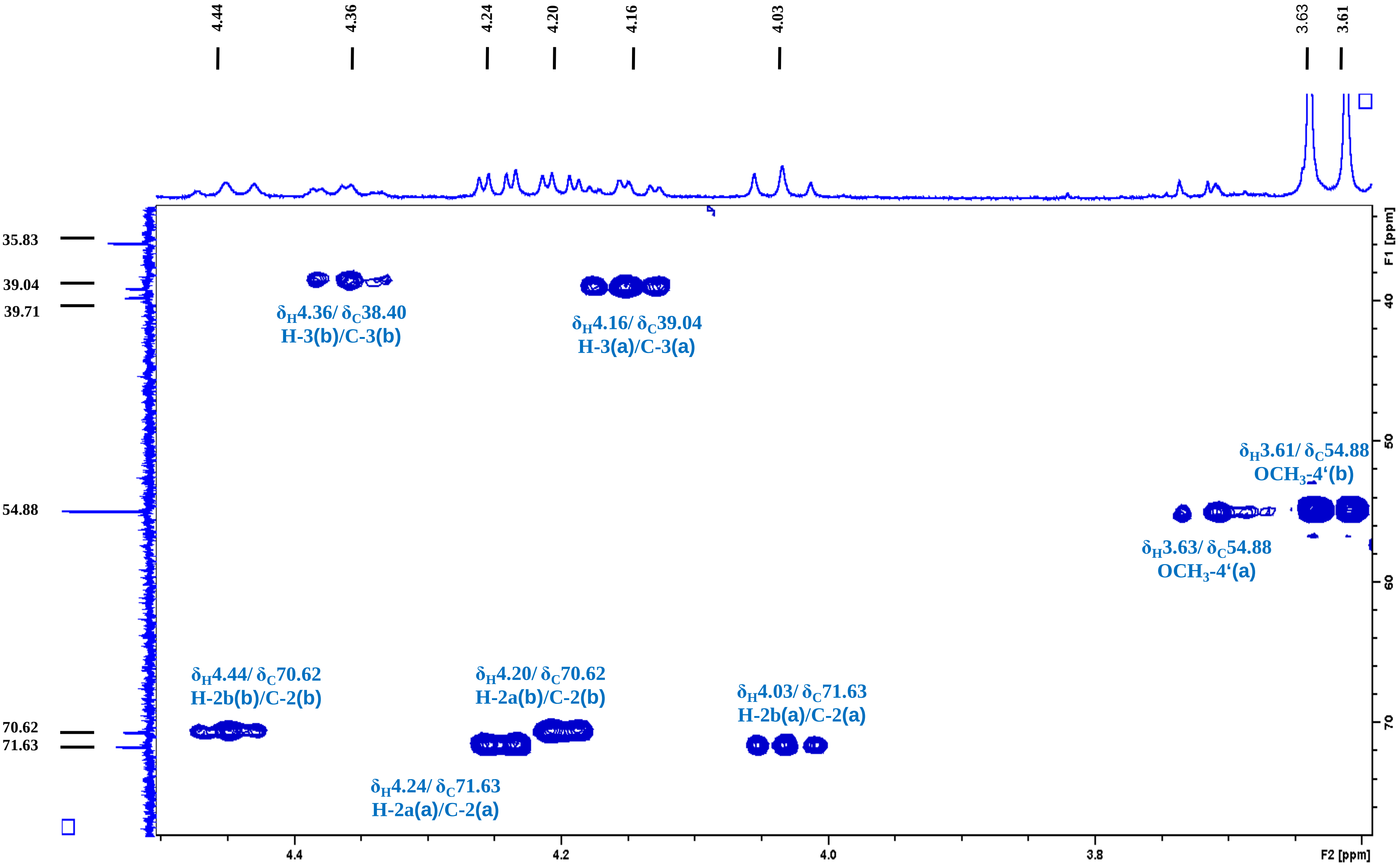


Figure S27. HSQC spectrum of compounds **1a and 1b** (enlarged).

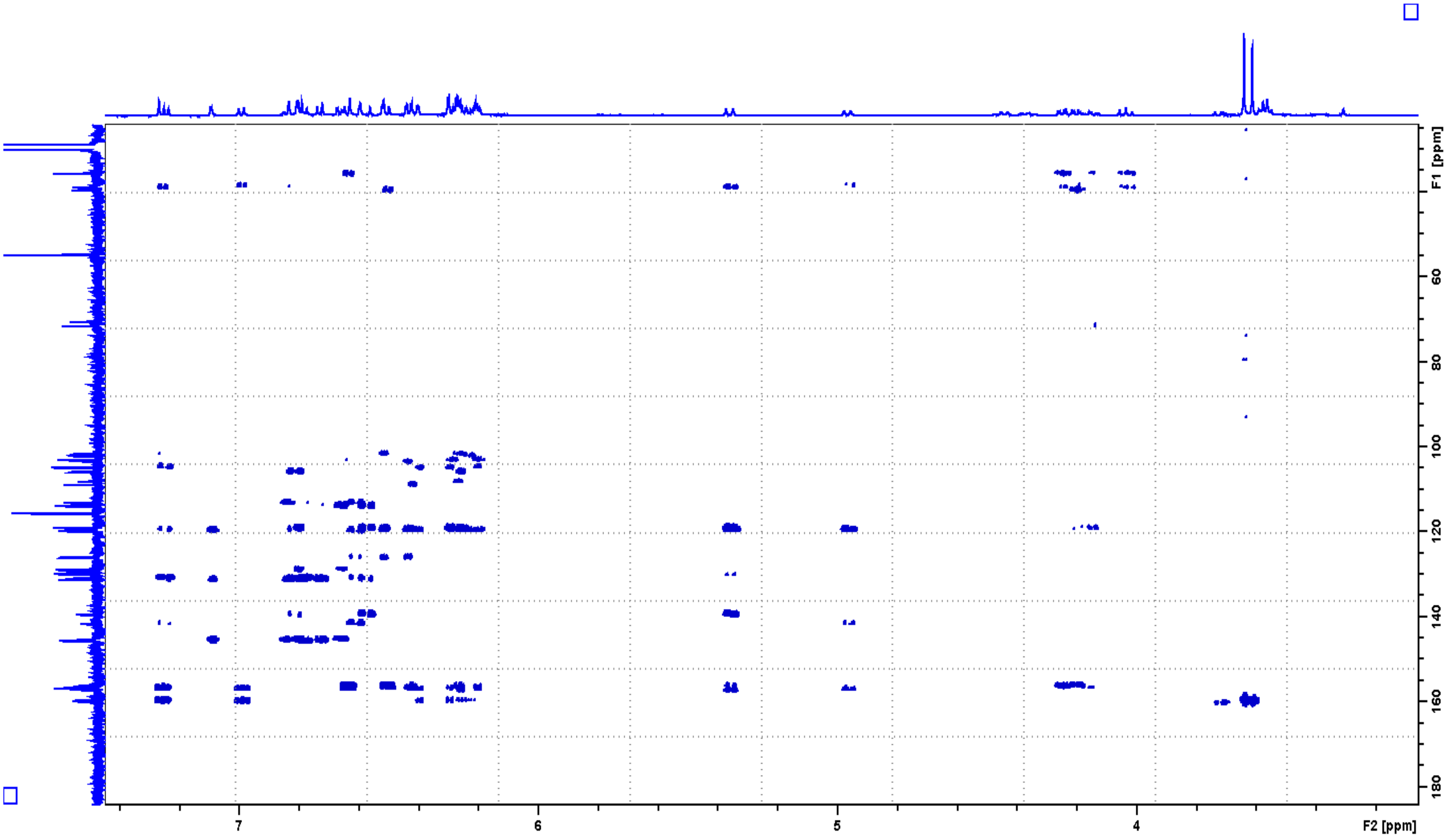


Figure S28. HMBC spectrum of compounds **1a and 1b**.

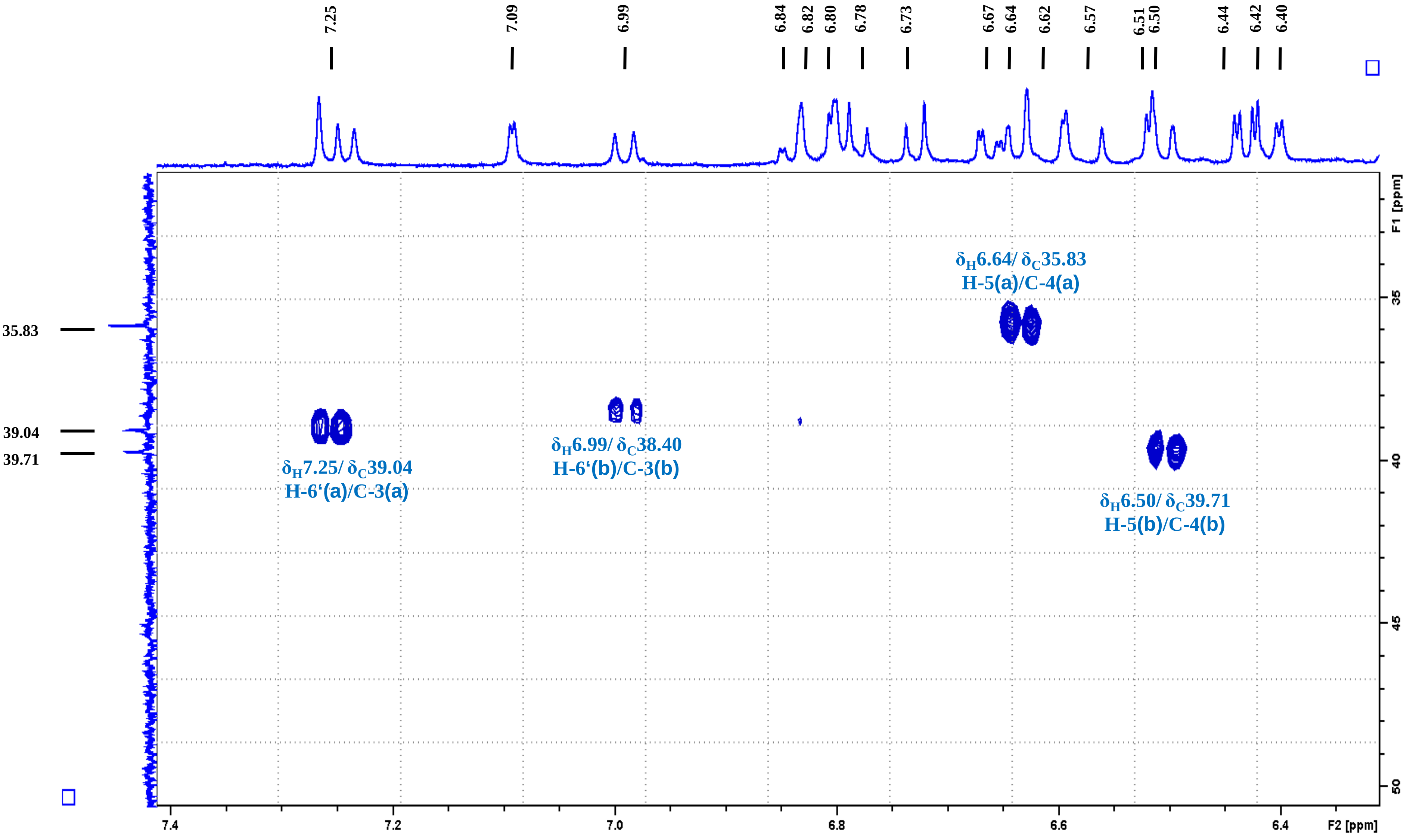


Figure S29. HMBC spectrum of compounds **1a and 1b** (enlarged).

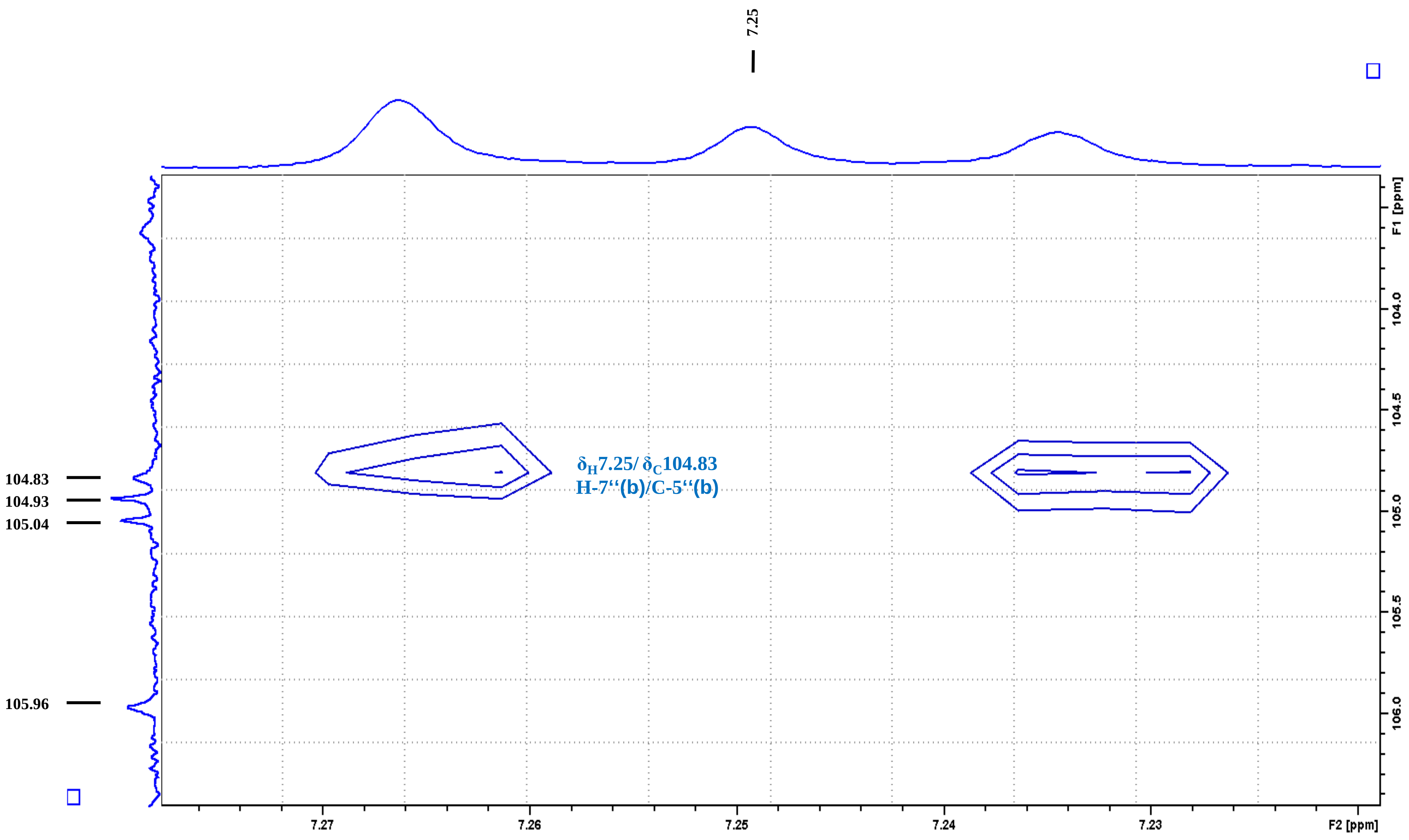


Figure S30. HMBC spectrum of compounds **1a and 1b** (enlarged).

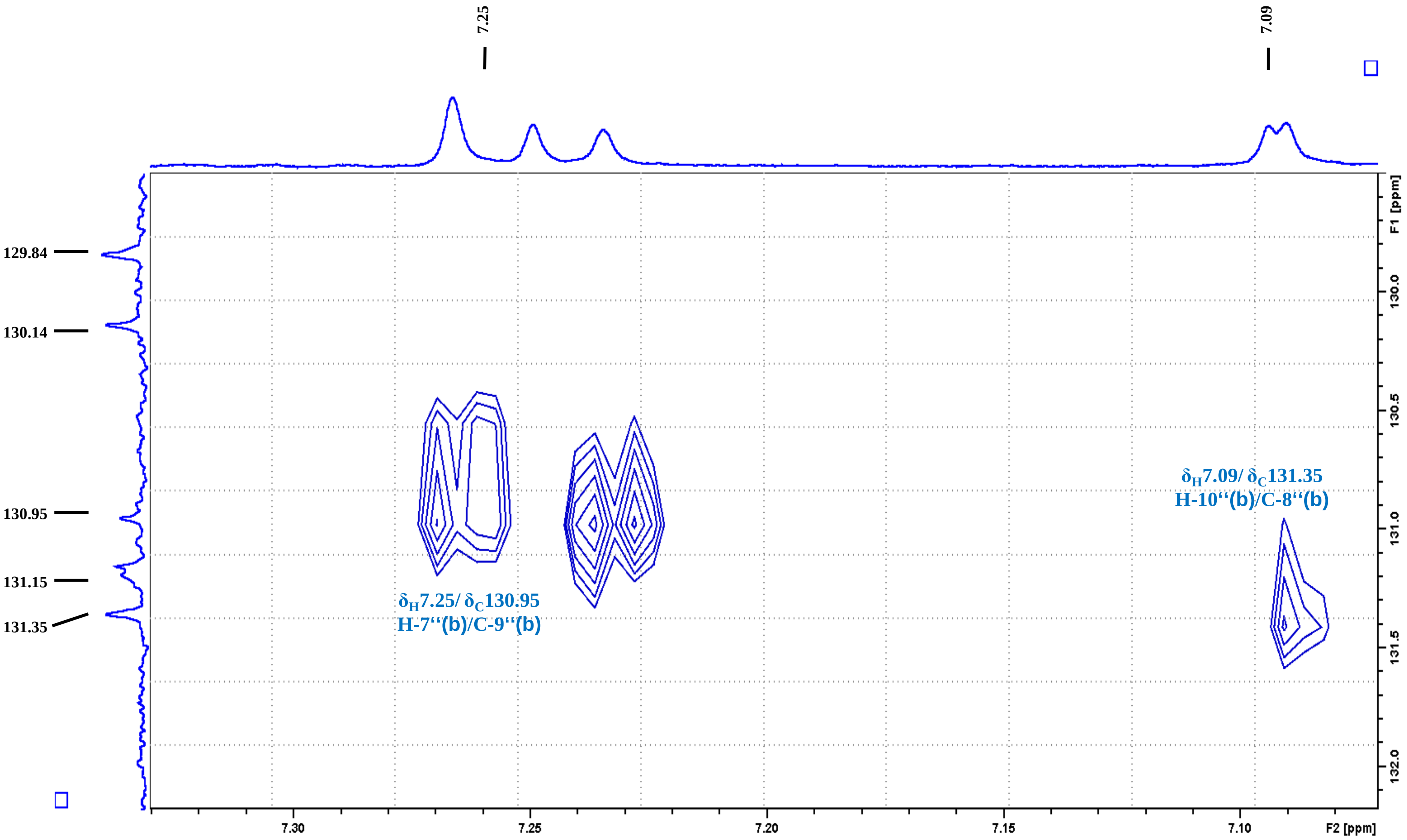


Figure S31. HMBC spectrum of compounds **1a and 1b** (enlarged).

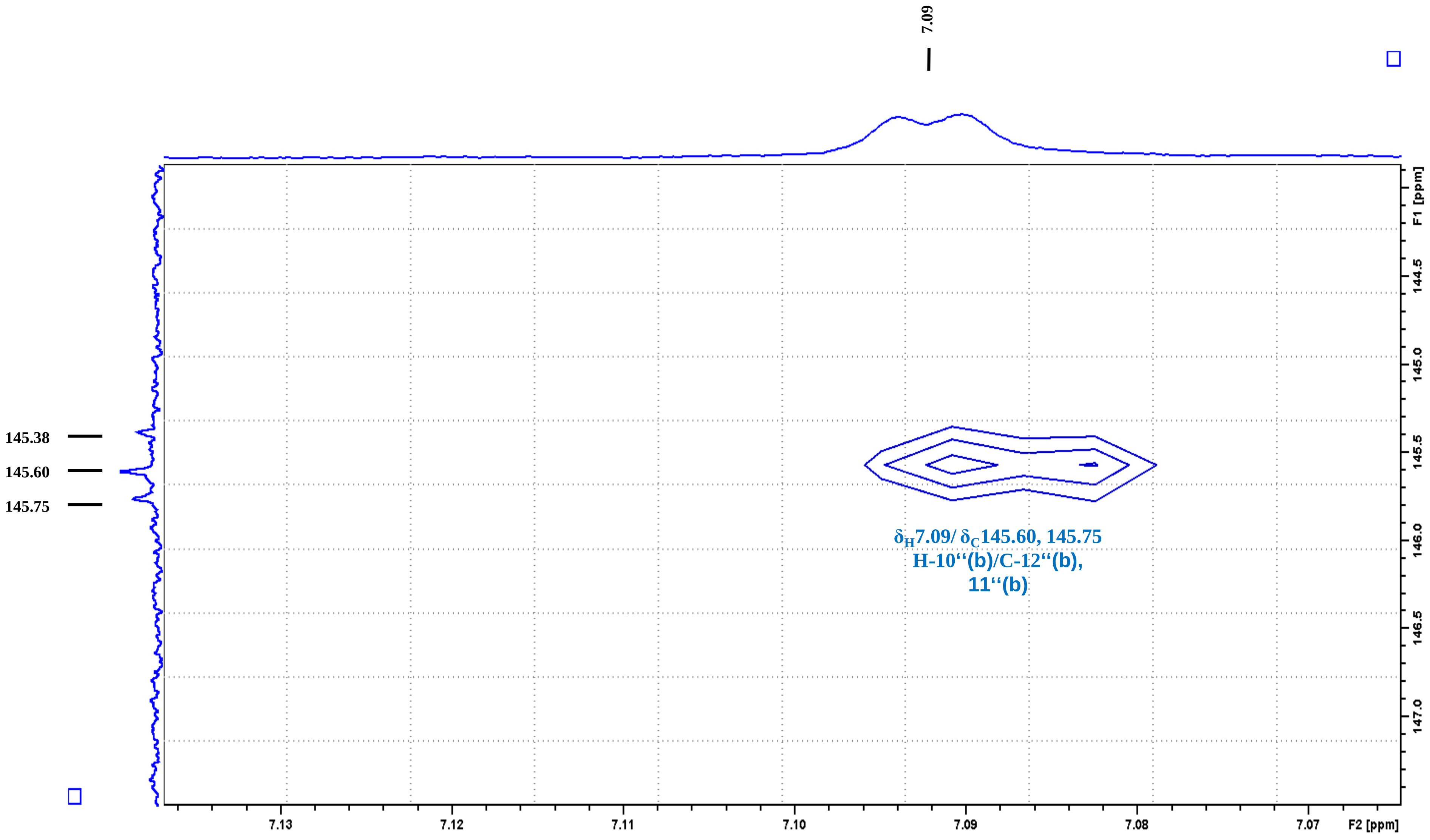


Figure S32. HMBC spectrum of compounds **1a and 1b** (enlarged).

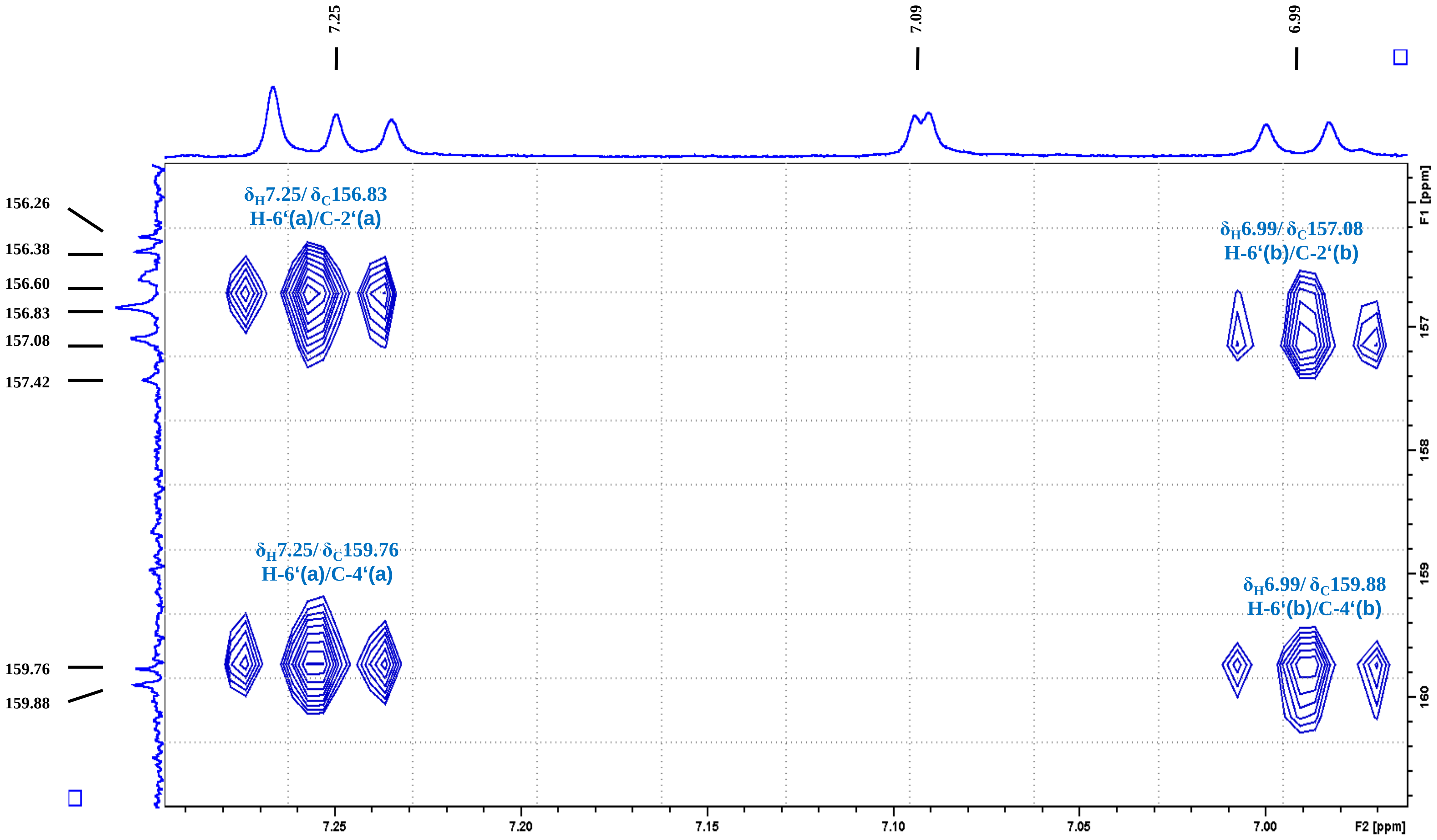


Figure S33. HMBC spectrum of compounds **1a and 1b** (enlarged).

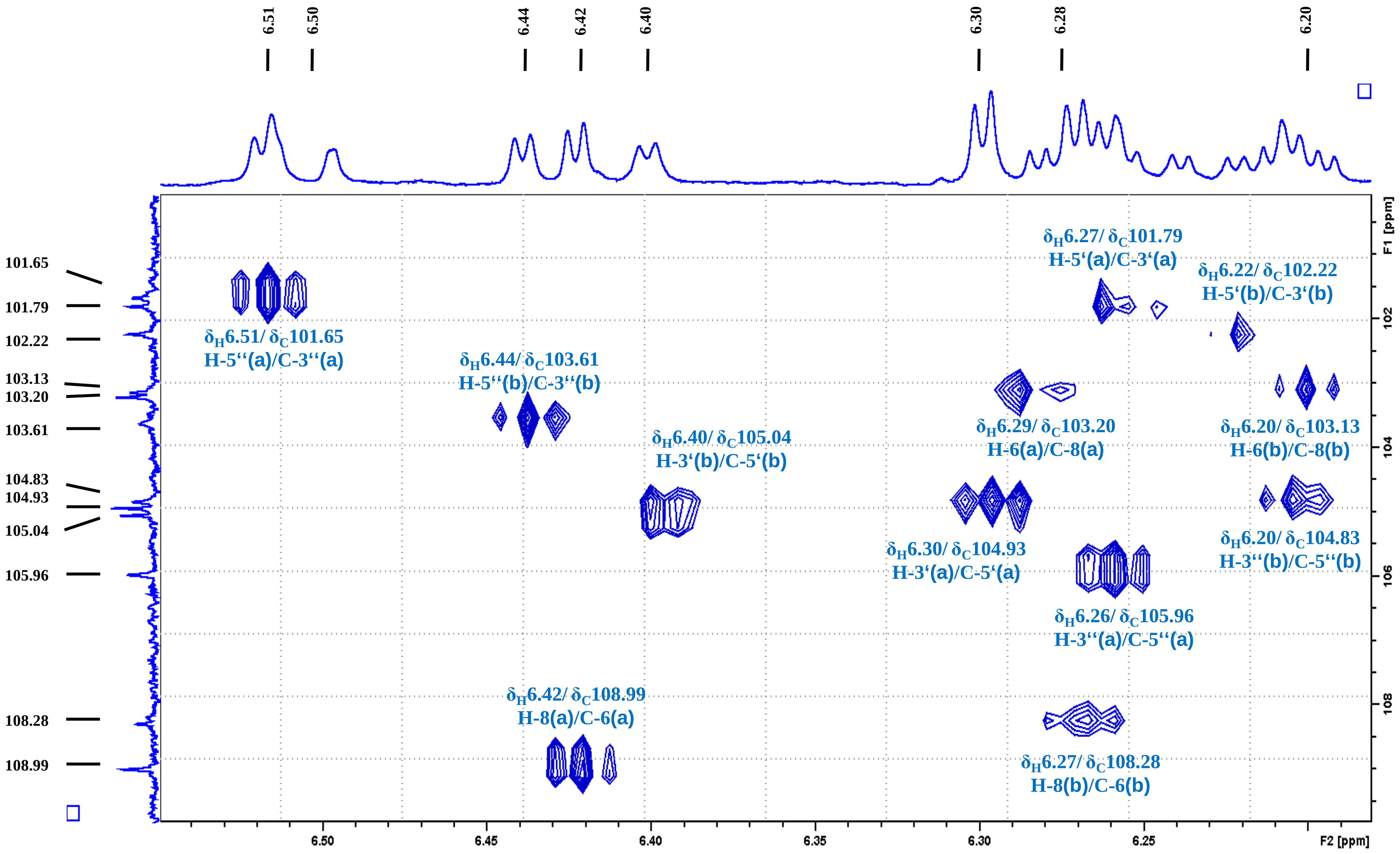


Figure S34. HMBC spectrum of compounds **1a and 1b** (enlarged).

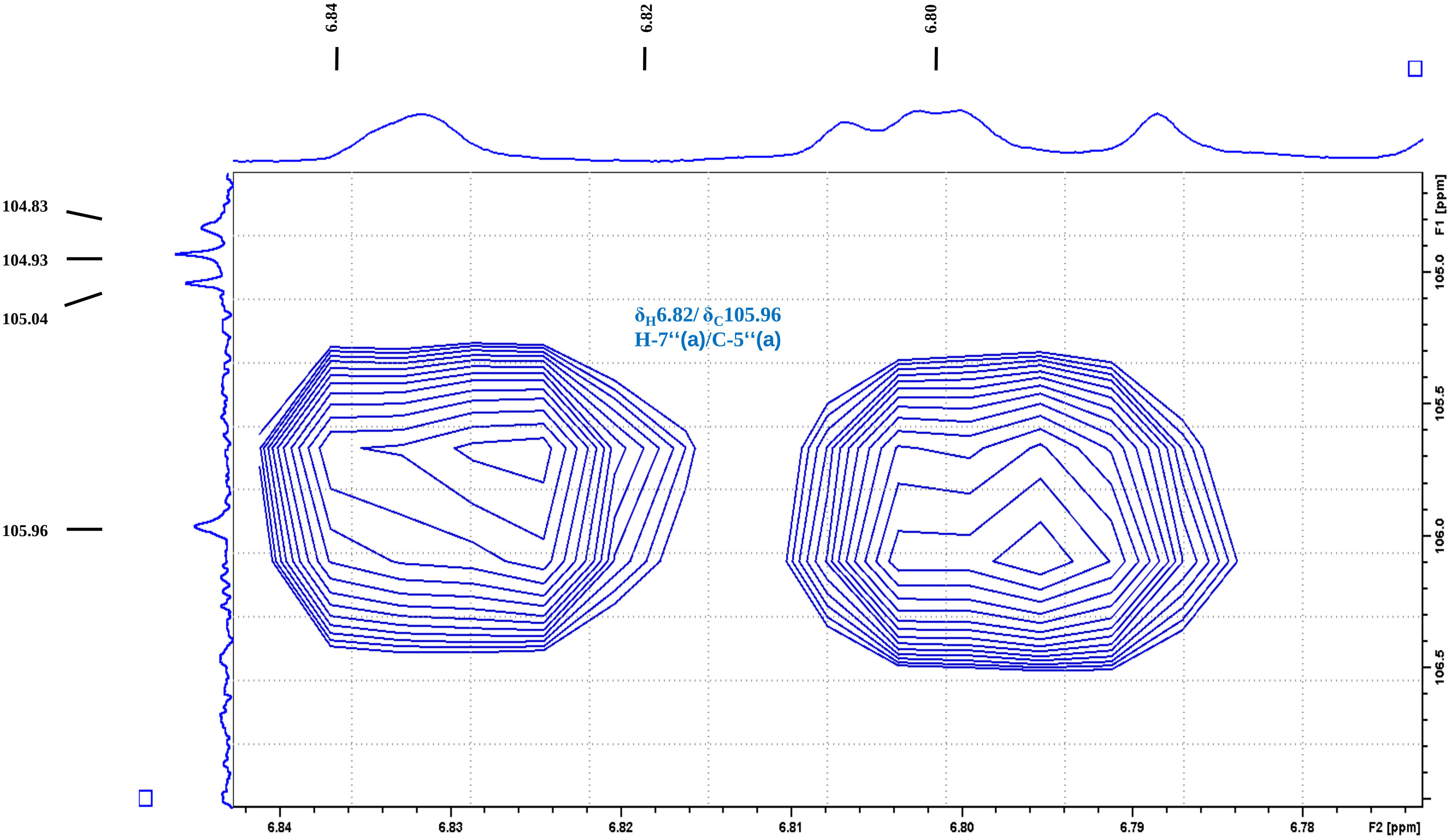


Figure S35. HMBC spectrum of compounds **1a and 1b** (enlarged).

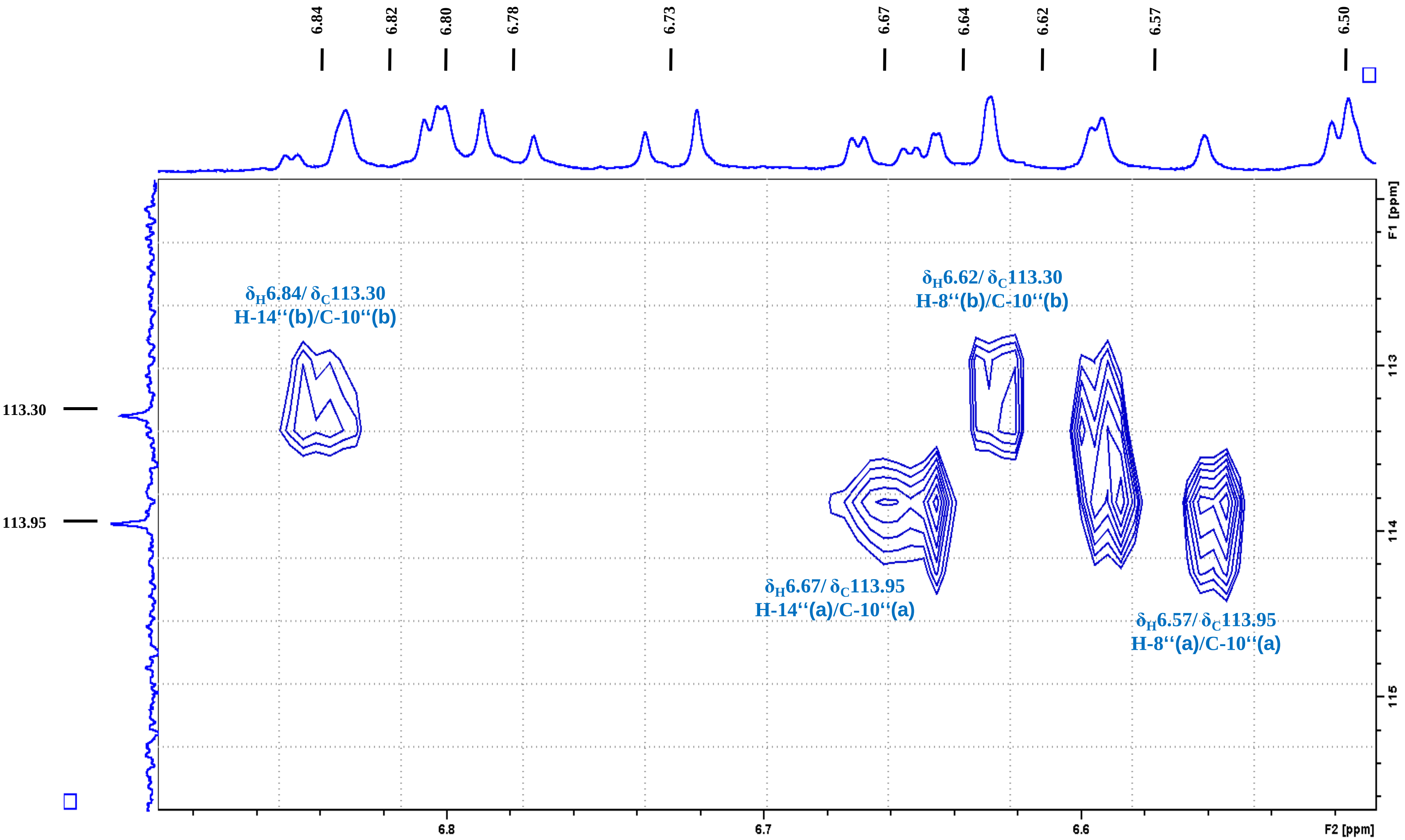


Figure S36. HMBC spectrum of compounds **1a and 1b** (enlarged).

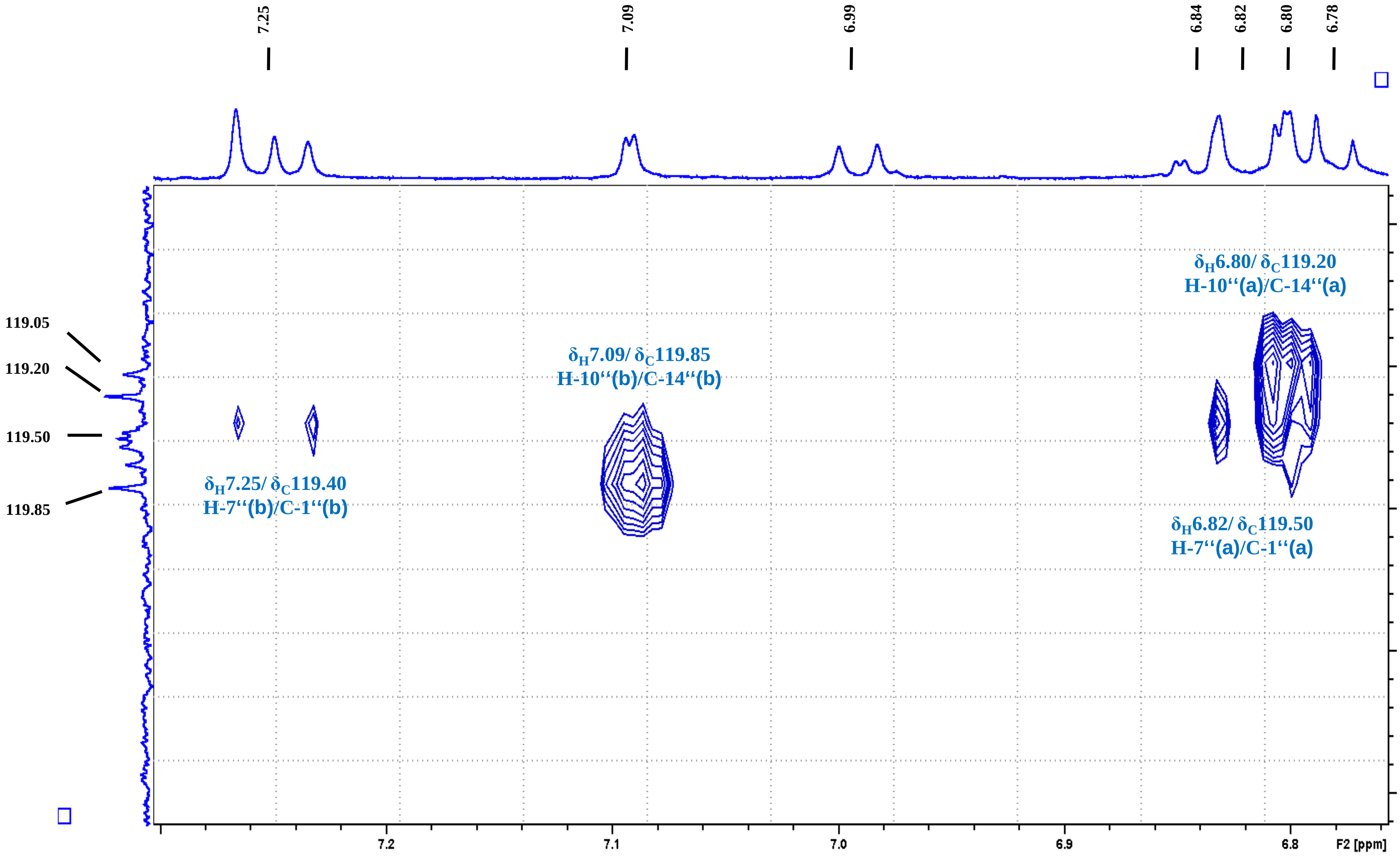


Figure S37. HMBC spectrum of compounds **1a and 1b** (enlarged).

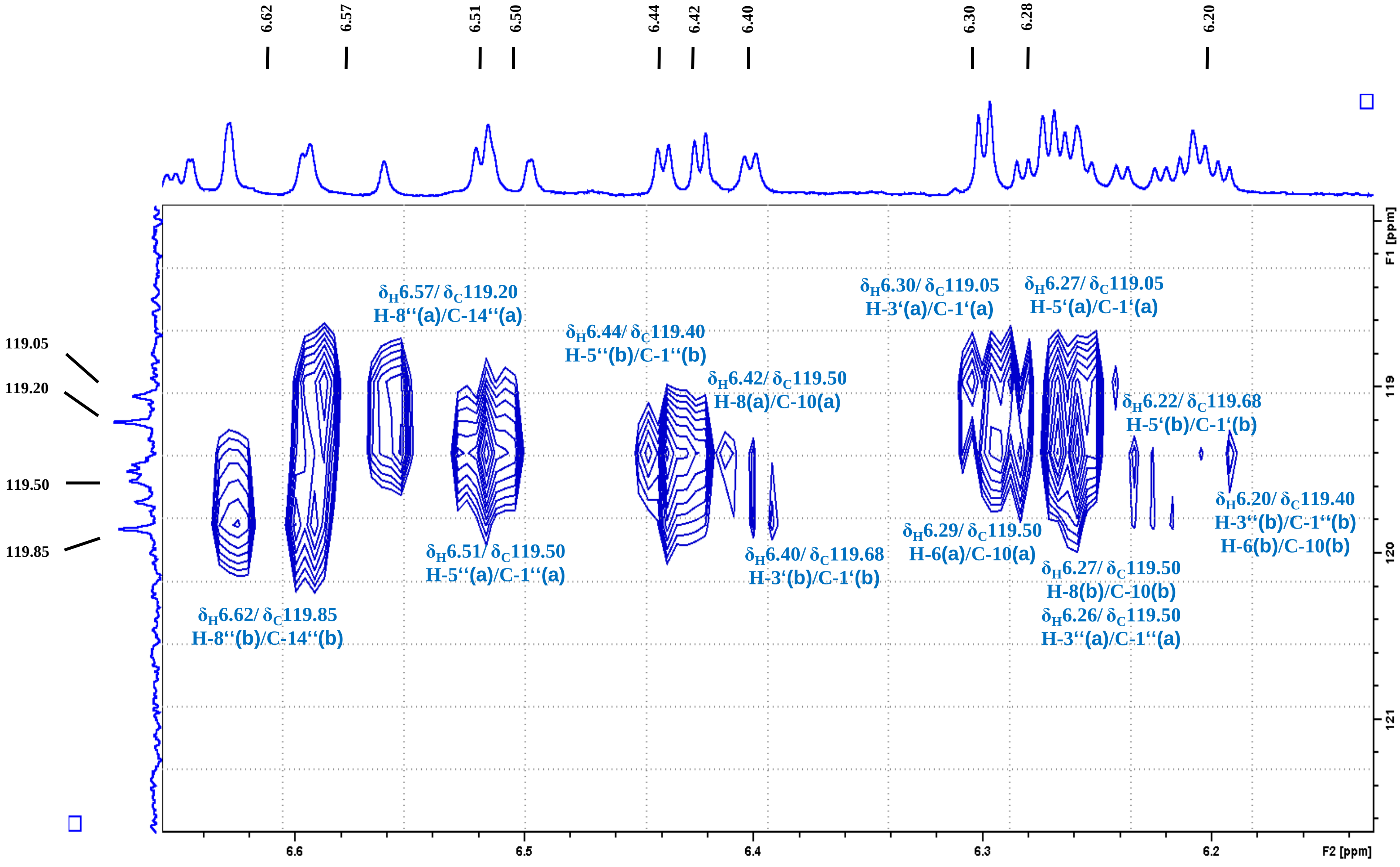


Figure S38. HMBC spectrum of compounds **1a and 1b** (enlarged).

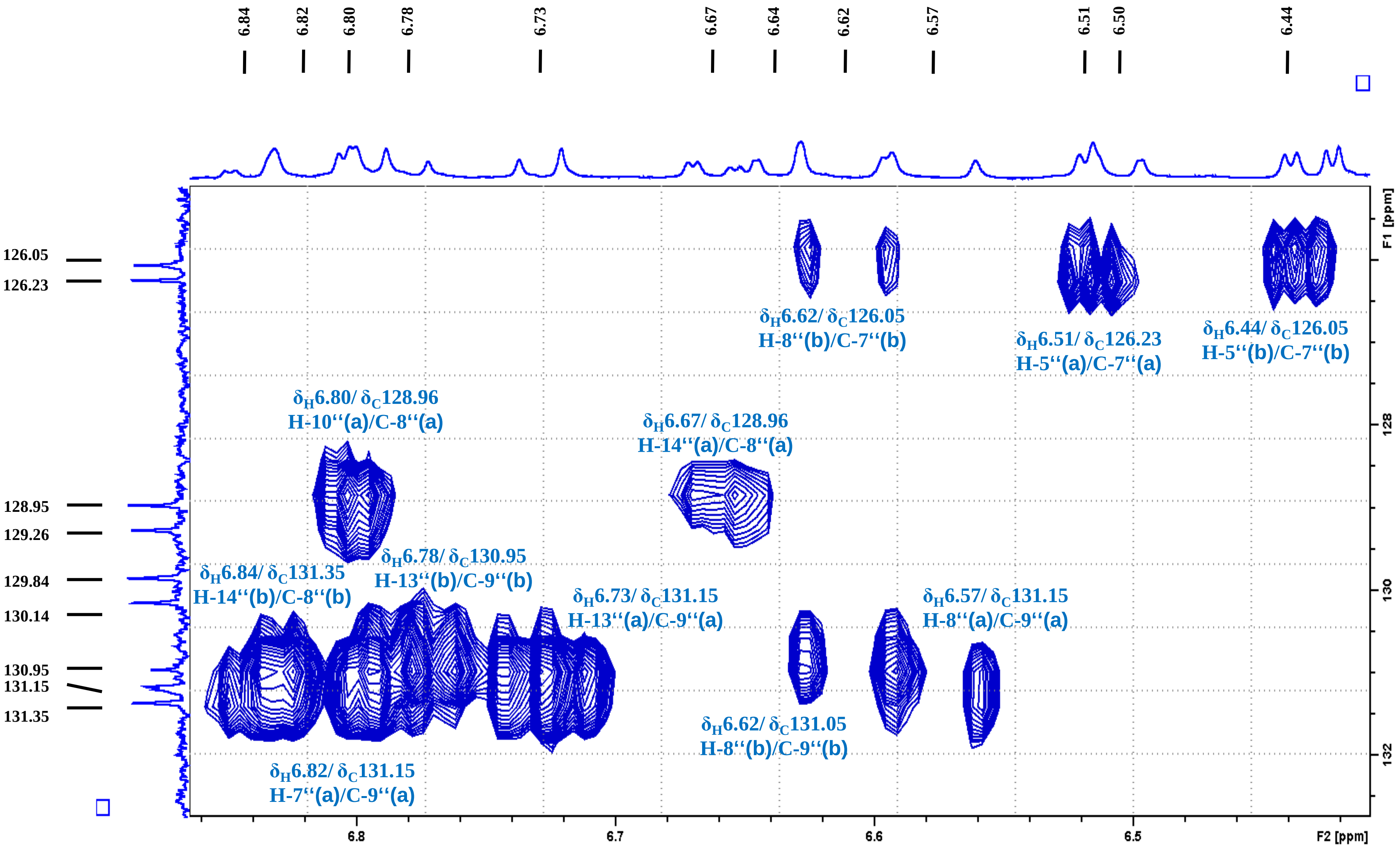


Figure S39. HMBC spectrum of compounds **1a and 1b** (enlarged).

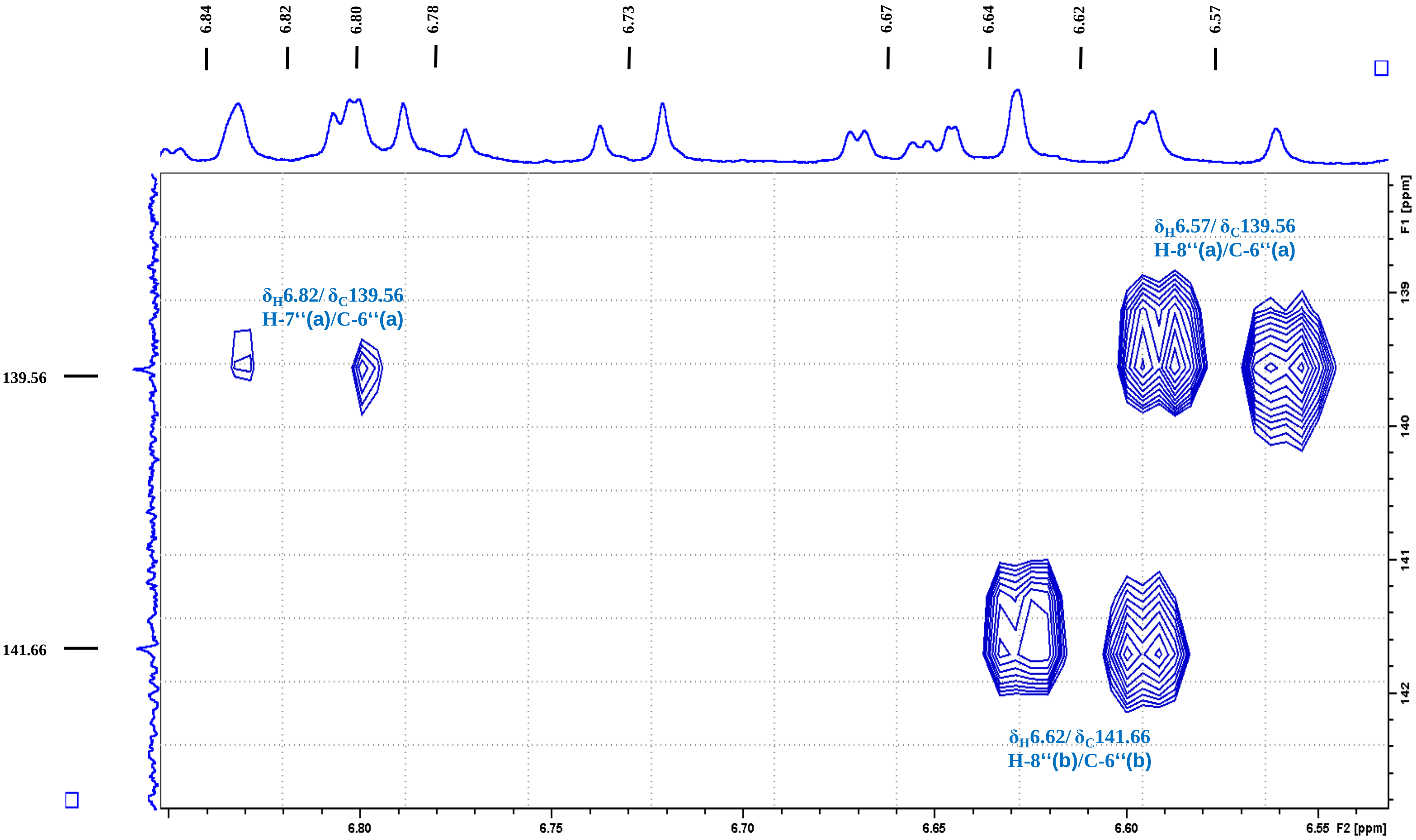


Figure S40. HMBC spectrum of compounds **1a and 1b** (enlarged).

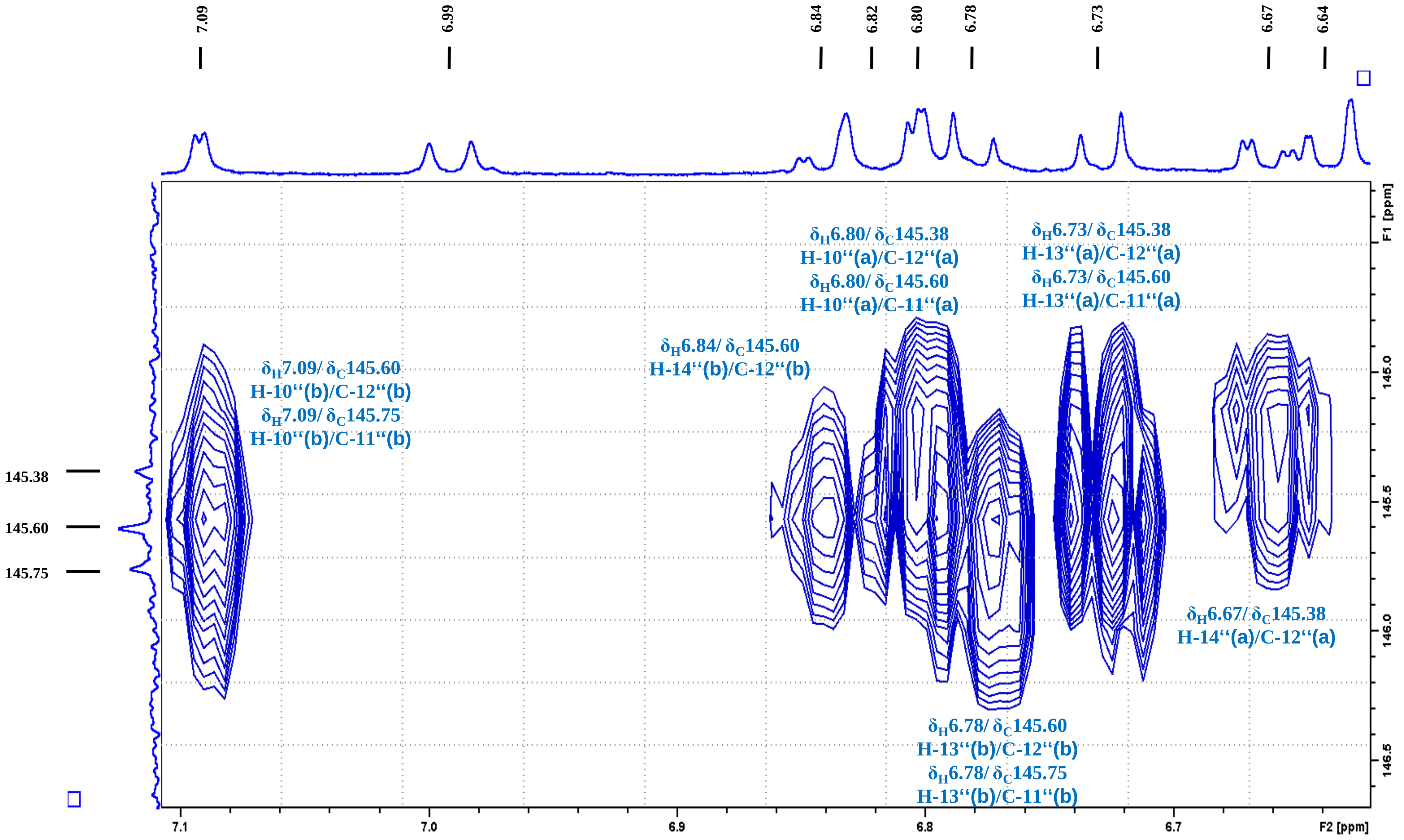


Figure S41. HMBC spectrum of compounds **1a and 1b** (enlarged).

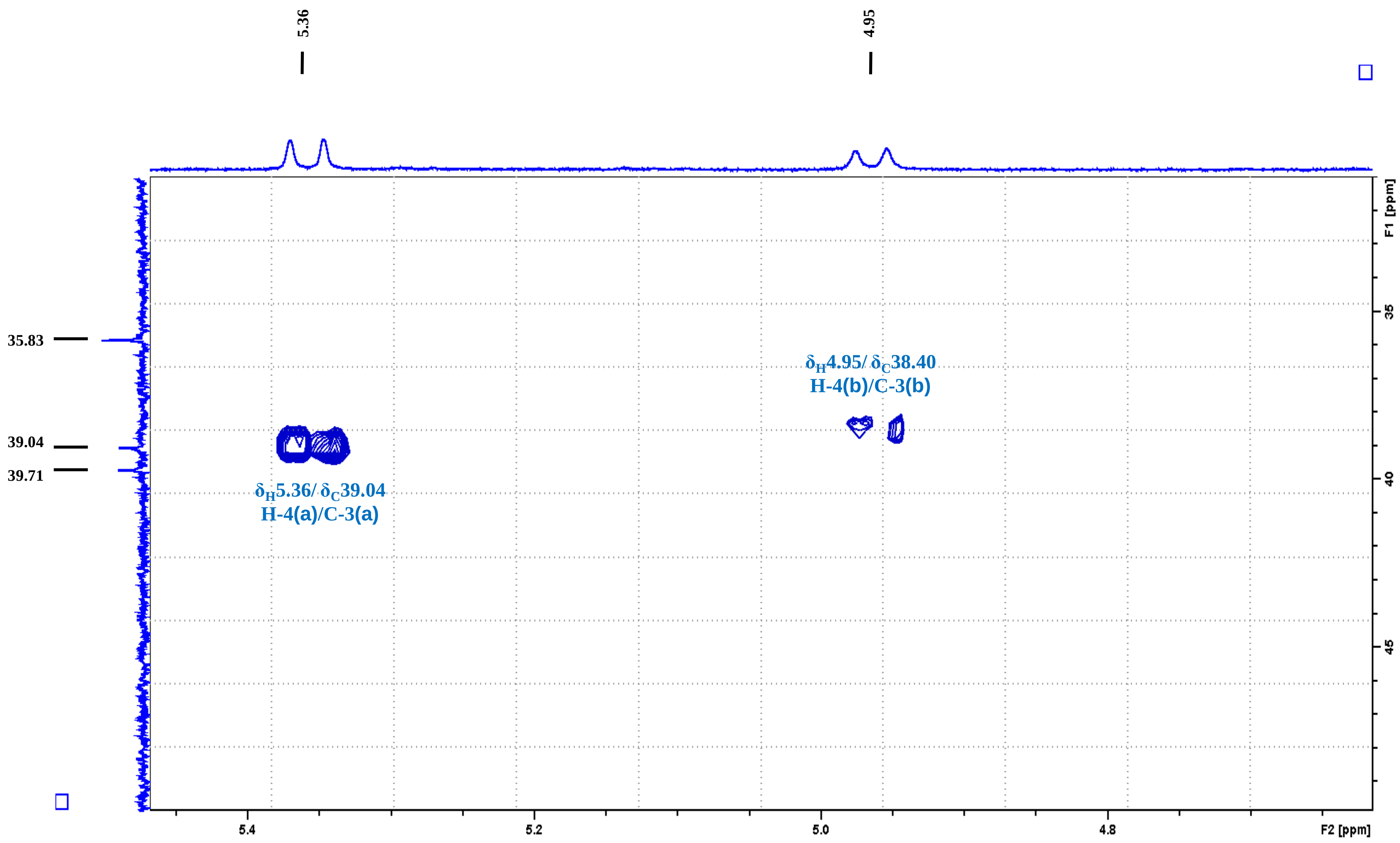


Figure S42. HMBC spectrum of compounds **1a and 1b** (enlarged).

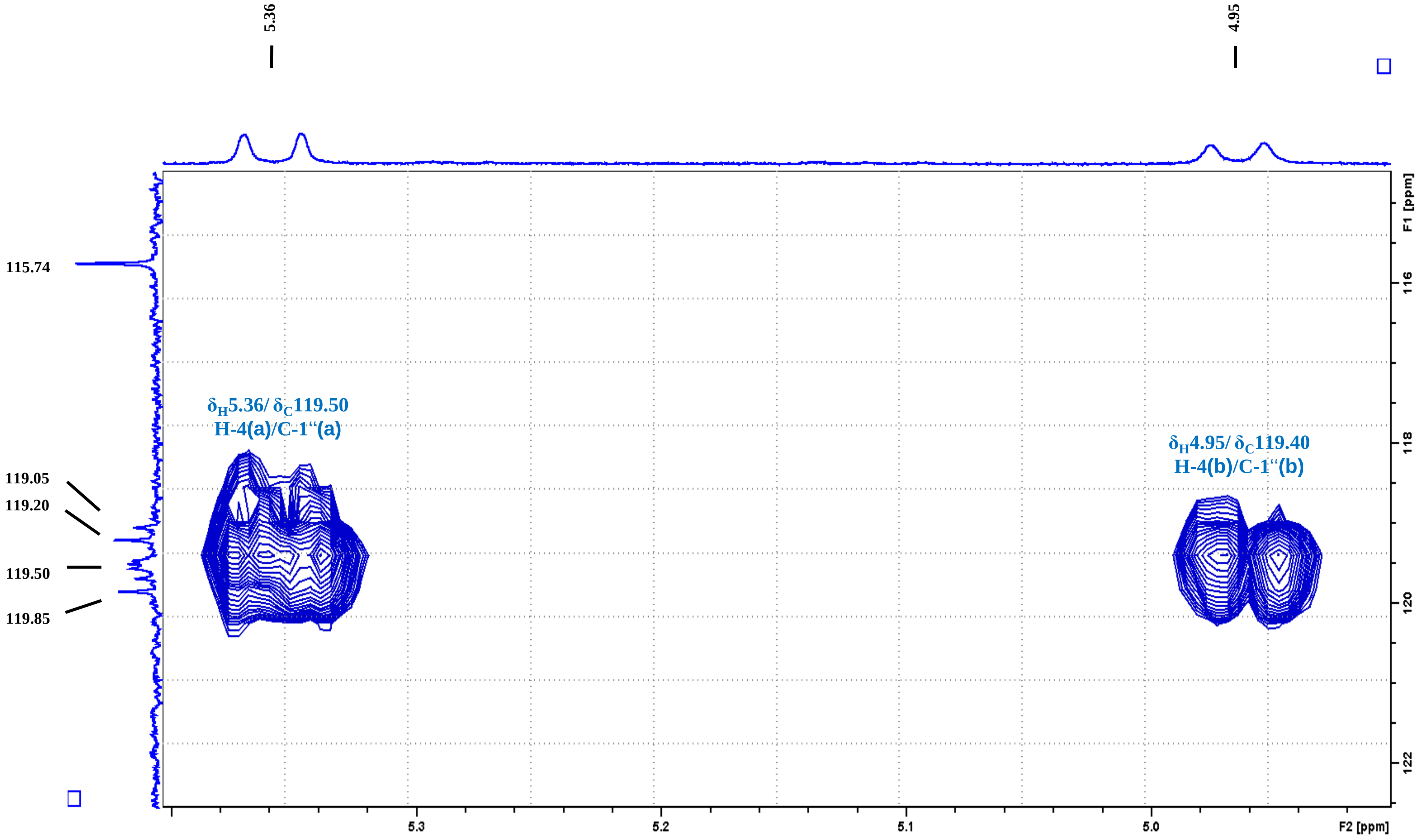


Figure S43. HMBC spectrum of compounds **1a and 1b** (enlarged).

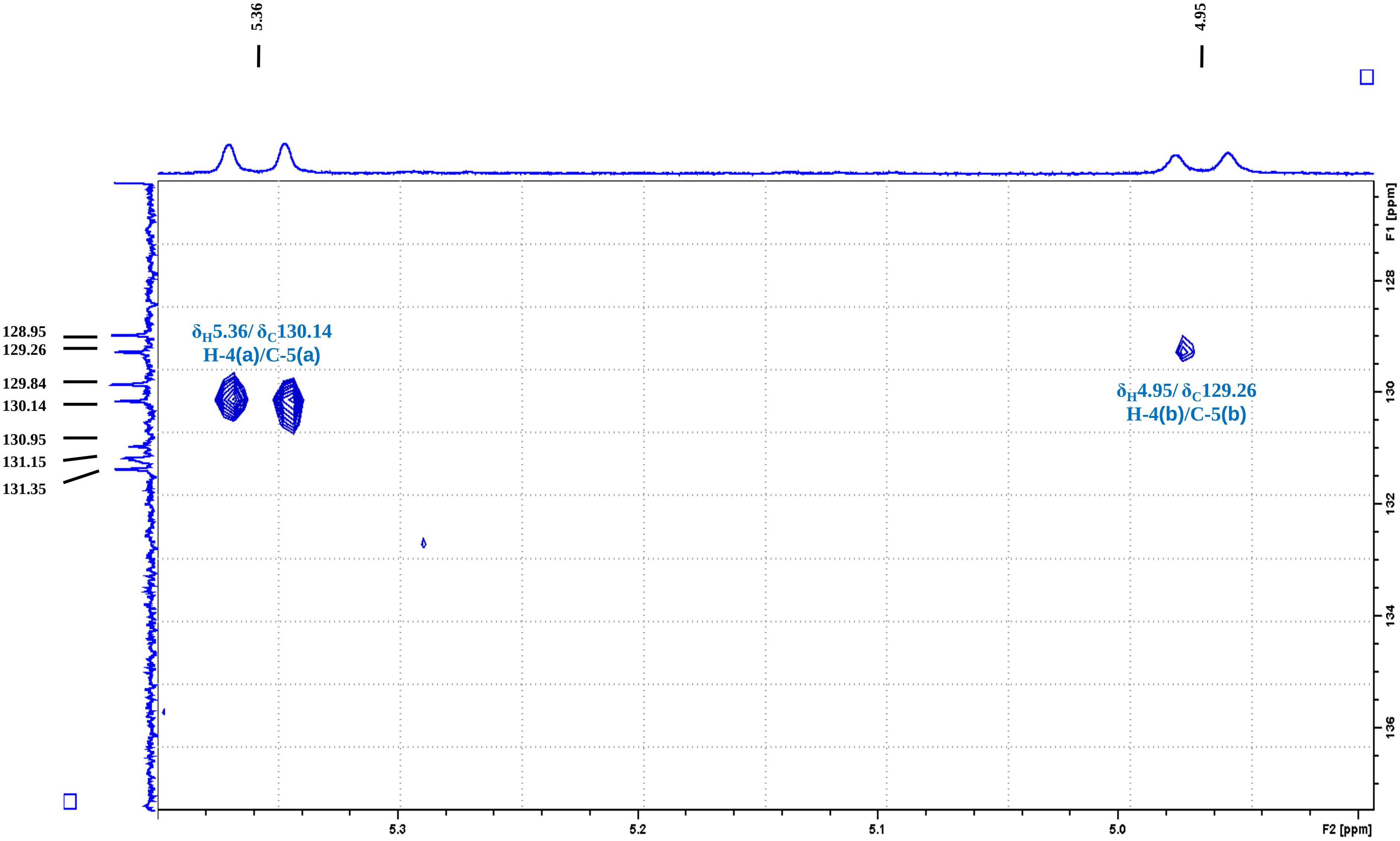


Figure S44. HMBC spectrum of compounds **1a and 1b** (enlarged).

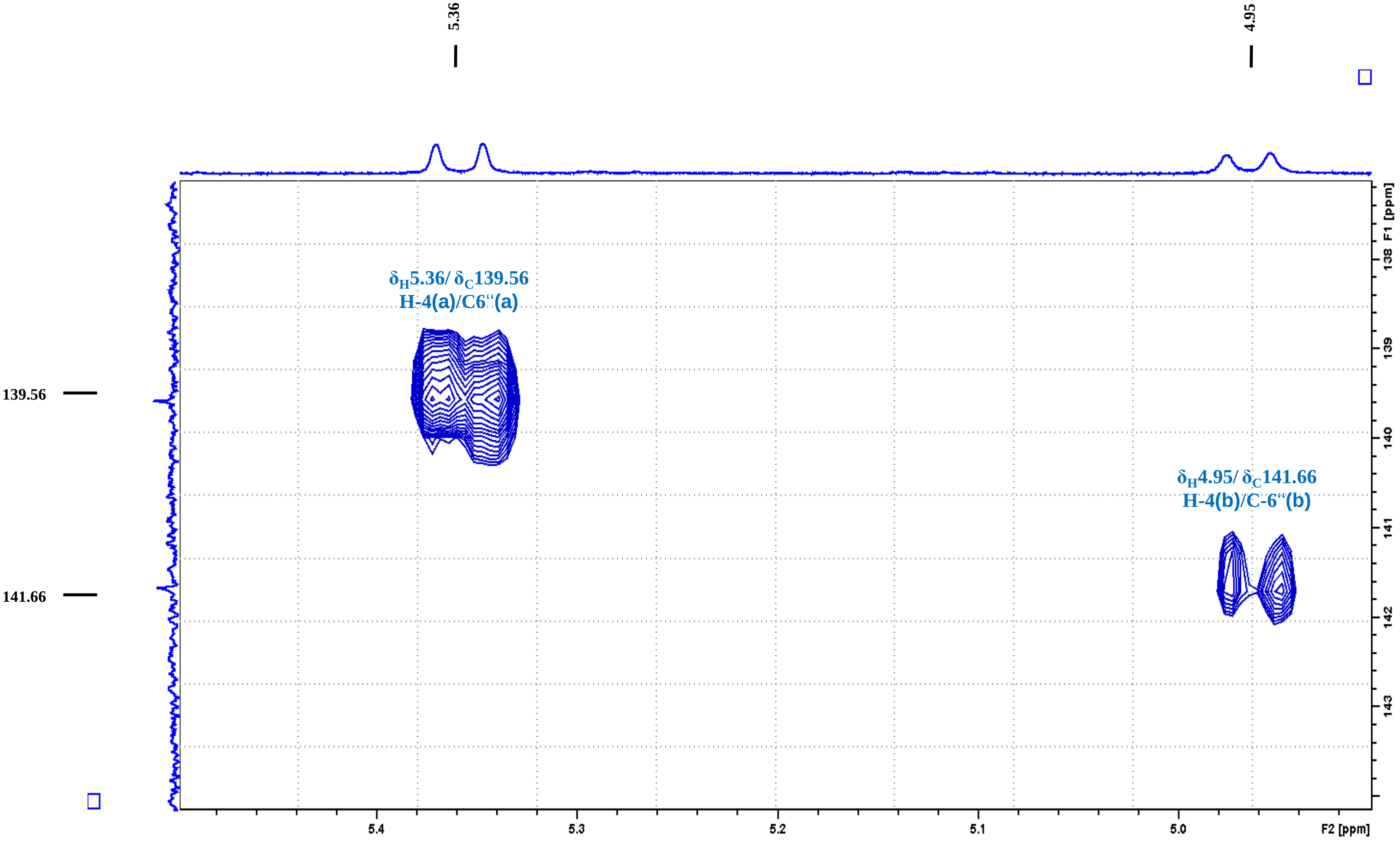


Figure S45. HMBC spectrum of compounds **1a and 1b** (enlarged).

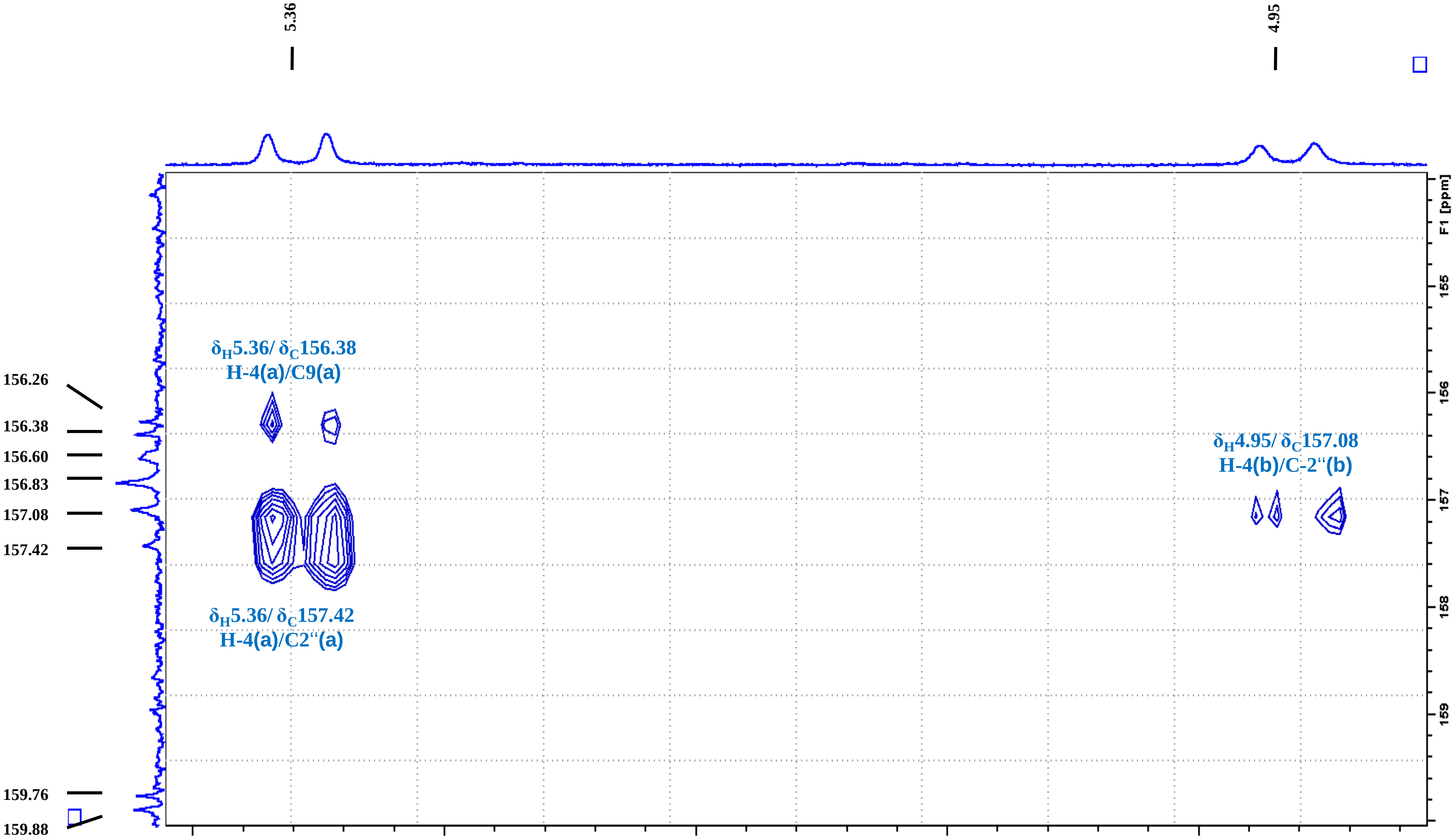


Figure S46. HMBC spectrum of compounds **1a and 1b** (enlarged).

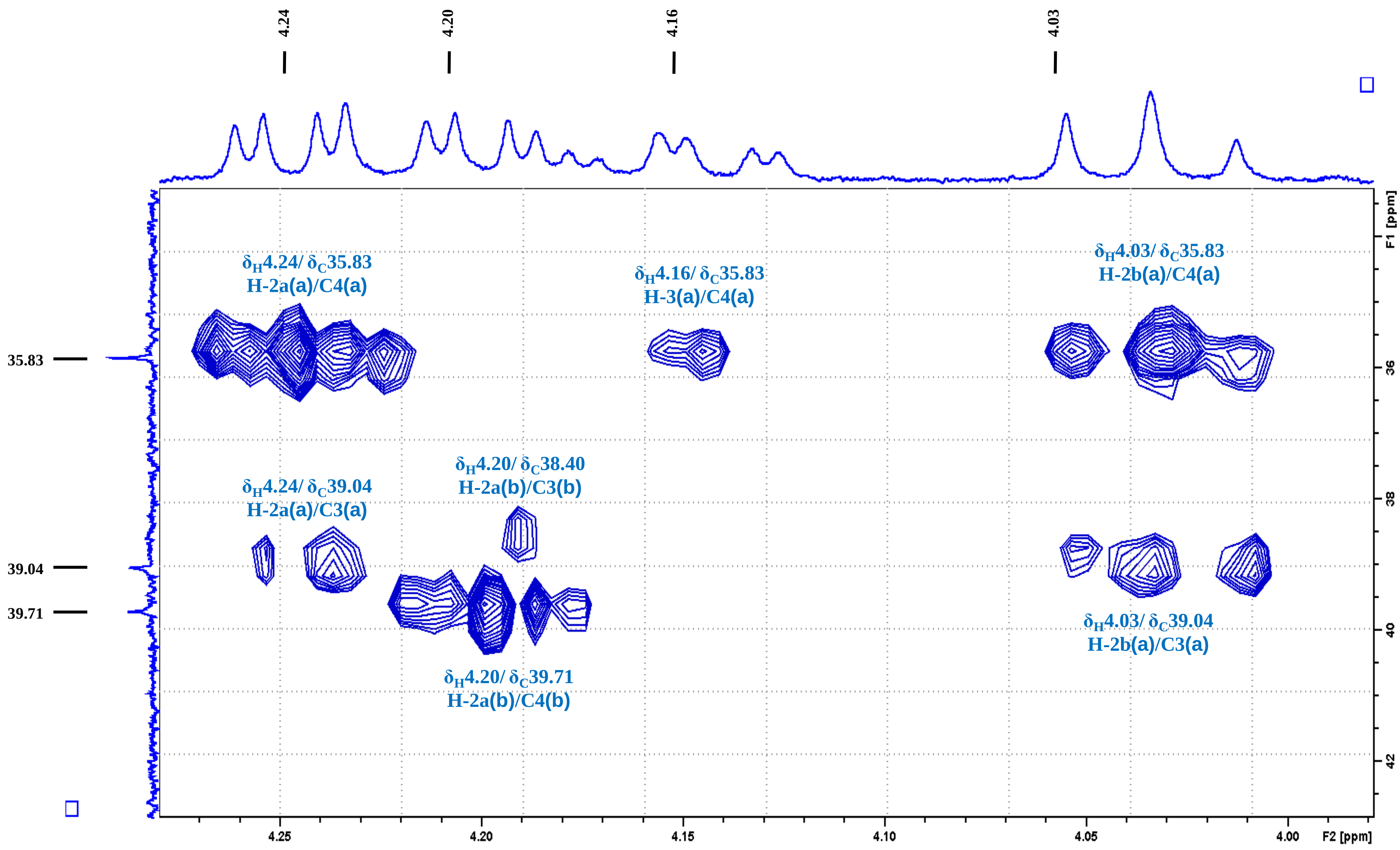


Figure S47. HMBC spectrum of compounds **1a and 1b** (enlarged).

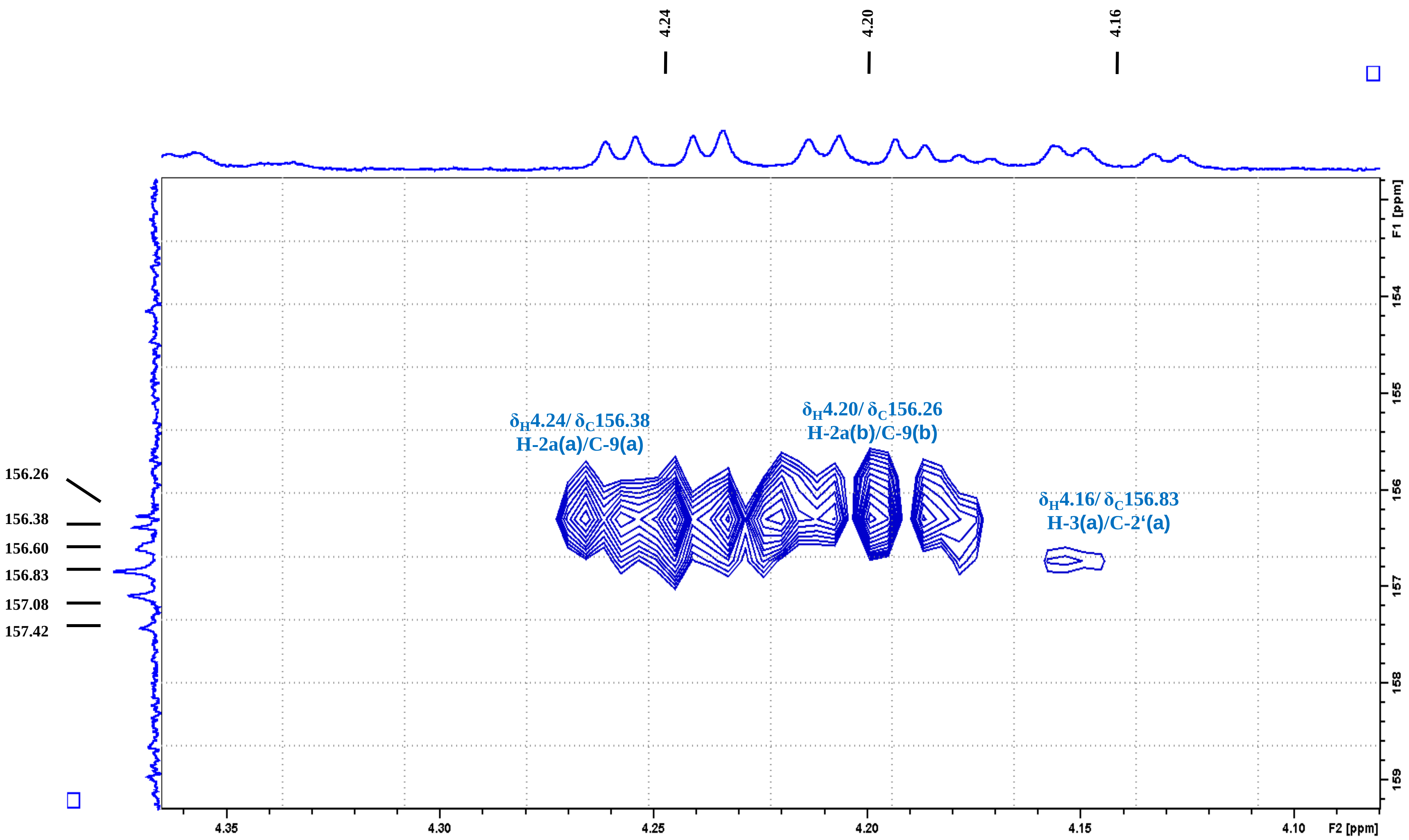


Figure S48. HMBC spectrum of compounds **1a and 1b** (enlarged).

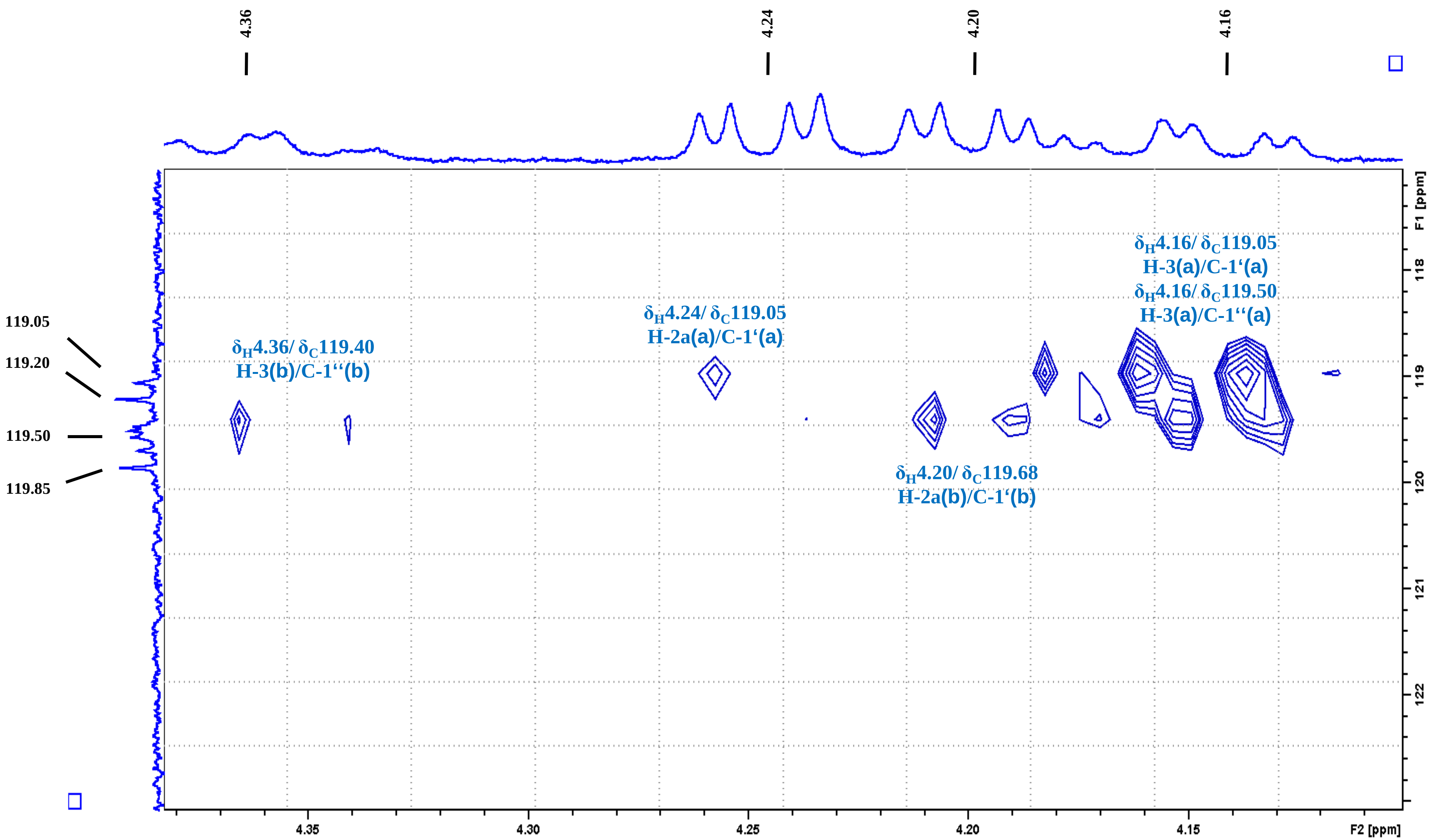


Figure S49. HMBC spectrum of compounds **1a and 1b** (enlarged).

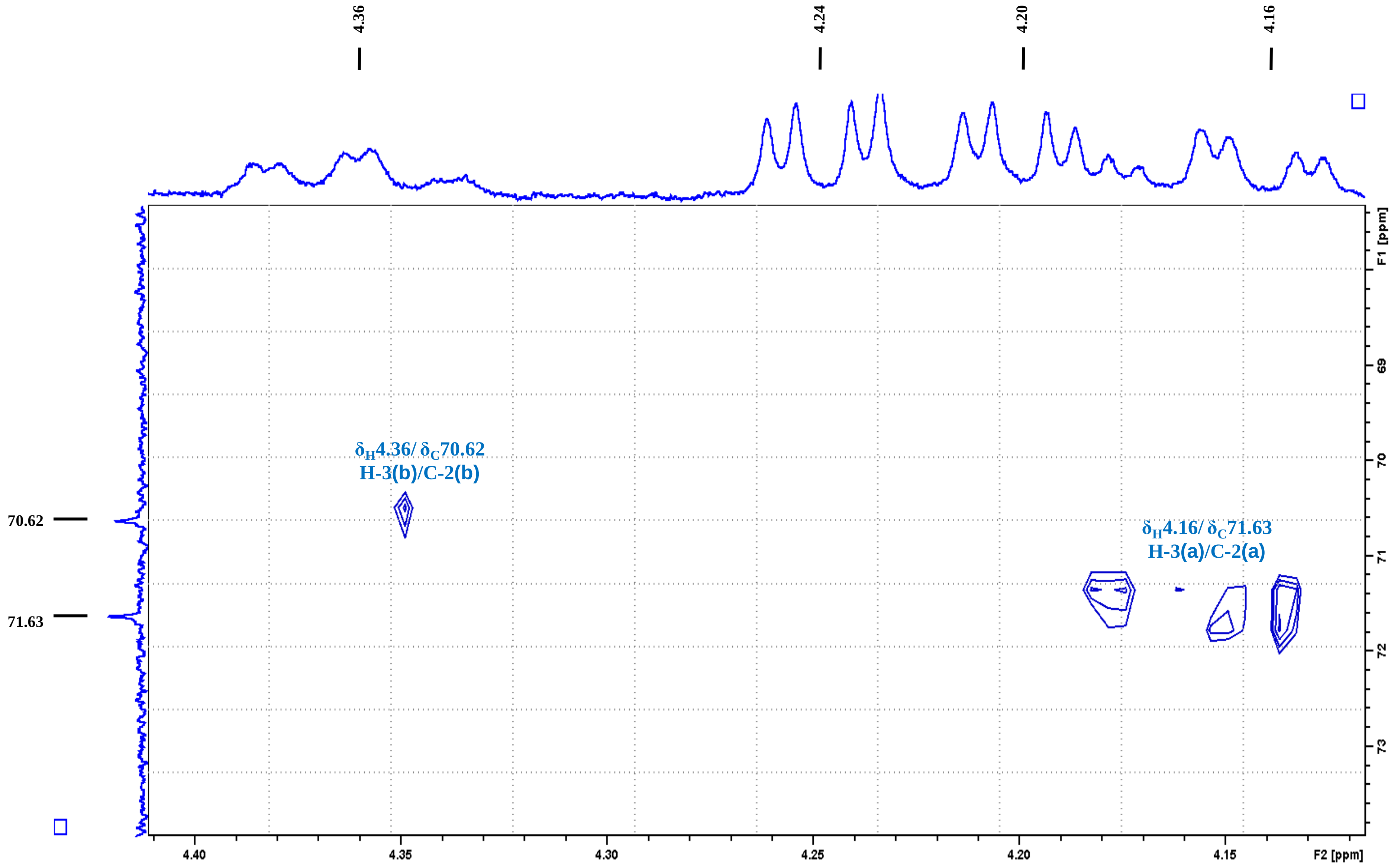


Figure S50. HMBC spectrum of compounds **1a and 1b** (enlarged).

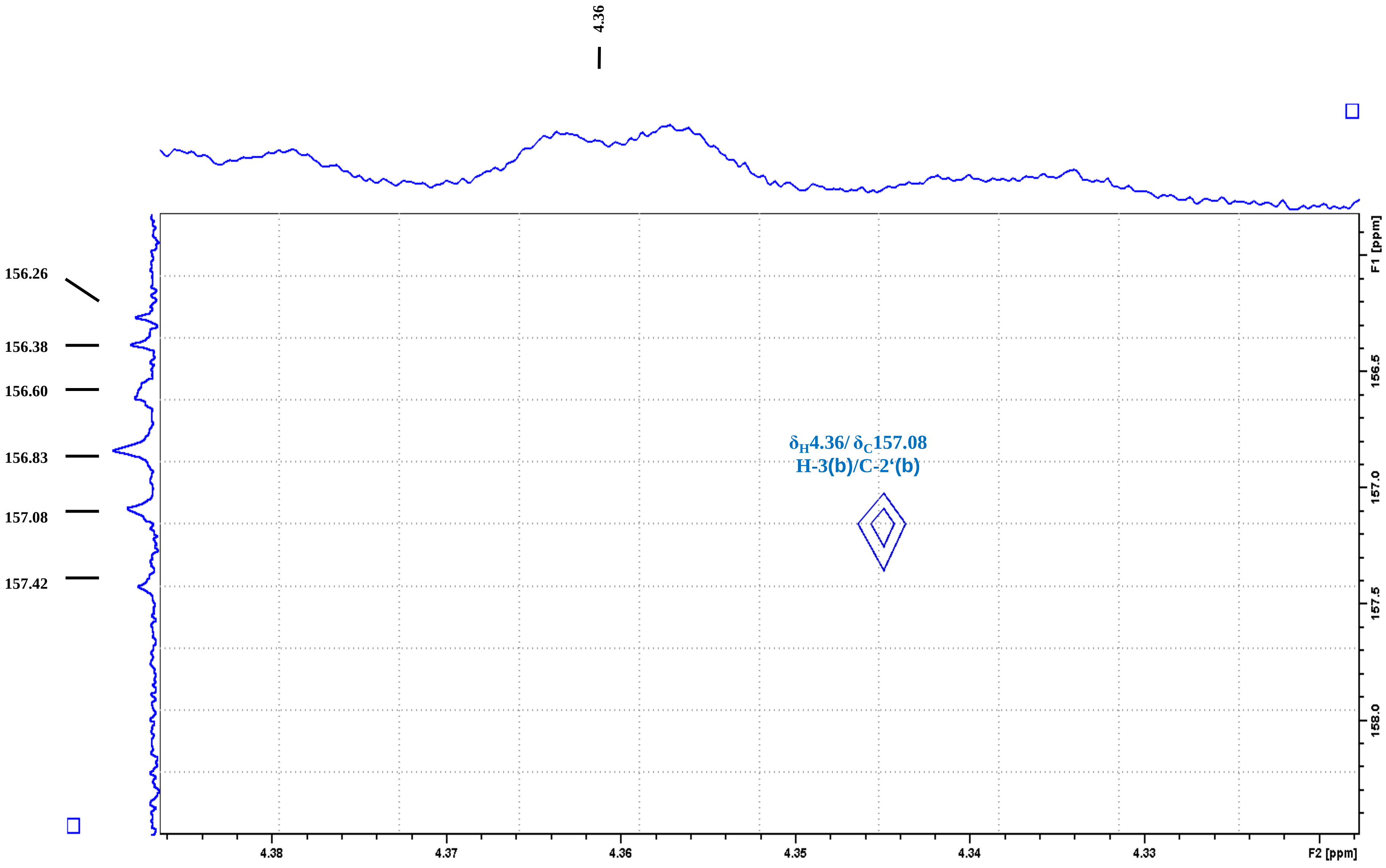


Figure S51. HMBC spectrum of compounds **1a and 1b** (enlarged).

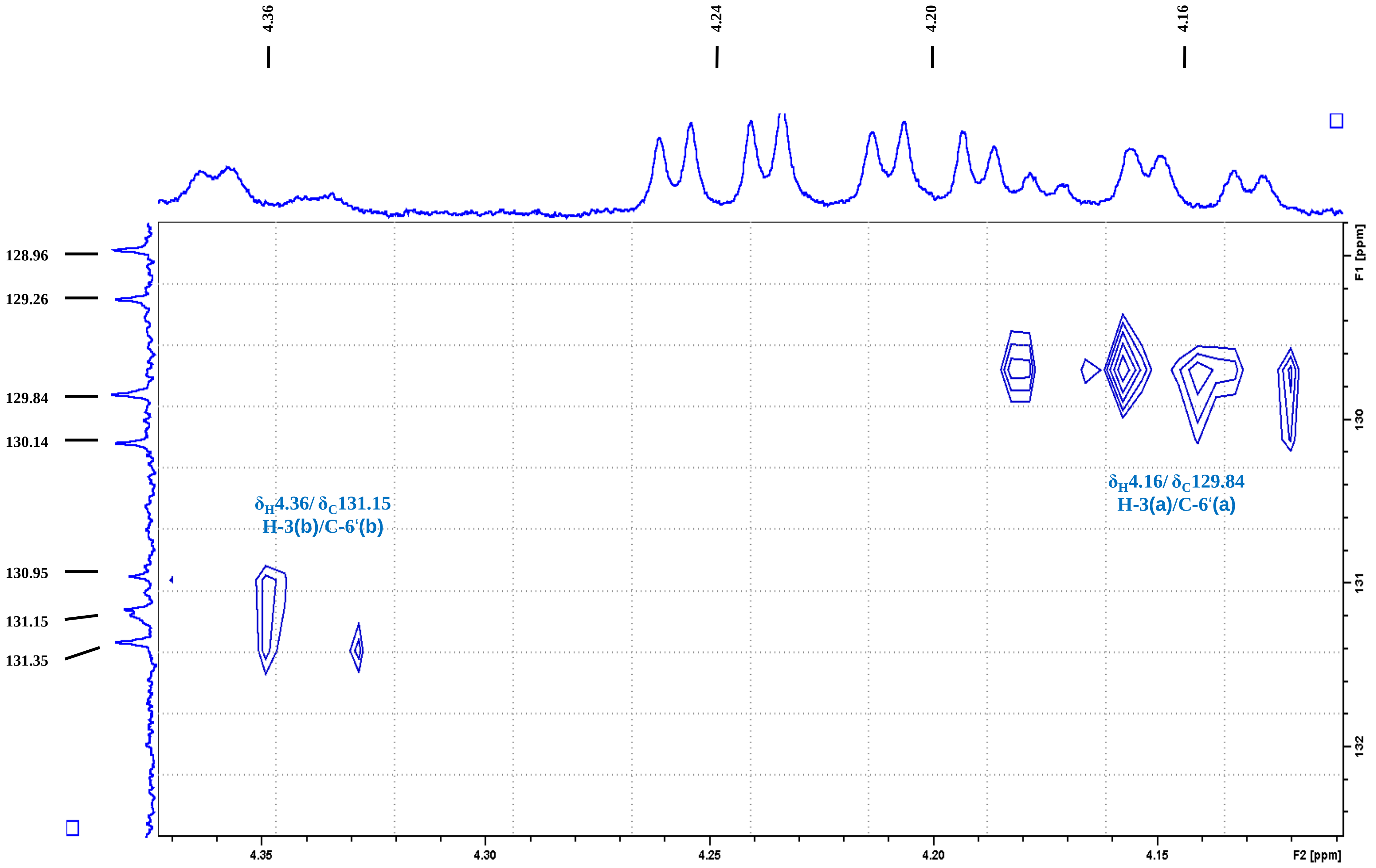


Figure S52. HMBC spectrum of compounds **1a and 1b** (enlarged).

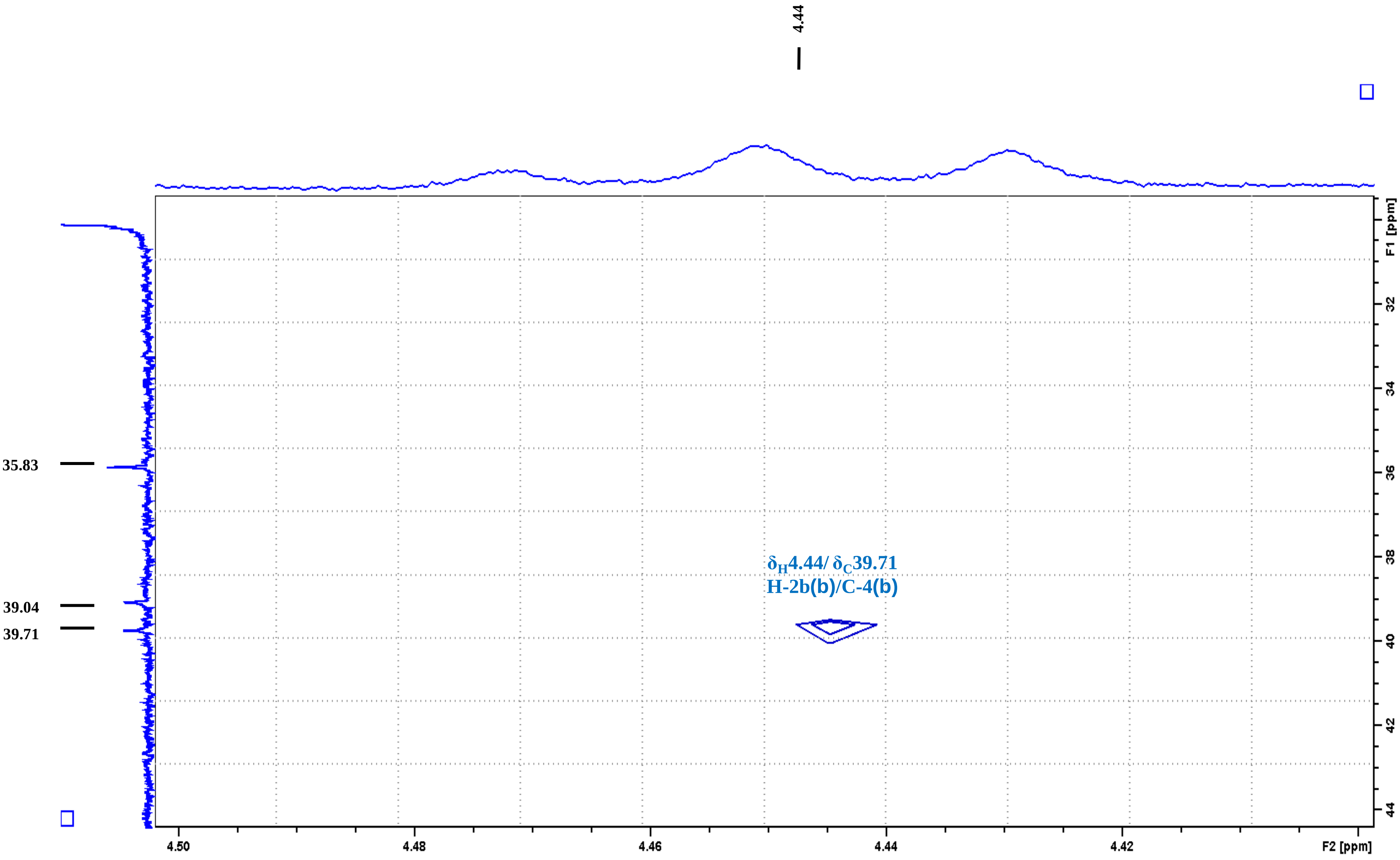


Figure S53. HMBC spectrum of compounds **1a and 1b** (enlarged).

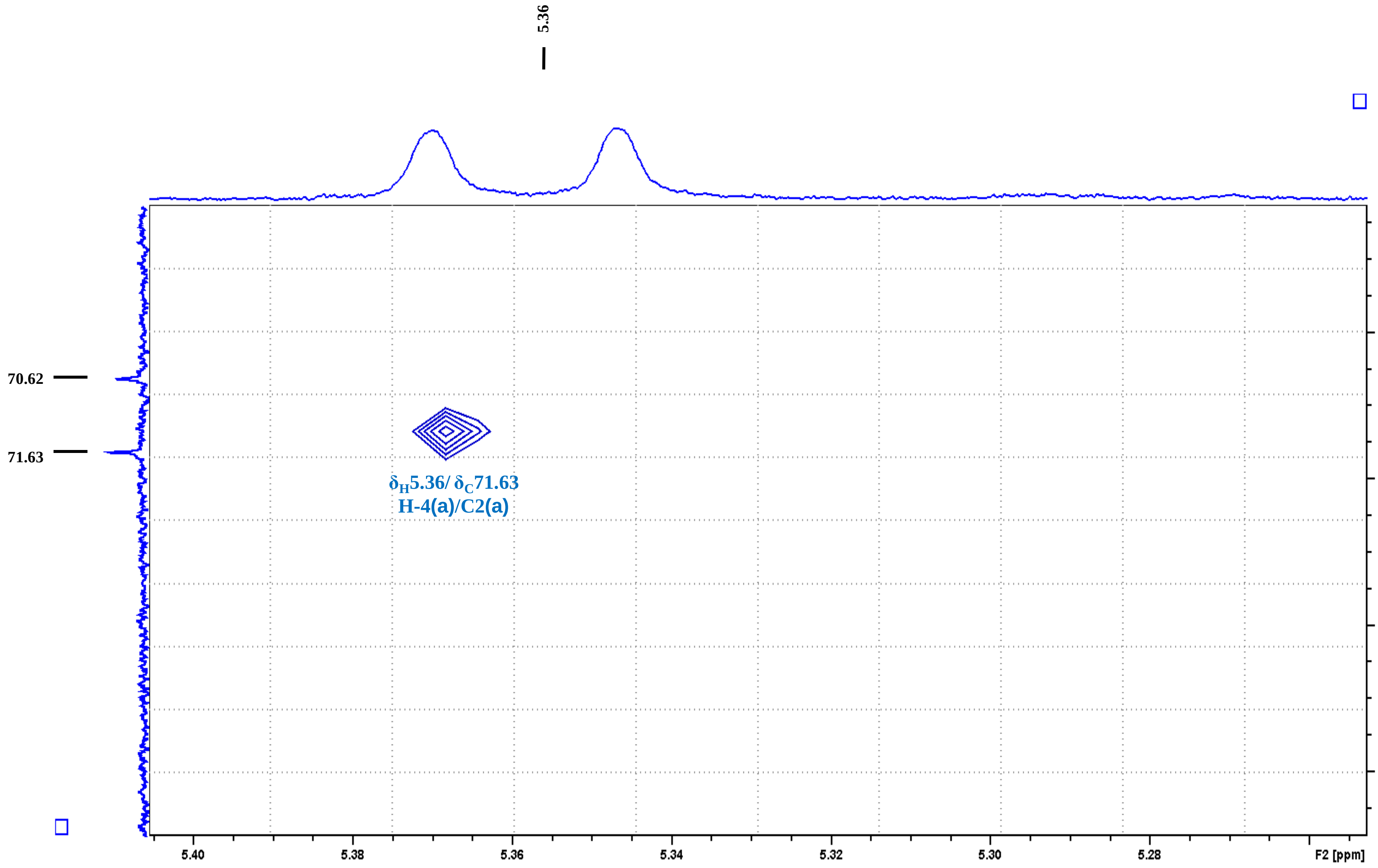


Figure S54. HMBC spectrum of compounds **1a and 1b** (enlarged).

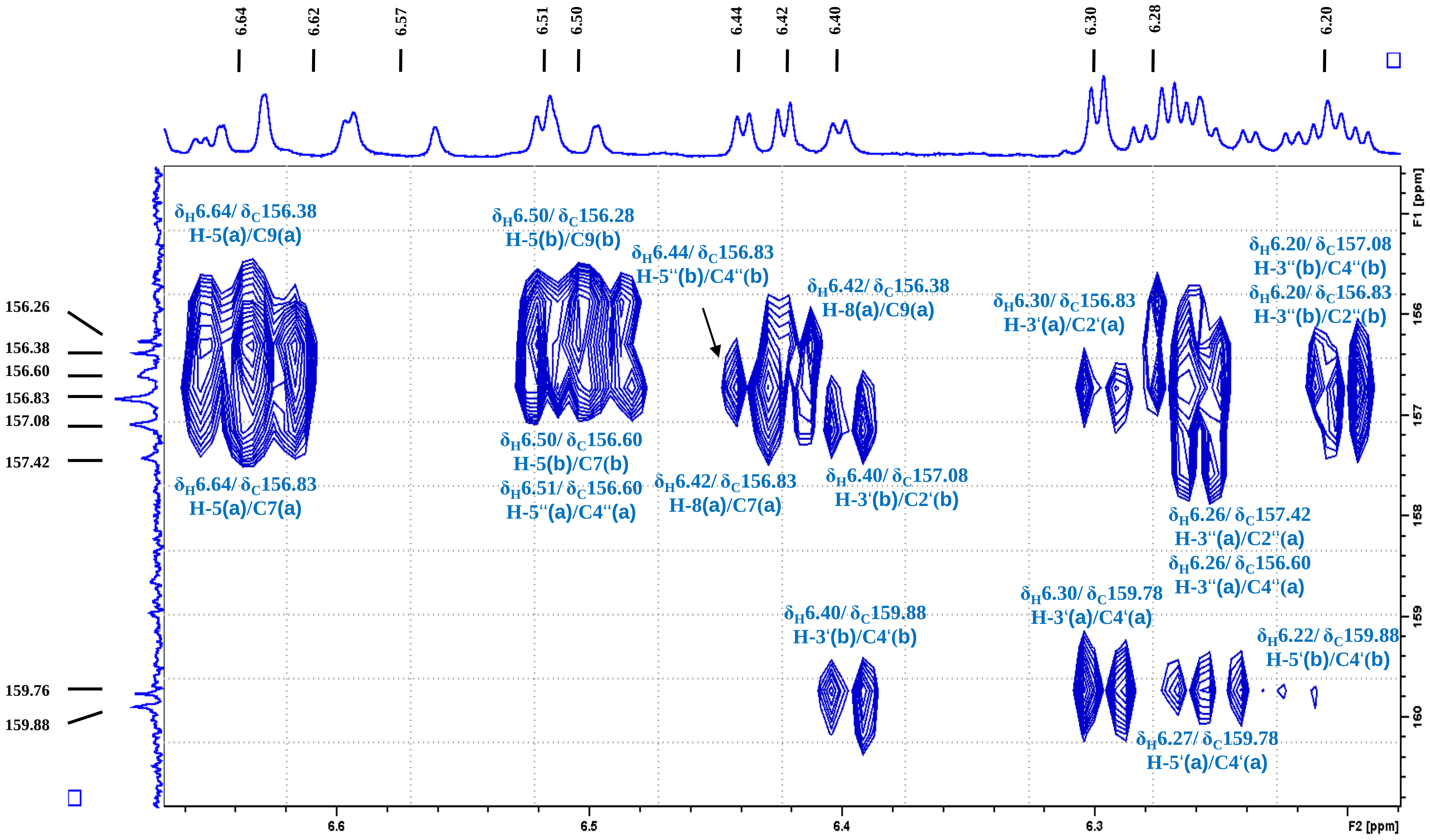


Figure S55. HMBC spectrum of compounds **1a and 1b** (enlarged).

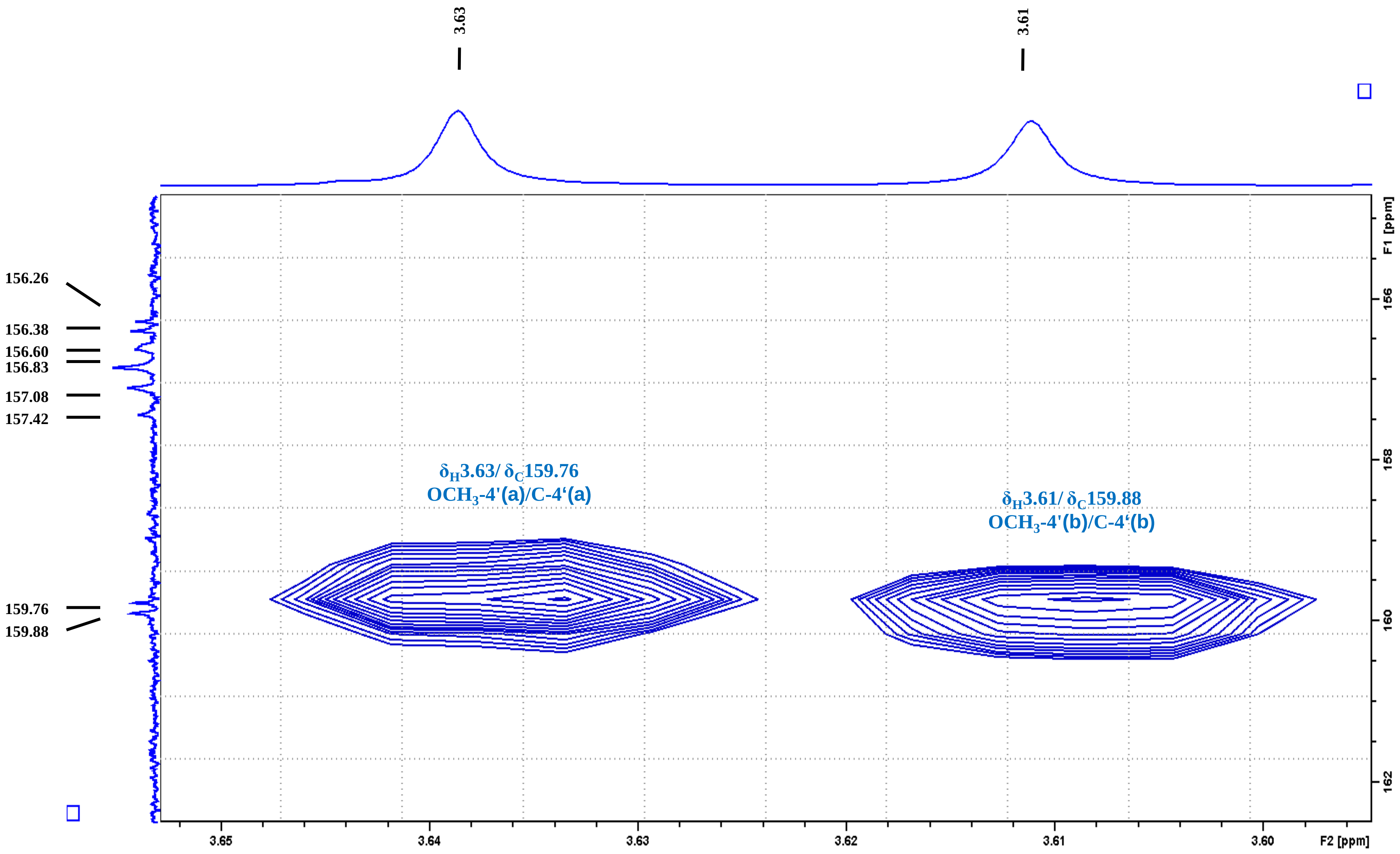


Figure S56. HMBC spectrum of compounds **1a and 1b** (enlarged).

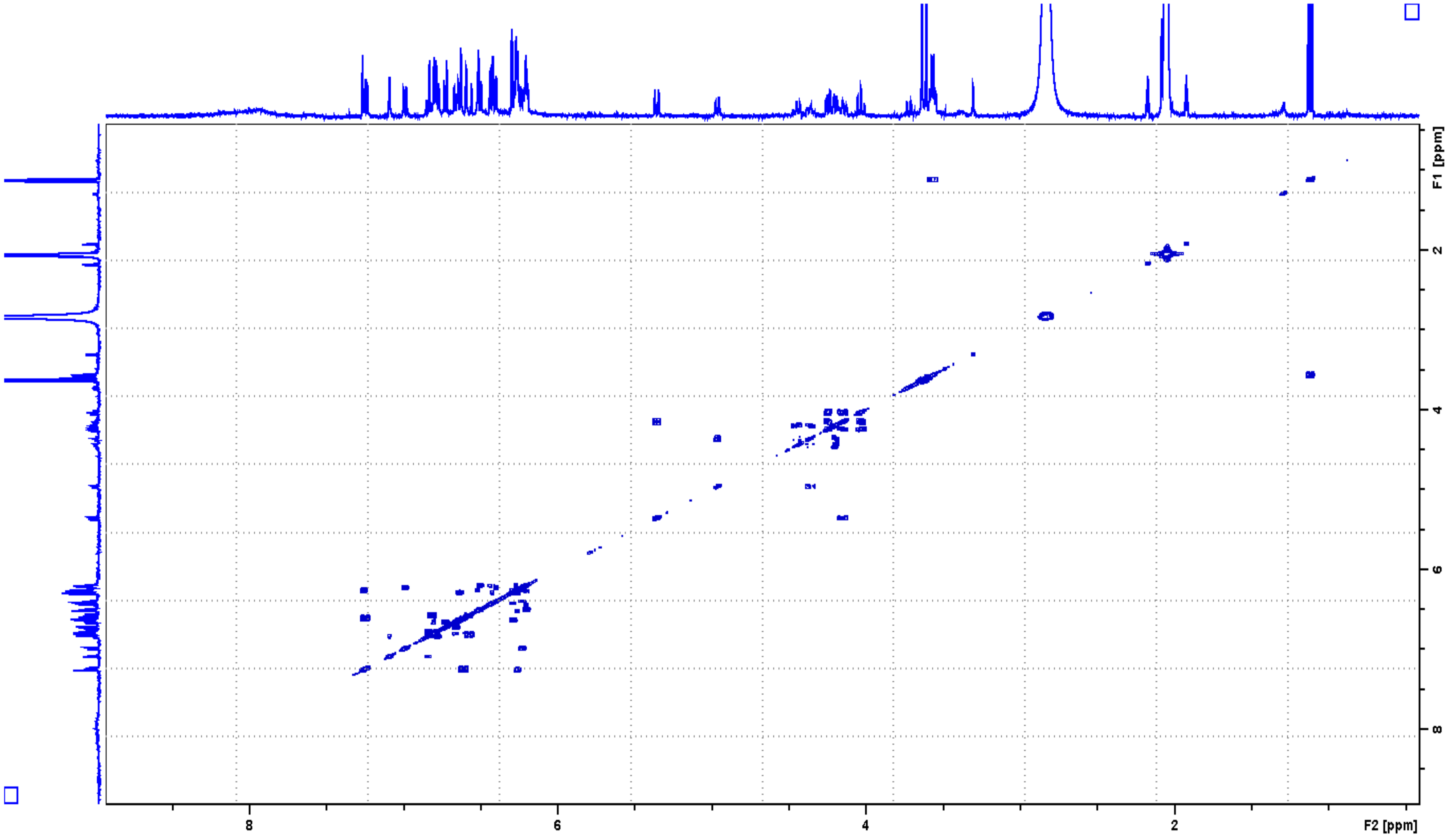


Figure S57. COSY spectrum of compounds **1a and 1b**.

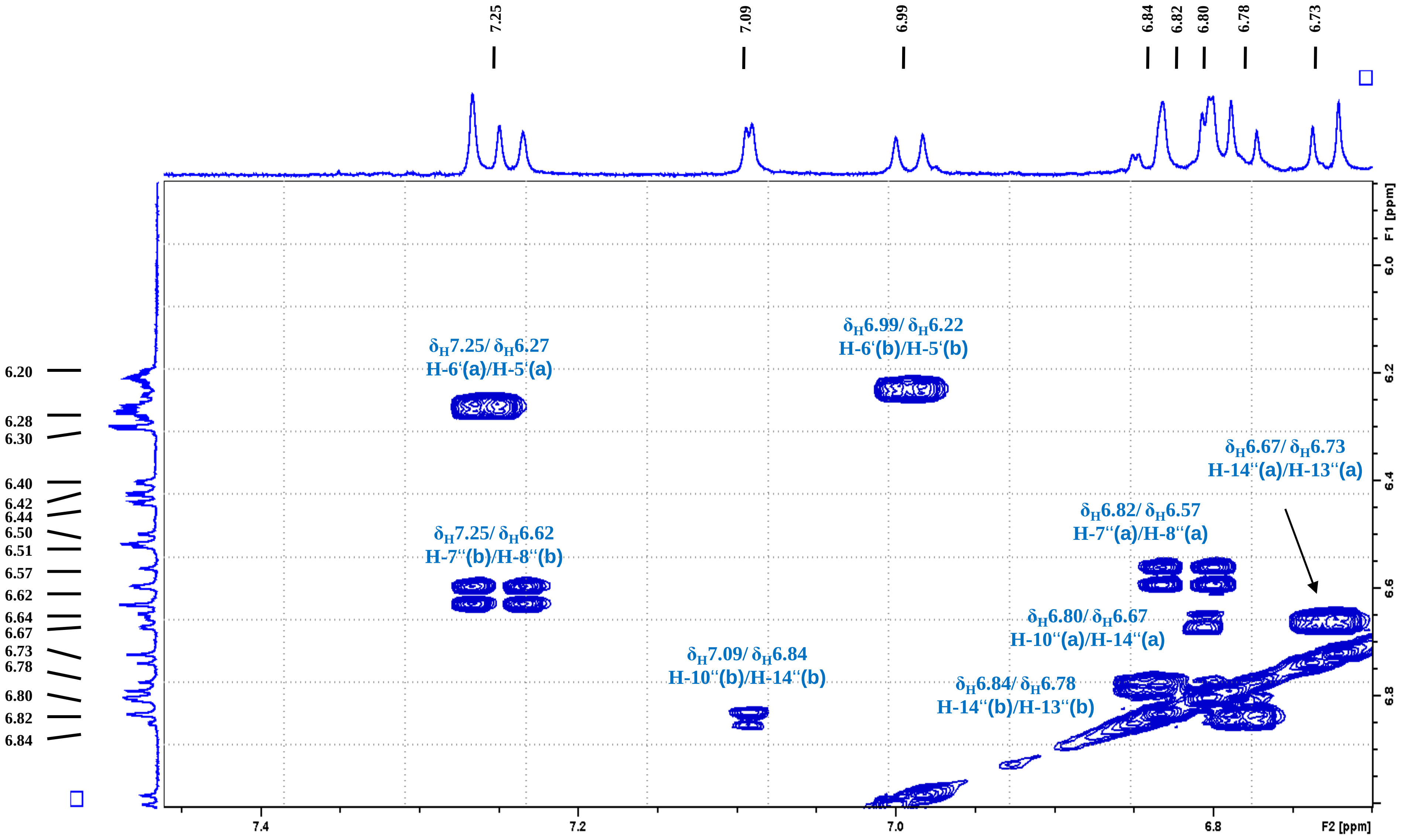


Figure S58. COSY spectrum of compounds **1a and 1b** (enlarged).

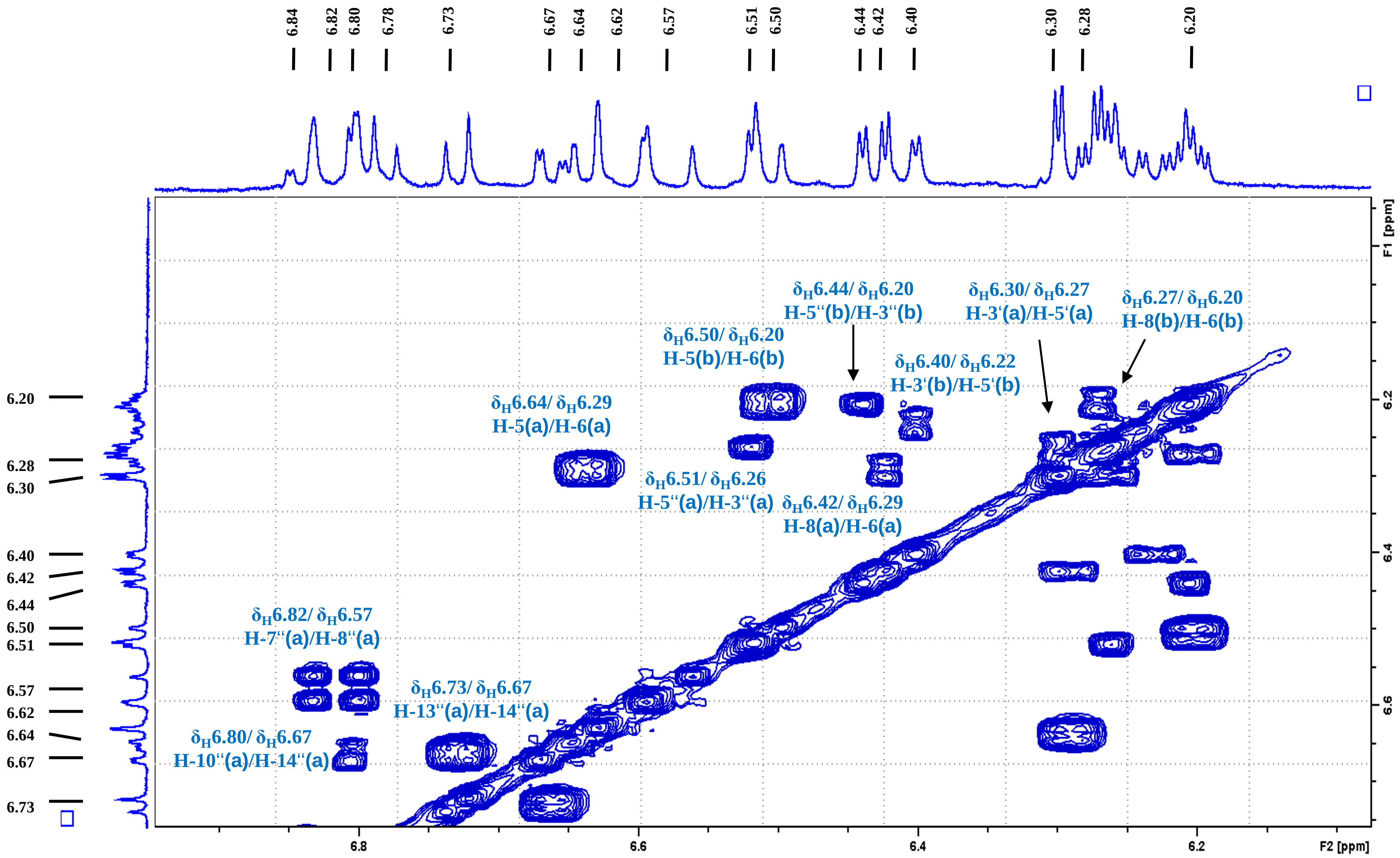


Figure S59. COSY spectrum of compounds **1a and 1b** (enlarged).

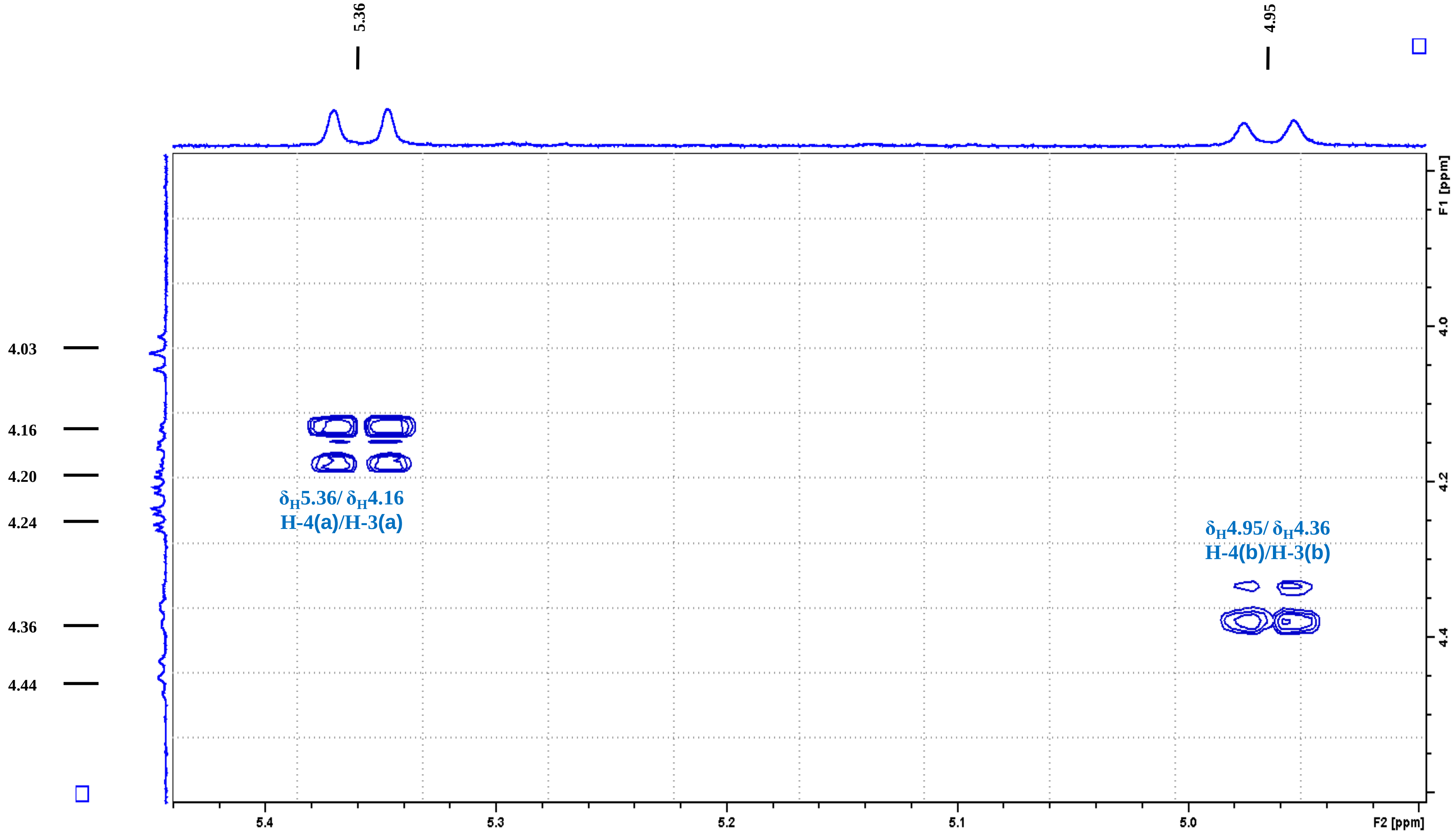


Figure S60. COSY spectrum of compounds **1a and 1b** (enlarged).

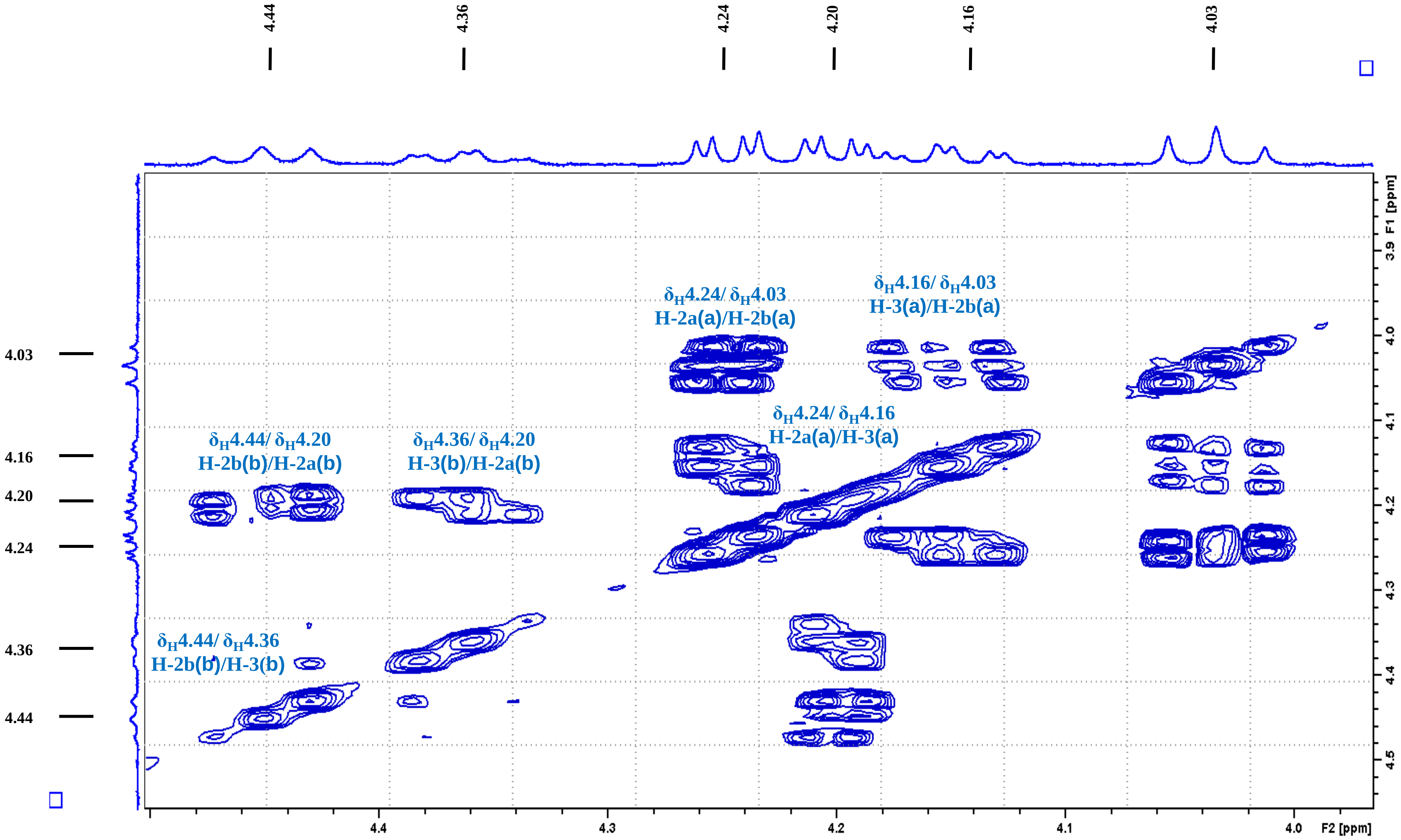


Figure S61. COSY spectrum of compounds **1a and 1b** (enlarged).

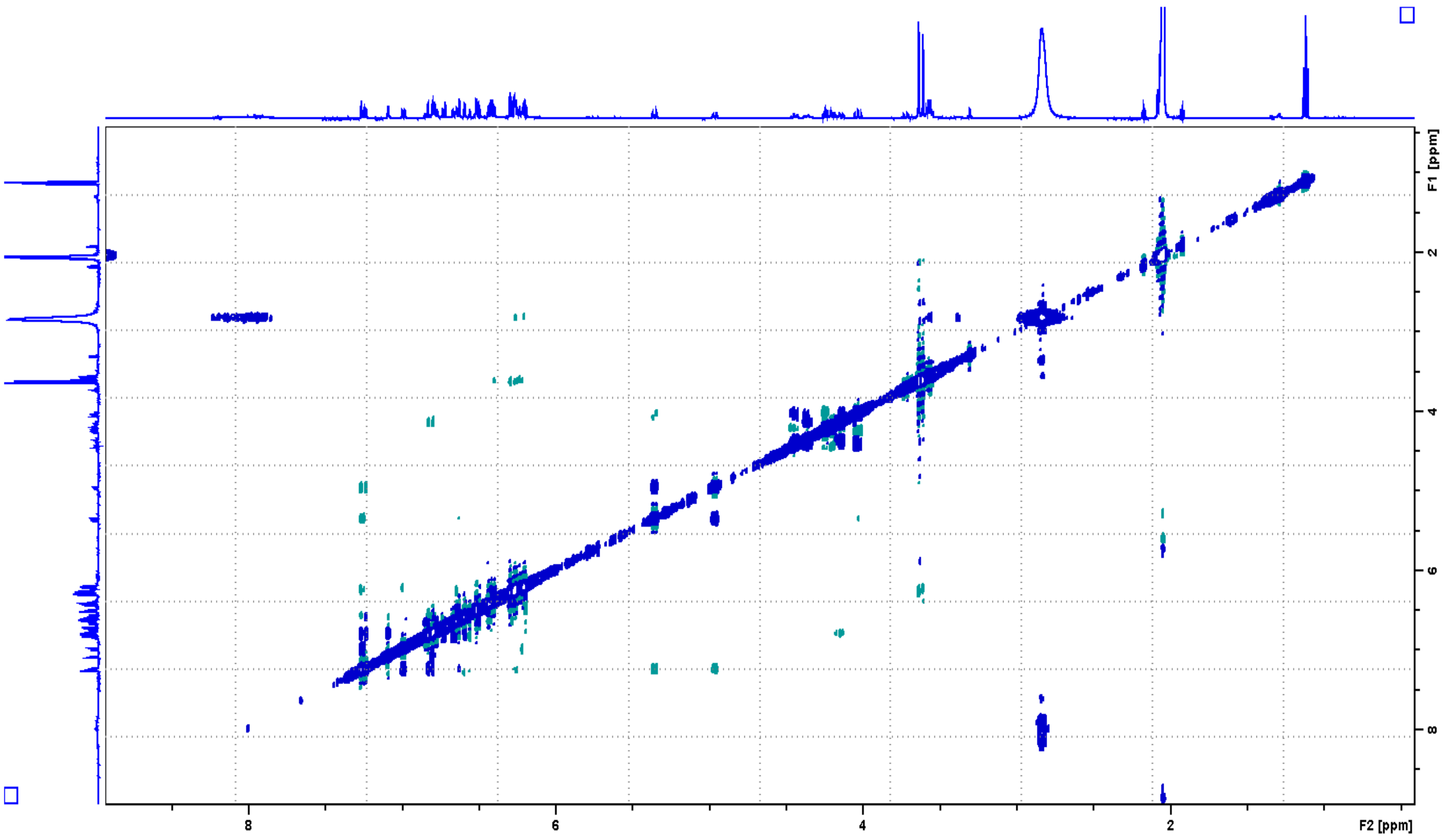


Figure S62. ROESY spectrum of compounds **1a and 1b**.

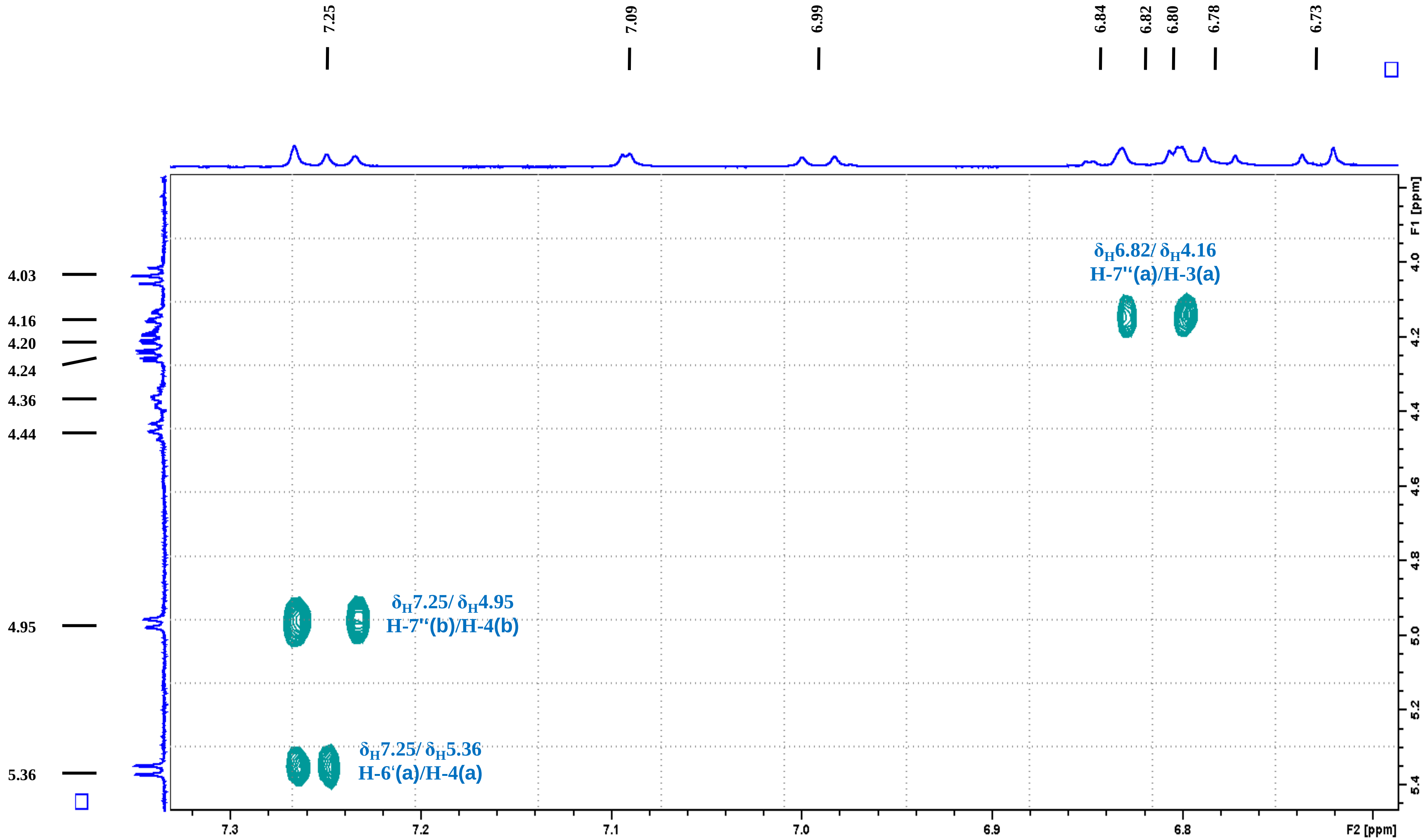


Figure S63. ROESY spectrum of compounds **1a and 1b** (enlarged).

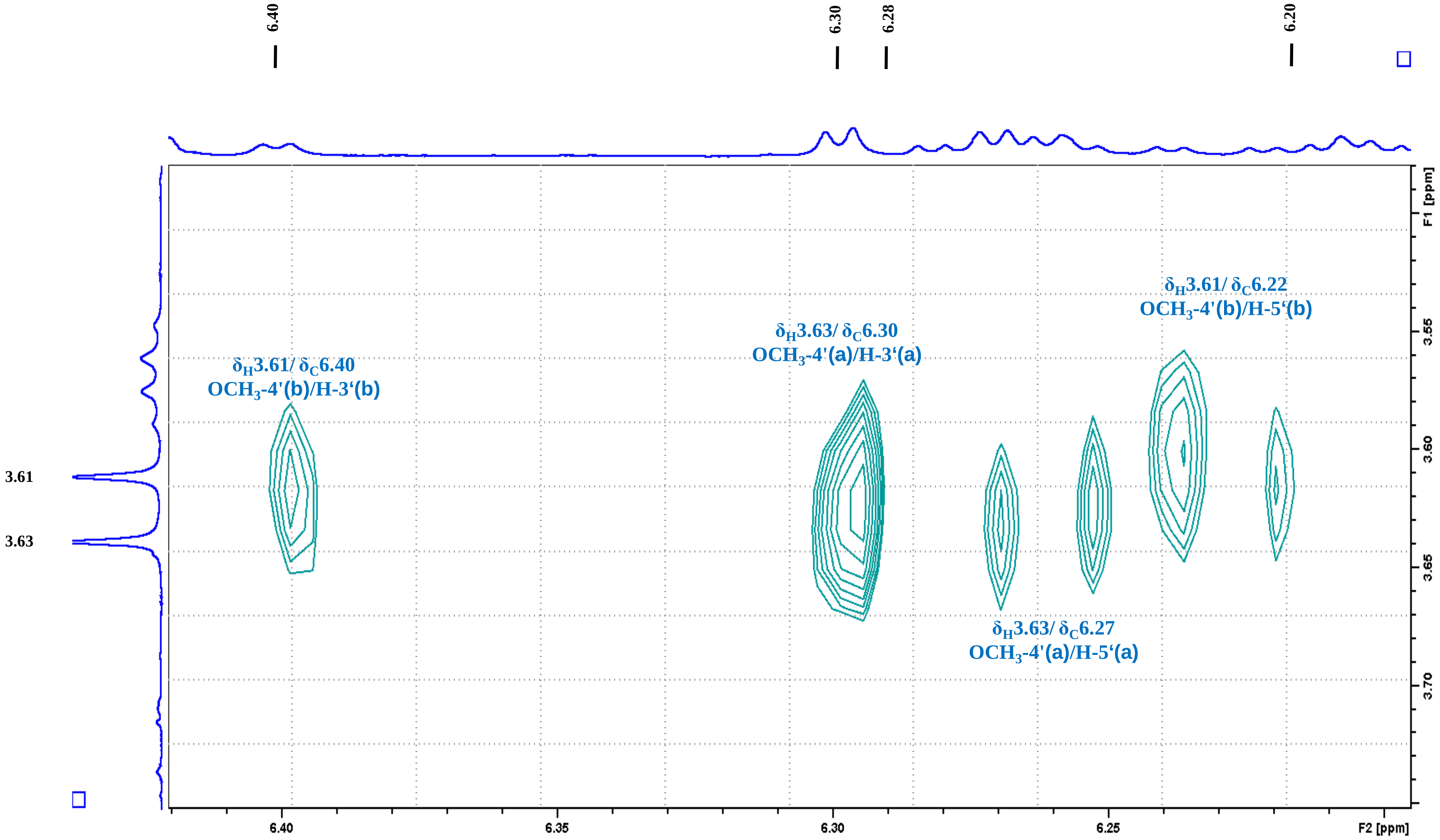


Figure S64. ROESY spectrum of compounds **1a and 1b** (enlarged).

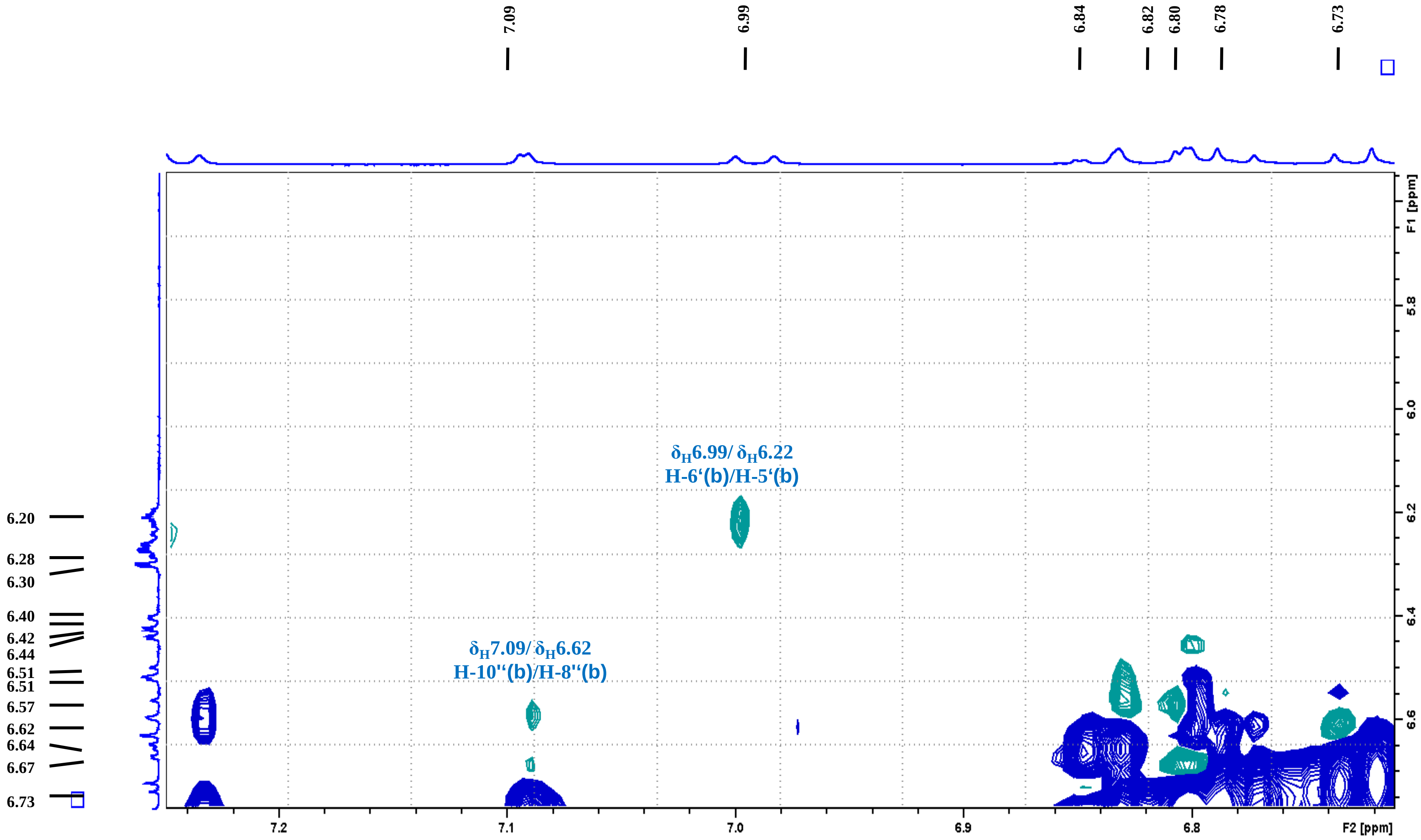


Figure S65. ROESY spectrum of compounds **1a and 1b** (enlarged).

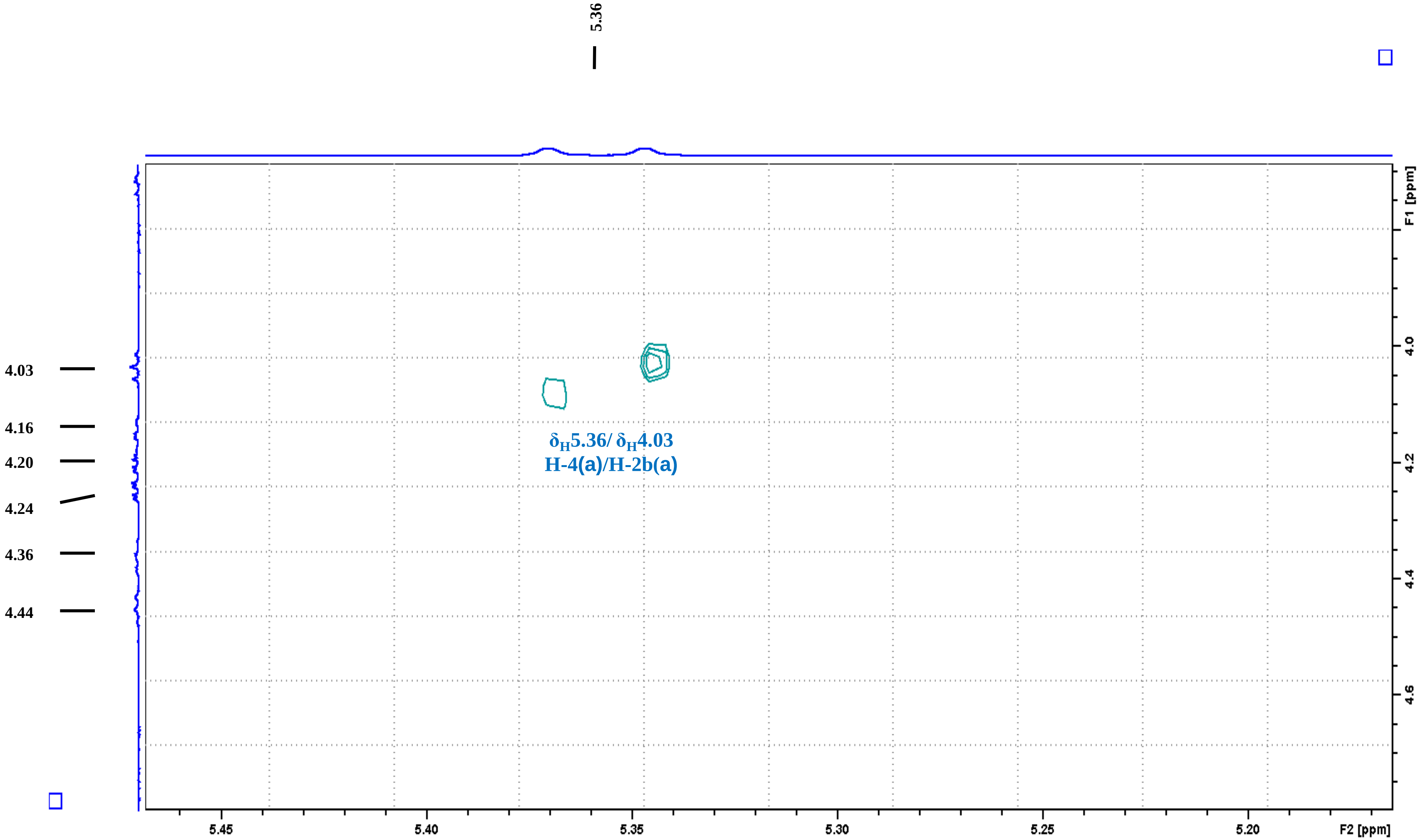


Figure S66. ROESY spectrum of compounds **1a and 1b** (enlarged).

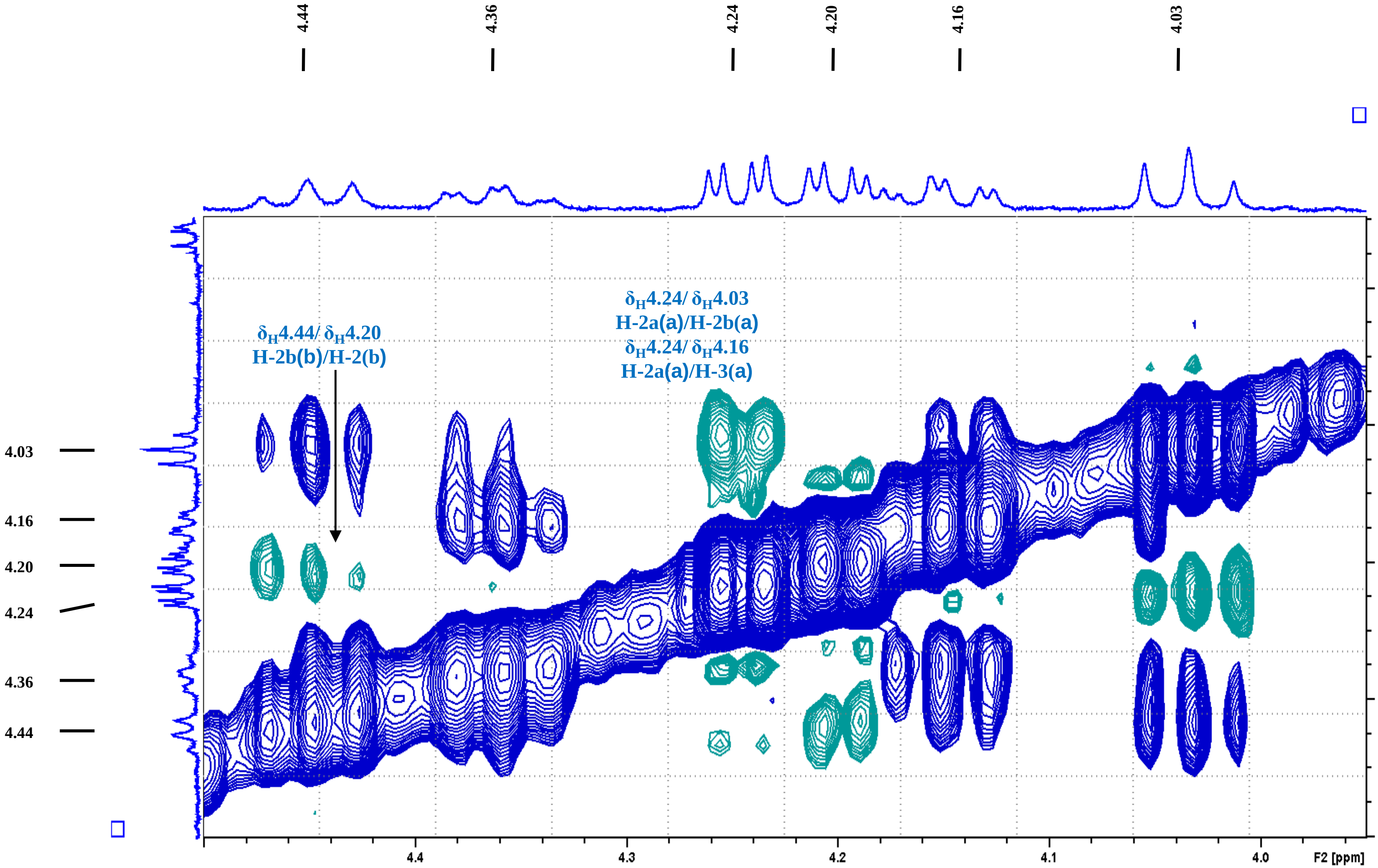


Figure S67. ROESY spectrum of compounds **1a and 1b** (enlarged).

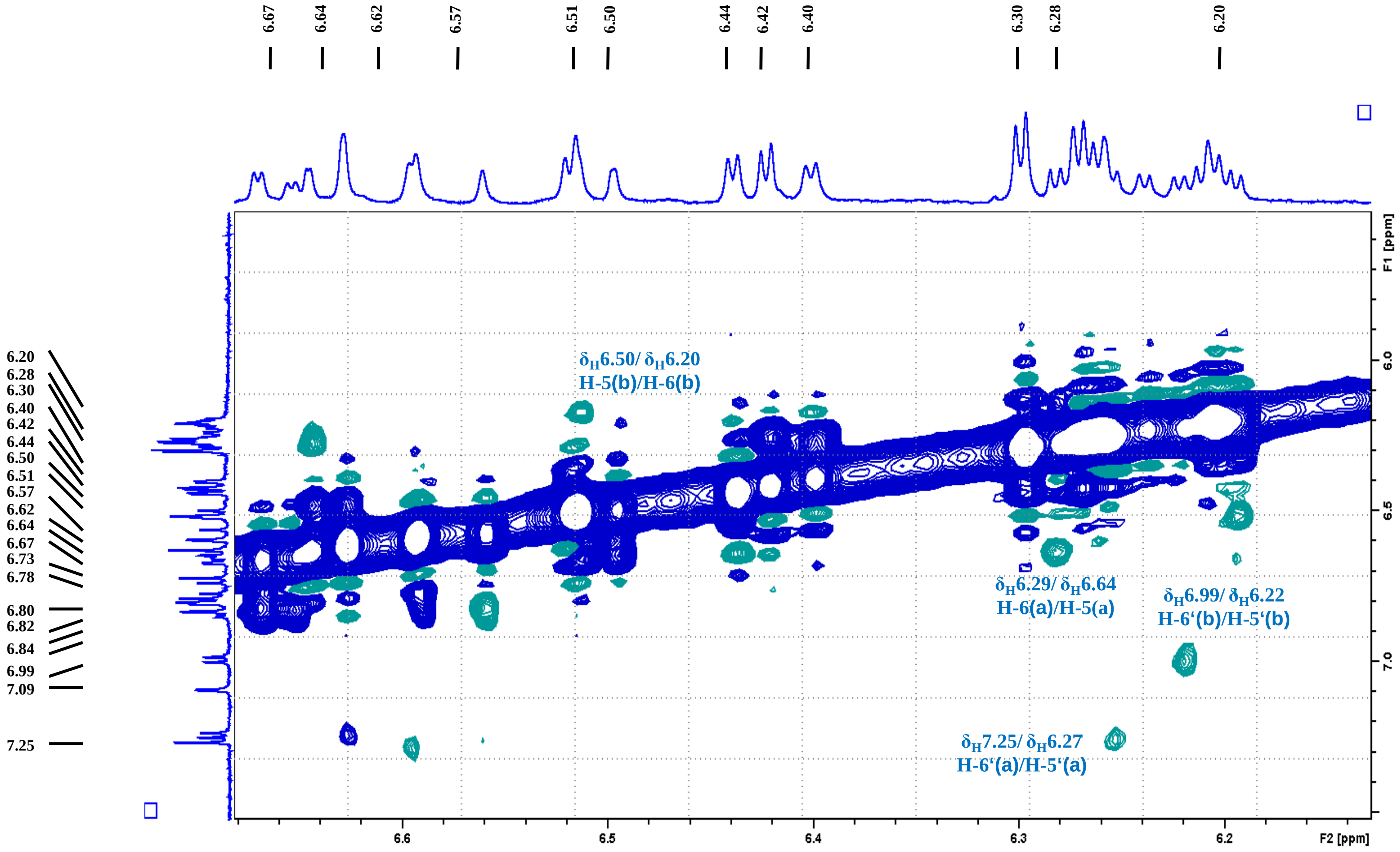


Figure S68. ROESY spectrum of compounds **1a and 1b** (enlarged).

| Compounds | Concentration μg/ml | Ct | -ΔCt | $2^{-\Delta Ct}$ | $log_{10}$ |
|---|---|---|---|---|---|
| Maksar® | 50 | 30,0±3,3 | -13,9±1,7 | 0,0000654 | -4,18 |
| | 5 | 20,6±2,5 | - 4,5±0,5 | 0,0441942 | -1,35 |
| 1 | 50 | 30,0±3,3 | -13,9±1,6 | 0,0000654 | -4,18 |
| | 5 | 20,4±2,4 | -4,3±0,5 | 0,0507657 | -1,29 |
| 2 | 50 | 30,0±3,0 | -13,9±1,6 | 0,0000654 | -4,18 |
| | 5 | 19,2±2,3 | -3,1±0,4 | 0,1166300 | -0,93 |
| 3 | 50 | 30,0±3,0 | -13,9±1,7 | 0,0000654 | -4,18 |
| | 5 | 19,7±2,4 | -3,6±0,4 | 0,0824692 | -1,08 |
| 4 | 50 | 30,0±3,1 | -13,9±1,6 | 0,0000654 | -4,18 |
| | 5 | 20,5±2,4 | -4,4±0,5 | 0,0473661 | -1,32 |
| 5 | 50 | 27,6±3,3 | -11,5±1,4 | 0,0003452 | -3,46 |
| | 5 | 18,7±2,2 | -2,6±0,3 | 0,1649384 | -0,78 |
| 6 | 50 | 23,6±2,6 | -7,5±0,9 | 0,0055242 | -2,26 |
| | 5 | 19,3±2,1 | -3,2±0,4 | 0,1088188 | -0, 96 |
| Virus control (DMSO) | | 16,1±1,9 | 0 | 1,0 | |
| Cell control (DMSO) | | 31,0±3,5 | | | |

Table S69. Anti-HSV-1 activity of polyphenolic compounds from *M. amurensis* (RT-PCR).
Ct - cycle threshold in real-time PCR; ΔCt - the difference between Ct value for infected cells treated with polyphenolic compounds and Ct value for the virus control. Values are presented as means ± standard deviations of three independent experiments.

S70. *Quantum-chemical modeling*

The quantum-chemical calculations for **1** in $CH_3OH$ were performed using density functional theory (DFT) with B3LYP exchange-correlation functional and the polarization continuum model (PCM), implemented in the Gaussian 16 package of programs [35]. The conformational analysis was performed with B3LYP/6-311G(d)_PCM level of theory. The statistical weights ($g_{im}$) of different conformations were obtained using equation SE1:

$$g_{im} = e^{-\Delta G_{im}/RT} \Big/ \sum_i e^{-\Delta G_{im}/RT} \qquad \text{(SE1)}$$

where the summation was done over stable conformations of **1** with $\Delta G_{im} \leq 5$ kcal/mol; (the subscript "m" denotes conformation with minimal *G*.

The excitation energies and the rotatory strengths were calculated using time-dependent density functional theory (TDDFT). Each individual transition from electronic ground state to the *i*-th calculated excited electronic state ($1 \leq i \leq 50$) was simulated as a Gauss-type function. The same values for the bandwidths at 1/*e* peak heights were used.

The total theoretical UV and ECD spectra were obtained after statistical averaging over all selected conformations (equations SE2 and SE3):

$$Absorbance_{calc}(\lambda) = \sum_i g_i \cdot Absorbance_{i,calc}(\lambda) \qquad \text{(SE2)}$$

$$\Delta\varepsilon_{calc}(\lambda) = \sum_i g_i \cdot \Delta\varepsilon_{i,calc}(\lambda) \qquad \text{(SE3)}$$

The scaled theoretical and experimental spectra were obtained using equation SE4:

$$F_{scaled}(\lambda) = F(\lambda) \Big/ \left| F(\lambda_{peak}) \right| \qquad \text{(SE4)},$$

where *F* is absorbance or Δε and the denominator $|F(\lambda_{peak)|}$ is a modulo of the peak value for the chosen characteristic band in corresponding spectrum.

The UV shifts Δλ were obtained while improving the coincidence between calculated and experimental UV spectrum. This UV shift was then taken for simulation of theoretical ECD spectrum.

S71-83. Conformational analysis.

Many Large-Amplitude Motions (LAM) may proceed in compound **1**:

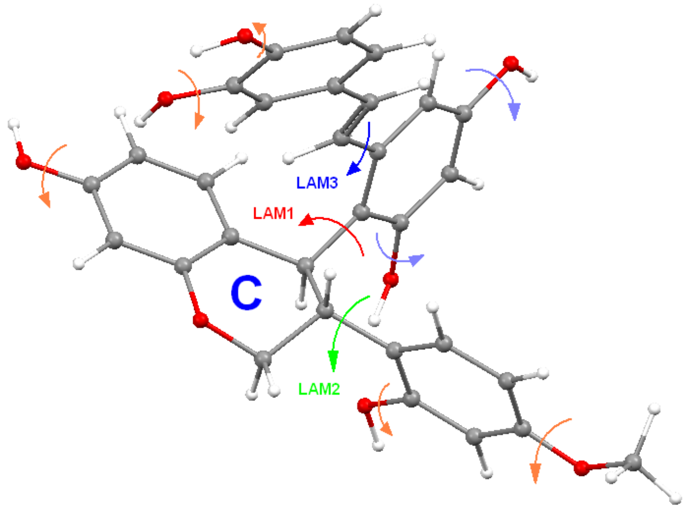


Figure S71. The main LAMs of **1**.

All of the LAMs influenced the UV and ECD spectra of **1**, especially LAM1, LAM2 and LAM3 as well as the inversion of ring C:

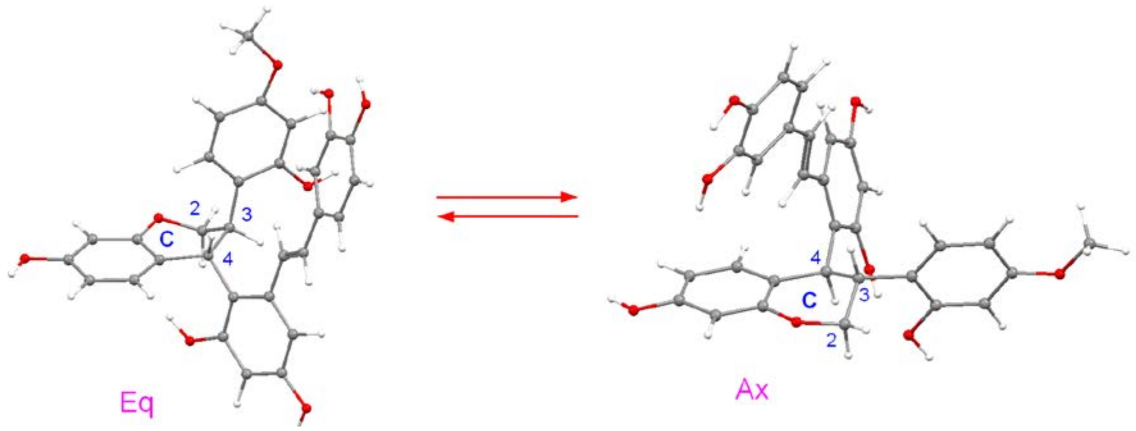


Figure S72. Inversion of ring C.

We used notation “Ax” and “Eq” to distinguish two conformations:

“AX” = axial orientation of H-3;

“Eq” = equatorial orientation of H-3

The inversion of ring C proceeds with overcoming potential barrier $\Delta V^{\#} \geq$ 4.1 kcal/mol. We obtained this value after performing calculations of the potential energy surface (PES) scans along the intrinsic reaction coordinate (IRC) trajectories for several model compounds using B3LYP/6-311G(d) method. First, the transition states (TS) for Ax ↔ Eq rearrangements were localized and proved by calculations of vibrational spectra (all of them contained one imaginary frequency). After that, the IRC-trajectories were calculated, starting from the TS structure and descending to minima on the potential energy surface. The one-dimensional potential V(s), calculated for these model compounds, is shown in Figure S73:

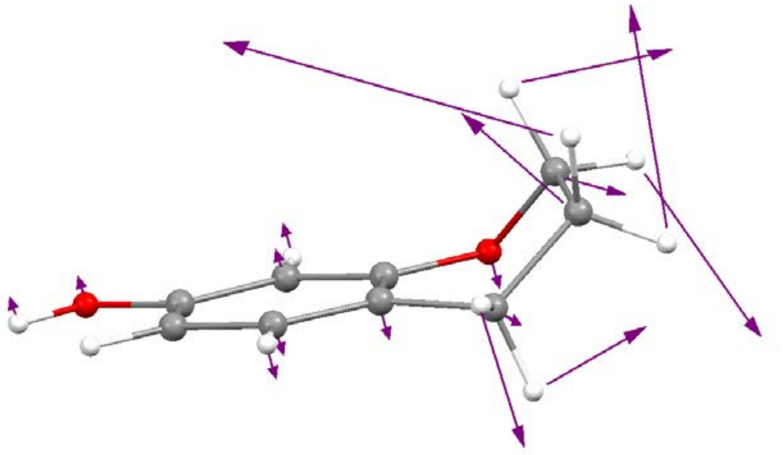

Figure S73. The geometry of the transition state for inversion of pyran ring in chroman-7-ol. The arrows schematically show relative amplitudes of displacements of atoms (for the gradient vector).

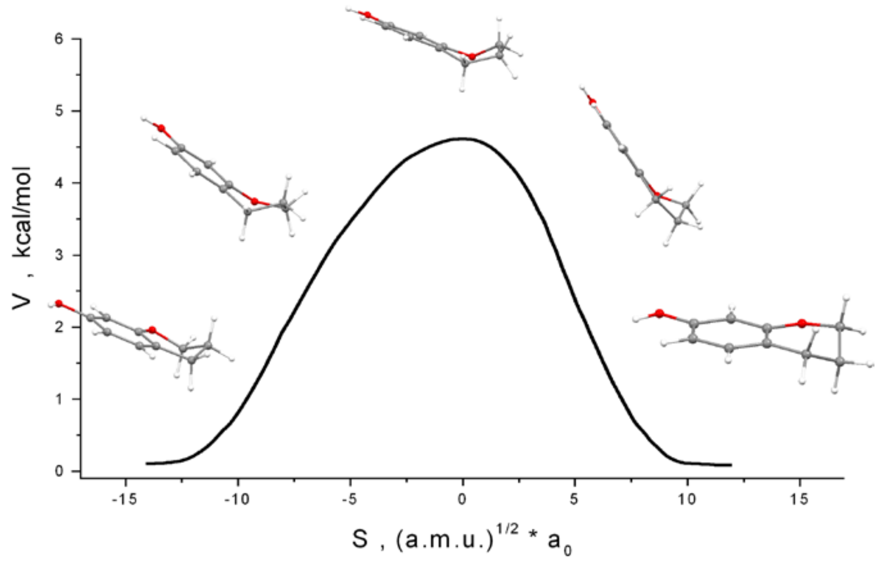


Figure S74. The inversion of chroman-7-ol: the potential energy scan along IRC trajectories.

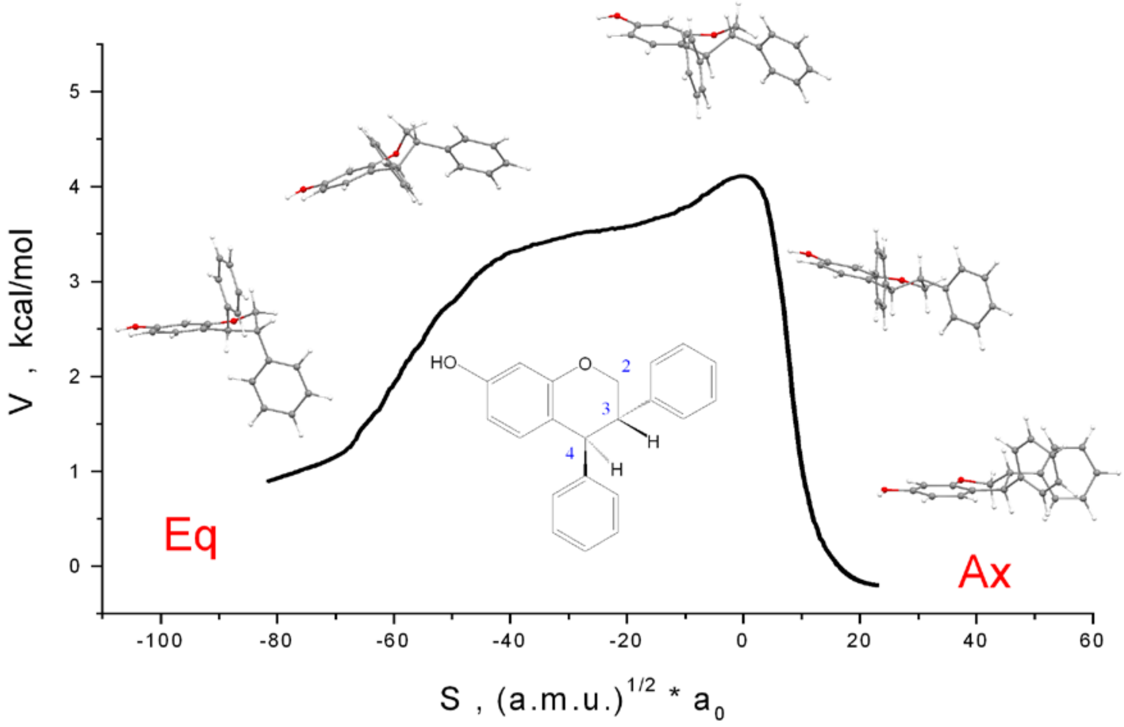


Figure S75. The inversion of (3*S*,4*S*)-3,4-diphenylchroman-7-ol: the potential energy scan along IRC trajectories.

One can see, that in the case of (3*S*,4*S*)-3,4-diphenylchroman-7-ol the potential V(s) is very asymmetric and that energies for “Ax” and “Eq” stable conformations differ for about ~ 1 kcal/mol. “Ax” conformation is more stable.

The rotation of 4-vinylbenzene-1,2-diol fragment around C6″–C7″ bond is hindered. There are two (one in the cases, when substituent at C-4 has orientation, “opposite” to substituent at C-3, denoted as “**1a**” conformation, see text) its “stable” orientations (Figure S76):

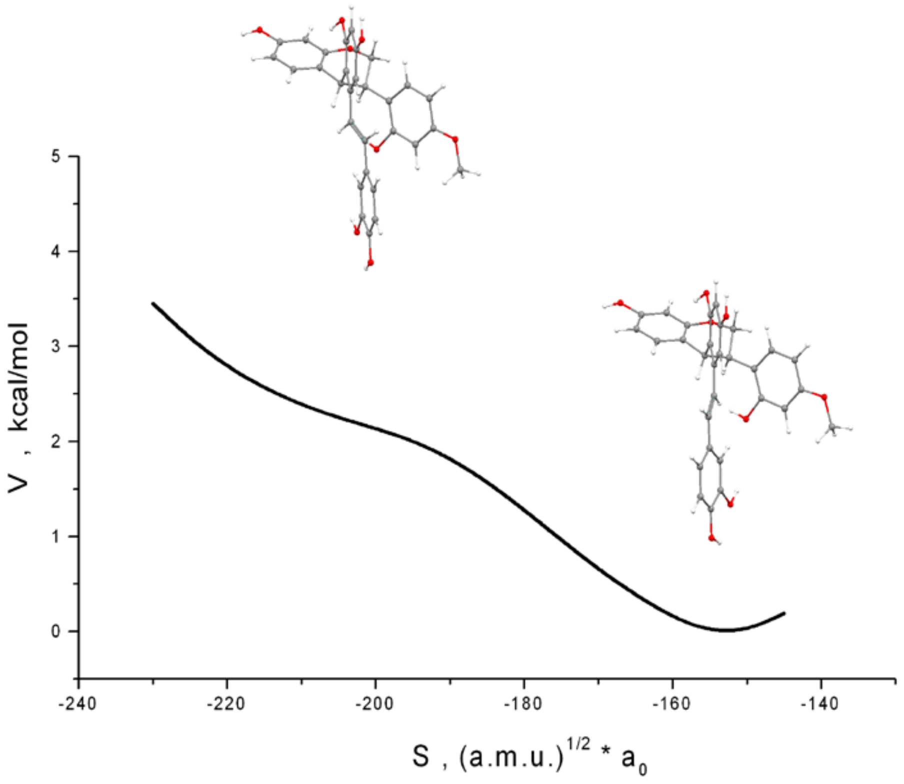


Figure S76. The potential energy as a function of internal rotation of 4-vinylbenzene-1,2-diol fragment around C6″–C7″ bond.

The internal rotation of piceatannol substituent at C4 in diapason -180° ≤ θ4 ≤ +180° may proceed only for “Eq” conformation of cycle C. And even in this case the transfer requires the concerted pronounced transformations of several fragments of molecule:

1) the simultaneous internal rotations of 4-vinylbenzene-1,2-diol fragment around C6″–C7″ bond and of the whole piceatannol substituent at C-4;
2) the internal rotation of 3,5-dihydroxybenzene substituent at C-3.

The potential energy scan along $\theta_4$ is shown In Figure S77:

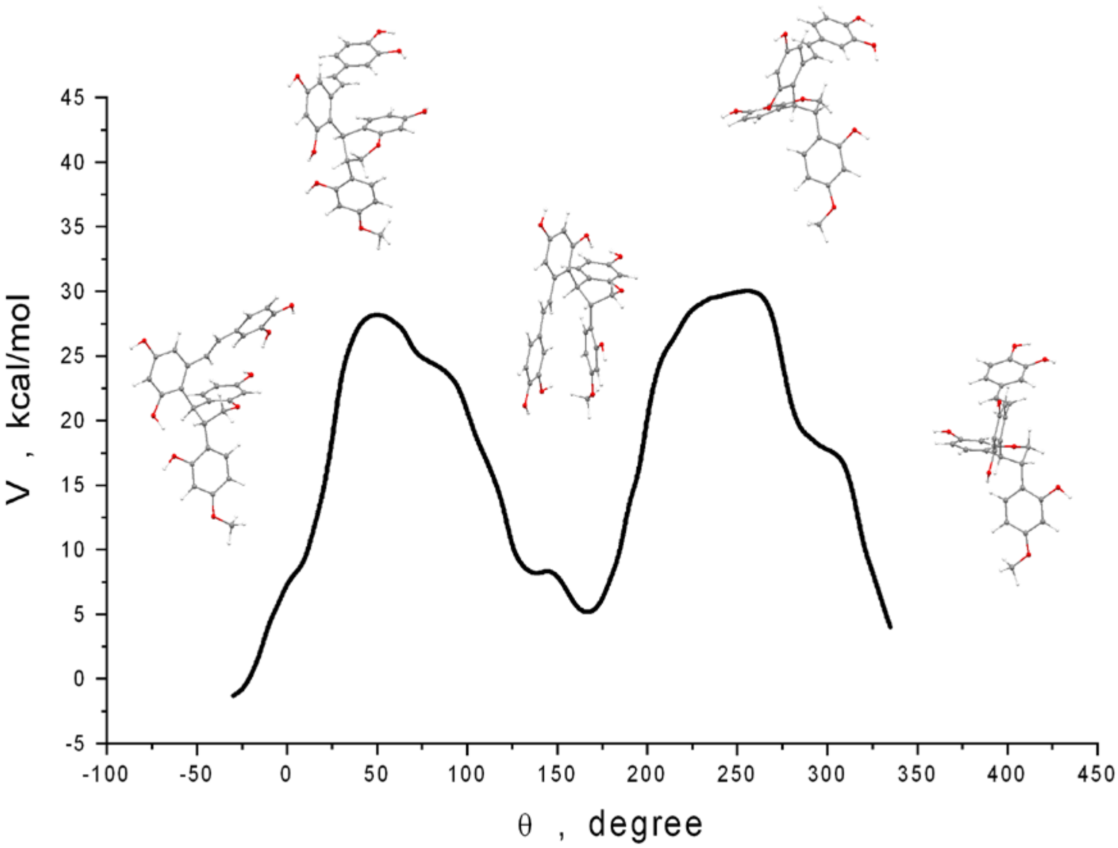


Figure S77. The variation of potential energy during the internal rotation of piceatannol substituent at C-4 around C4–C1″ bond.

We performed the search of the transition state structure (TS) aiming to obtain the lowest possible value for the height of potential energy barrier $\Delta V^{\#}$ ( Figure S78):

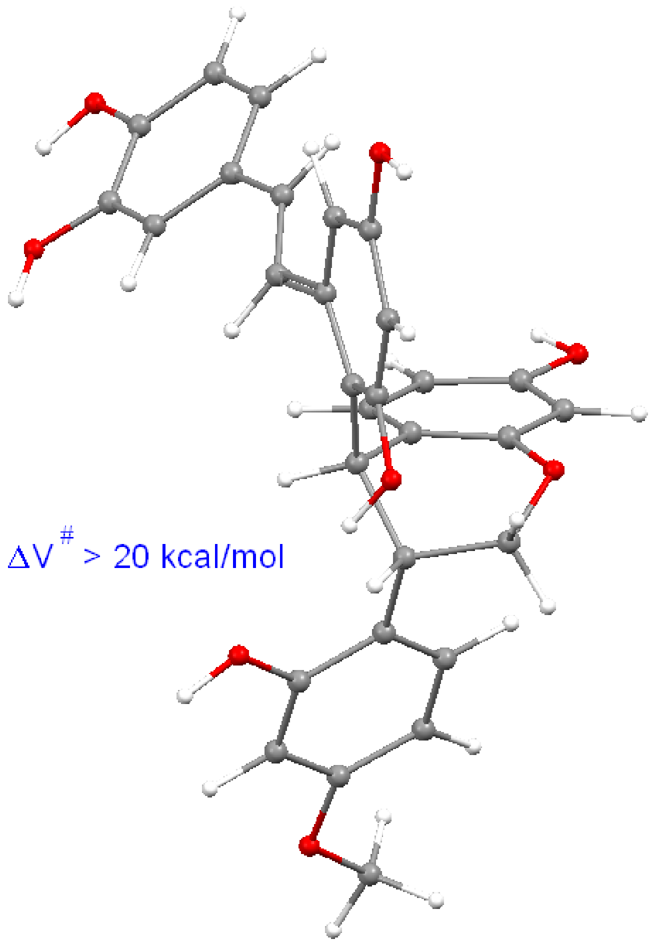


Figure S78. The TS for 3*R*,4*S*-**1a** ↔ 3*R*,4*S*-**1b** rearrangement.

Our estimation gives $\Delta V^{\#} \geq 20$ kcal/mol.

Figure S79. The most stable conformations of 3*S*,4*S*-**1**.

| | |
|---|---|
| 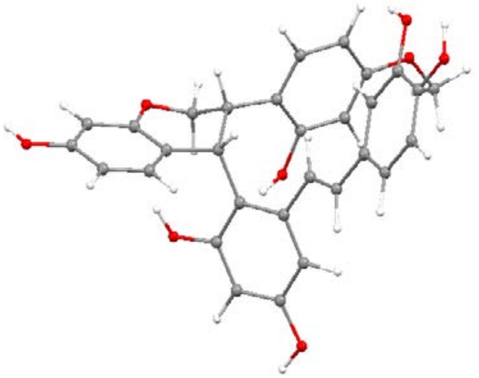 3*S*,4*S*-**1**_1 [0.02] | 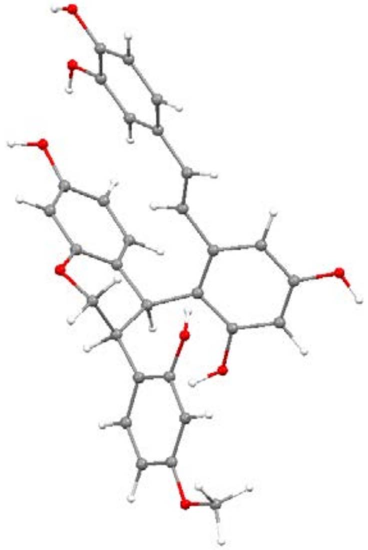 3*S*,4*S*-**1**_2 [<0.01] |
| 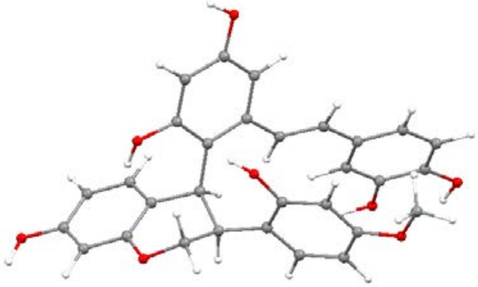 3*S*,4*S*-**1**_3 [0.02] | 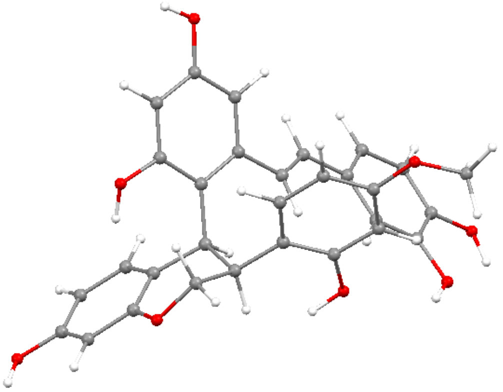 3*S*,4*S*-**1**_4 [0.58] |
| 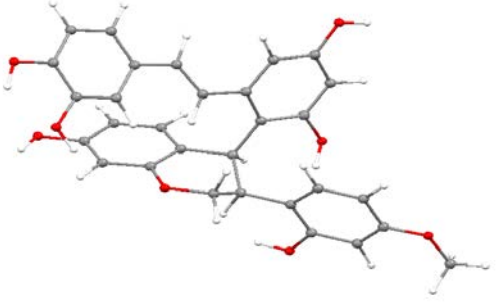 3*S*,4*S*-**1**_5 [<0.01] | 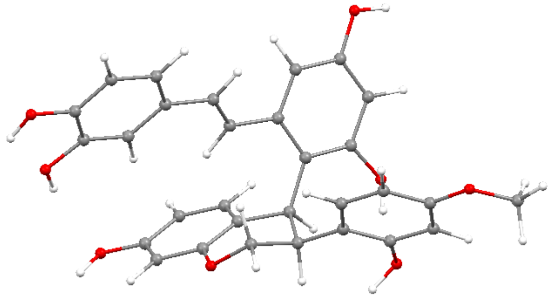 3*S*,4*S*-**1**_6 [0.01] |

| | |
|---|---|
| 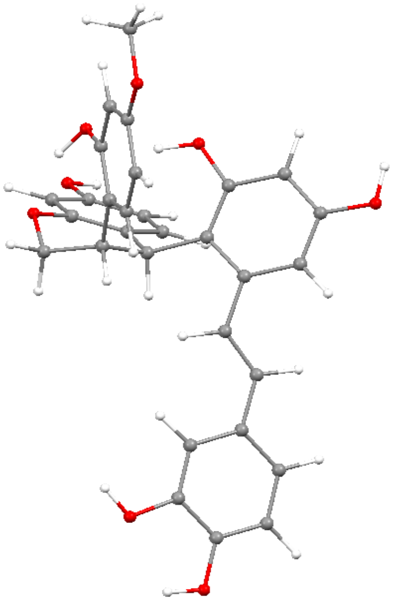<br>3*S*,4*S*-**1**_7 [0.18] | 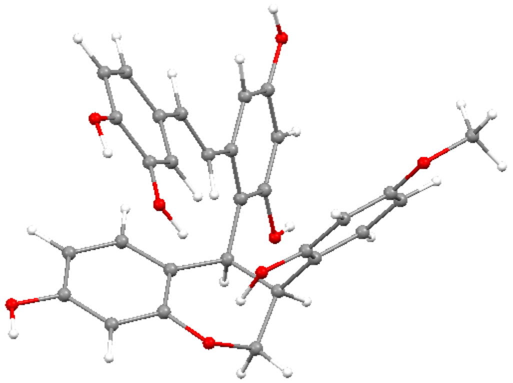<br>3*S*,4*S*-**1**_8 [0.08] |
| 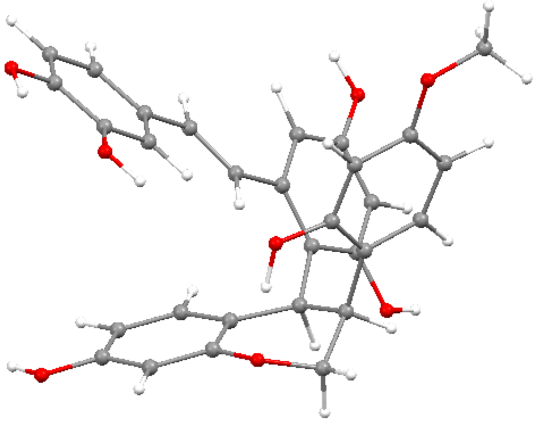<br>3*S*,4*S*-**1**_9 [0.11] | 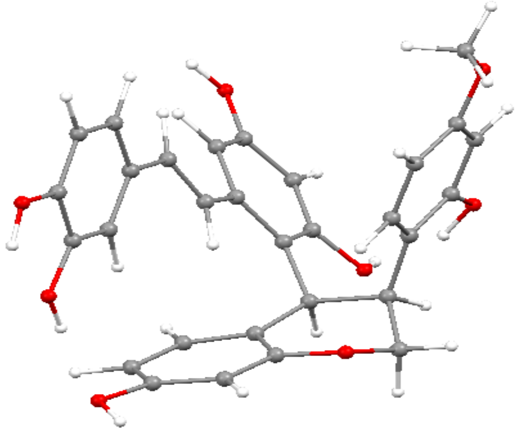<br>3*S*,4*S*-**1**_10 [< 0.01] |

Figure S80 The most stable conformations of 3*R*,4*S* stereoisomer of **1**.

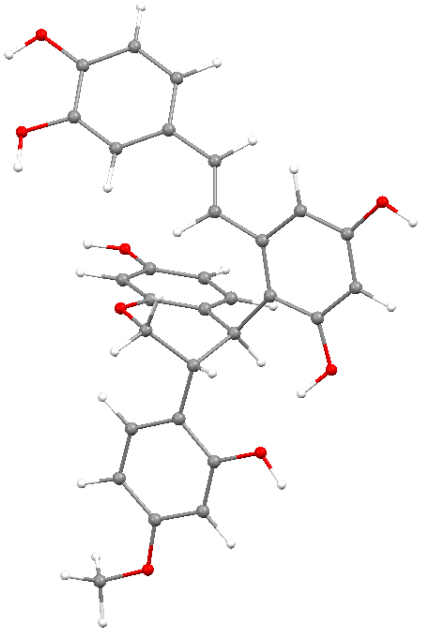

3*R*,4*S*-**1a_**Eq1 [< 0.001]

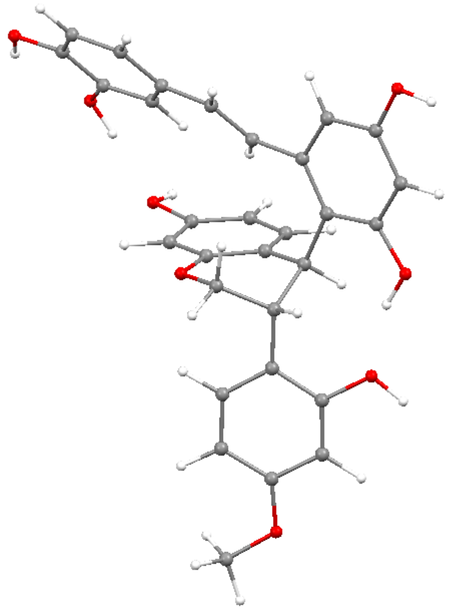

3*R*,4*S*-**1a_** Eq2 [0.003]

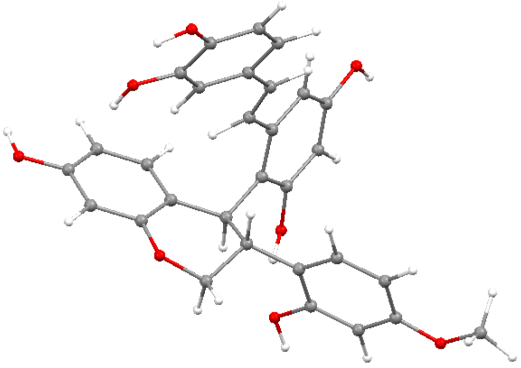

3*R*,4*S*-**1a_** Ax1 [0.446]

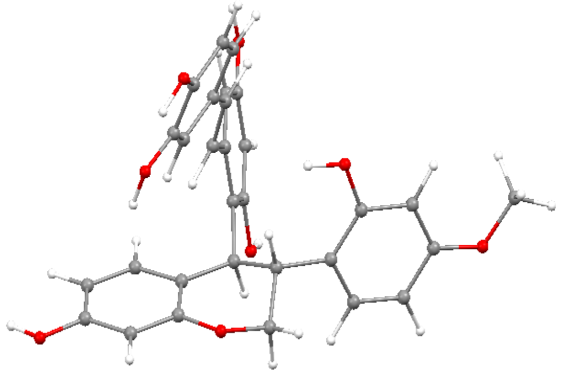

3*R*,4*S*-**1a_** Ax2 [0.426]

| | |
|---|---|
| 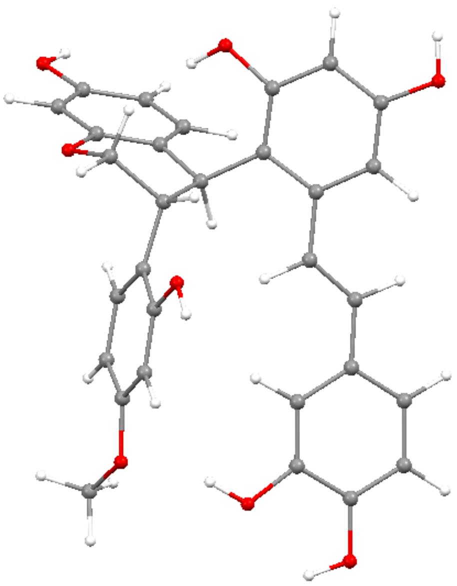 3*R*,4*S*-**1b_**Eq1 [0.007] | 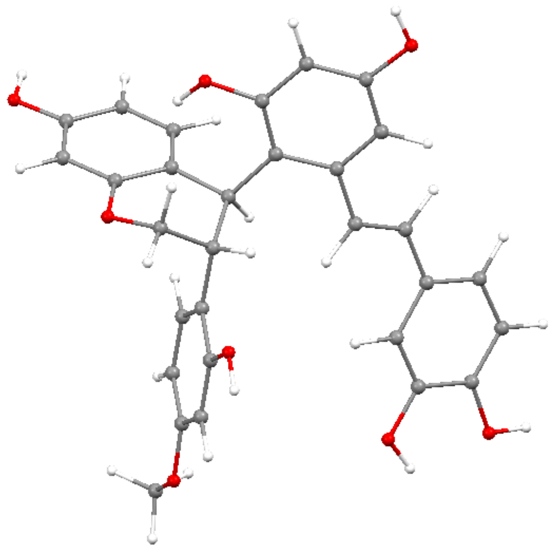 3*R*,4*S*-**1b_**Eq2 [0.045] |
| 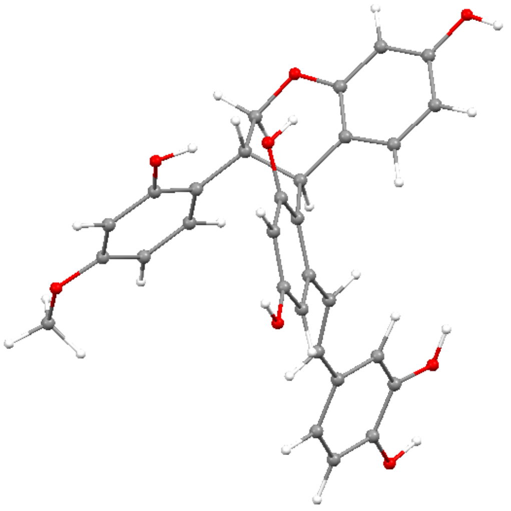 3*R*,4*S*-**1b_** Ax1 [0.007] | 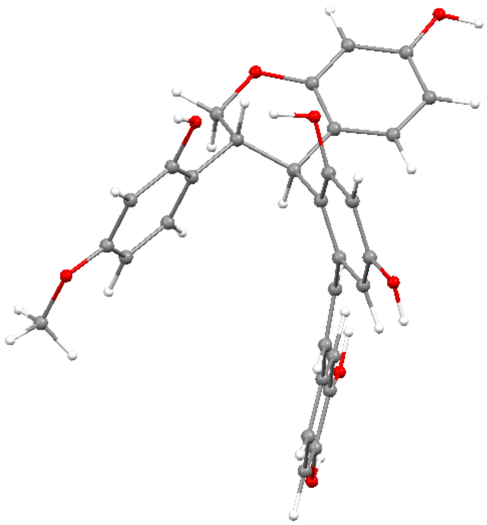 3*R*,4*S*-**1b_** Ax2 [ 0.018] |

*) the statistical weights are calculated in consideration, that 3*R*,4*S*-**1a** and 3*R*,4*S*-**1b** conformations form the mono compound 3R,4S-**1**. In the paper these statistical weights were recalculated, according to consideration, that in the NMR time scale 3*R*,4*S*-**1a** and 3*R*,4*S*-**1b** may be treated as different compounds.

Figure S81 The most stable conformations of 3*S*,4*R* stereoisomer of compound **6**.

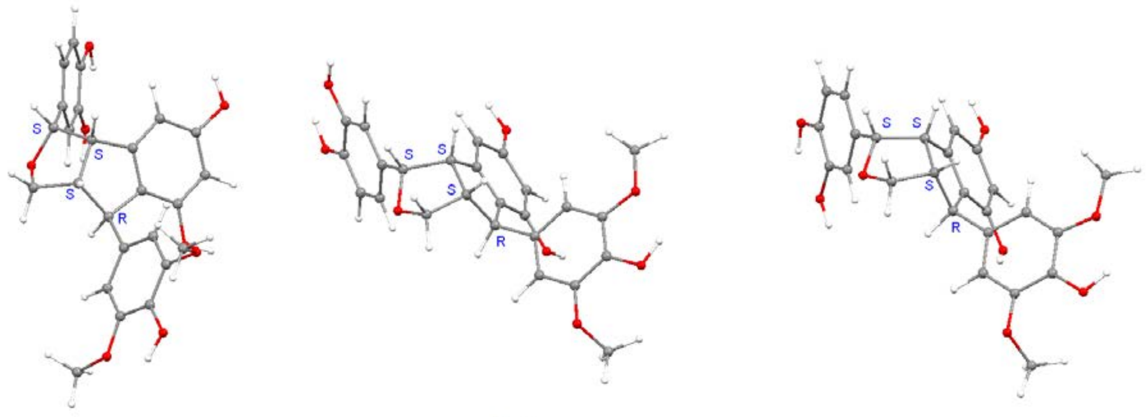


**6-1** [ 0.08 ]

**6-2** [ 0.06 ]

**6-3** [ 0.86 ]

Figure S82, The most stable conformations of 3*S*,4*R*-**4**.

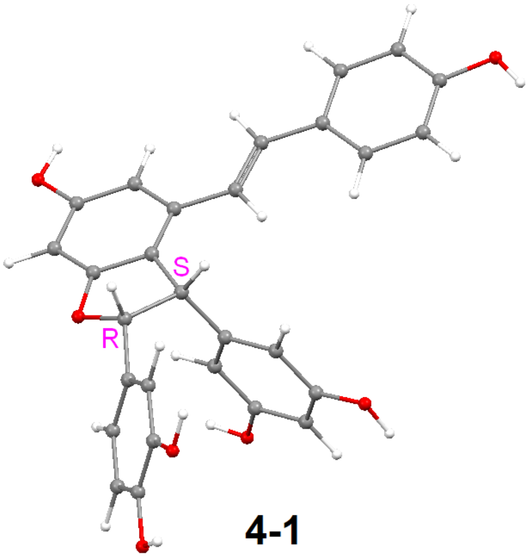


**4-1**
[ 0.39 ]

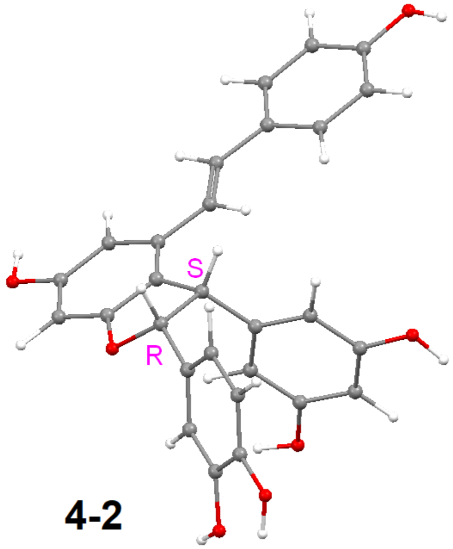


**4-2**
[ 0.61 ]

Figure S83. The most stable conformations of 3*S*,4*R*-**3**.

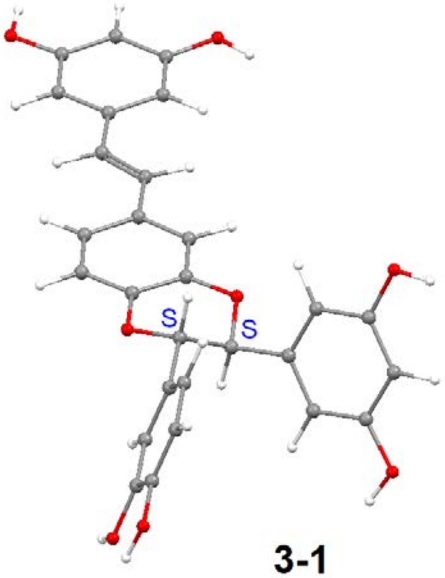


**3-1**
[ 0.31 ]

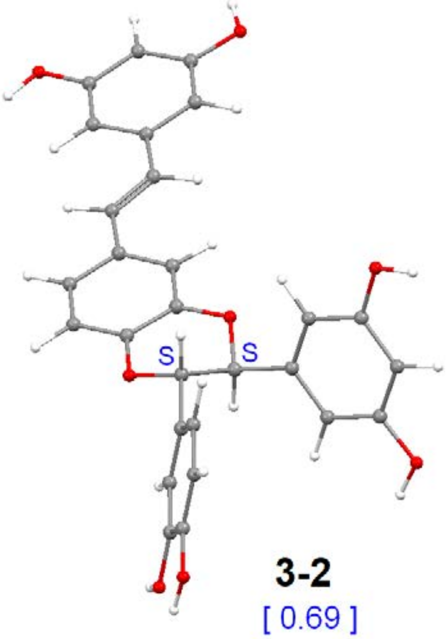


**3-2**
[ 0.69 ]

S84. The modeling of ECD spectra.

The statistically averaged ECD spectra of 3*R*,4*S*-**1** were obtained for two limit cases:

1) $\Delta\varepsilon_{Ax+Eq}$ (3*R*,4*S*-**1**) = $\Delta\varepsilon_{Ax+Eq}$(3*R*,4*S*-**1a_Ax** + 3*R*,4*S*-**1a_EQ** + 3*R*,4*S*-**1b_Ax** + 3*R*,4*S*-**1b_EQ**) - (red plot);

2) $\Delta\varepsilon_{Ax}$ (3*R*,4*S*-**1**) = +$\Delta\varepsilon_{Ax}$(3*R*,4*S*-**1a_Ax** + 3*R*,4*S*-**1a_EQ** + 3*R*,4*S*-**1b_Ax** ) - (blue plot).

Thus, in all cases we take into account both conformations for 3*R*,4*S*-**1a**, whereas Δε(3*R*,4*S*-**1b**) was calculated in two different manner.

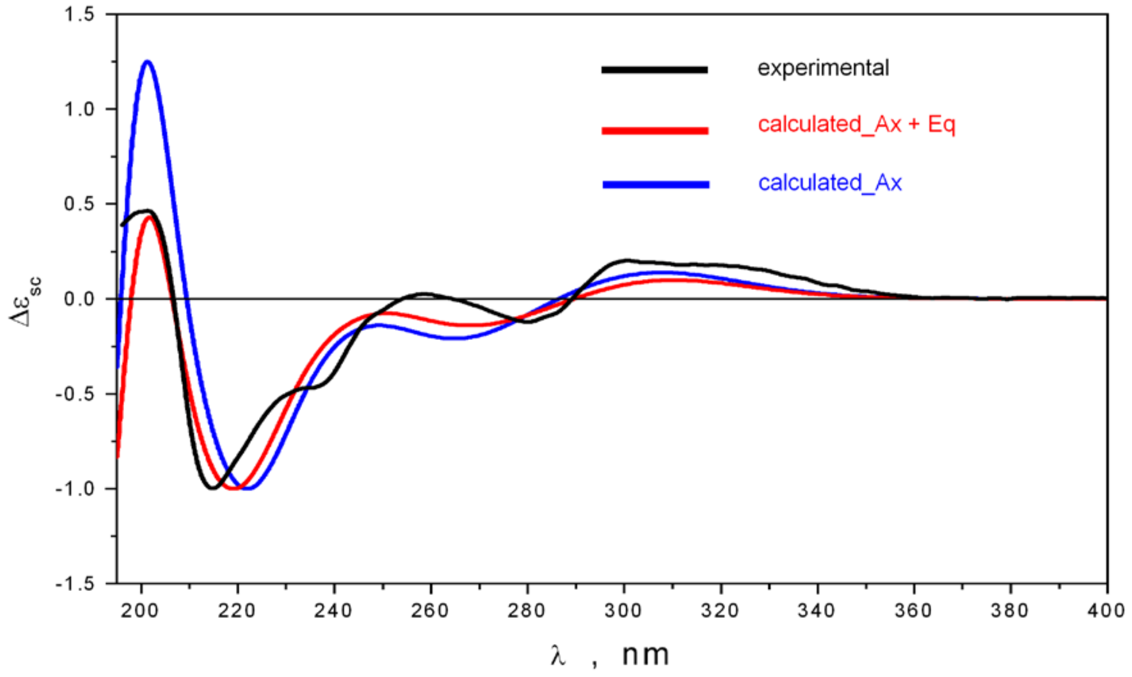


Figure S84. Theoretical ECD spectra of 3*R*,4*S*-**1**, compared with experimental ECD spectrum of **1**.

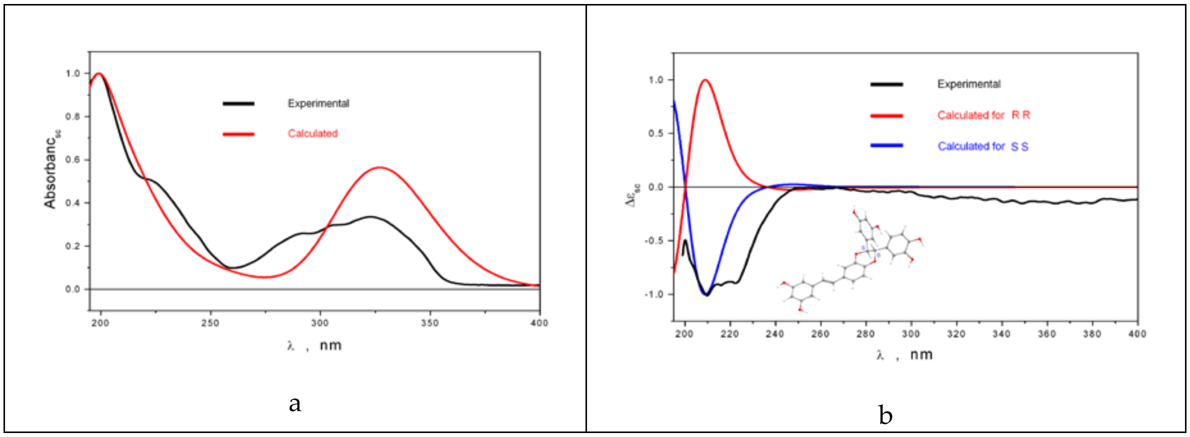


Figure S85. The comparison of experimental and theoretical UV (a) and ECD (b) spectra of **3**.

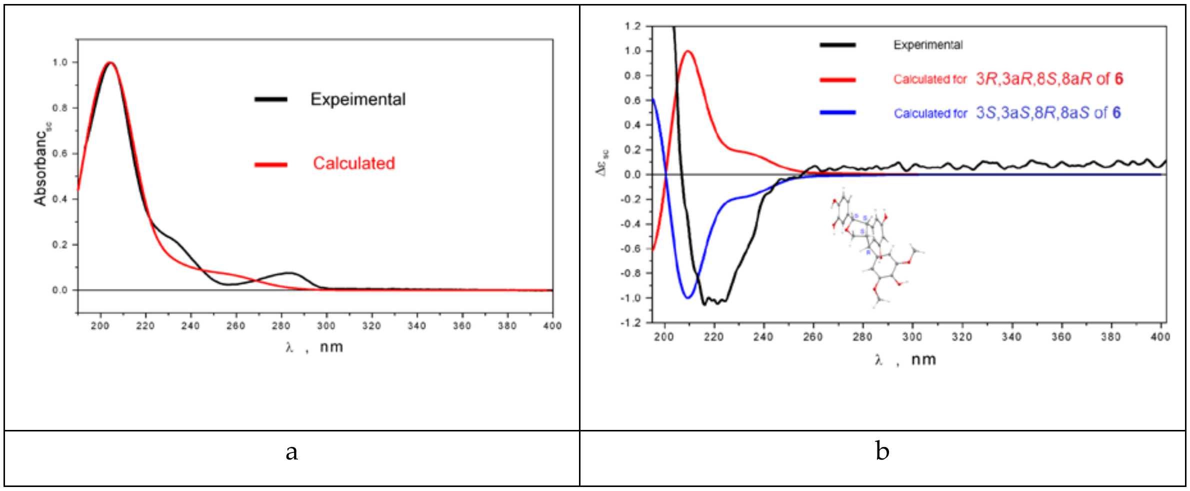


Figure S86. The comparison of experimental and theoretical UV (a) and ECD (b) spectra of **6**.

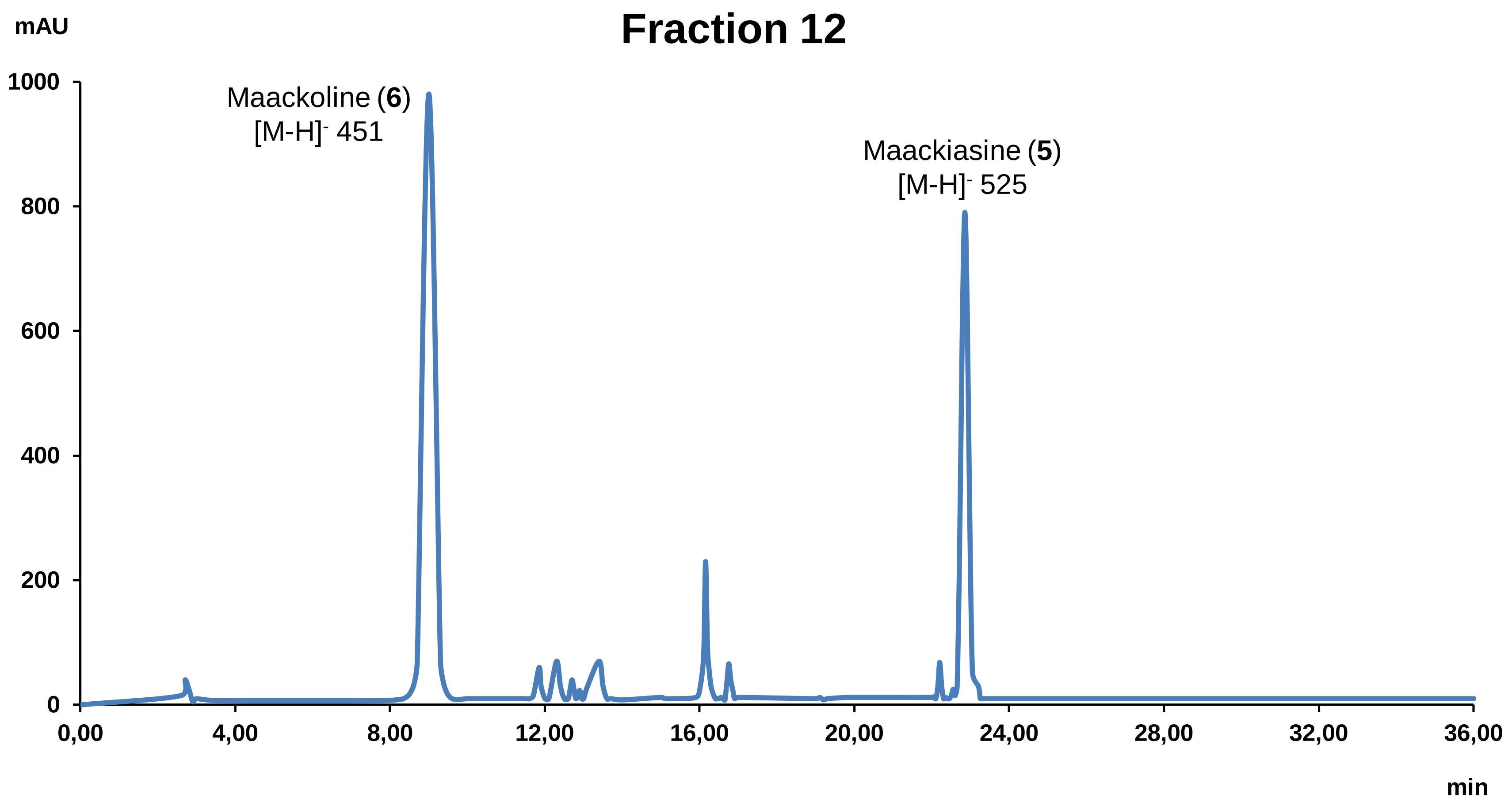


Figure S87. HPLC profile of fraction 12 obtained from *Maackia amurensis* heartwood extract after chromatography on a polyamide column.

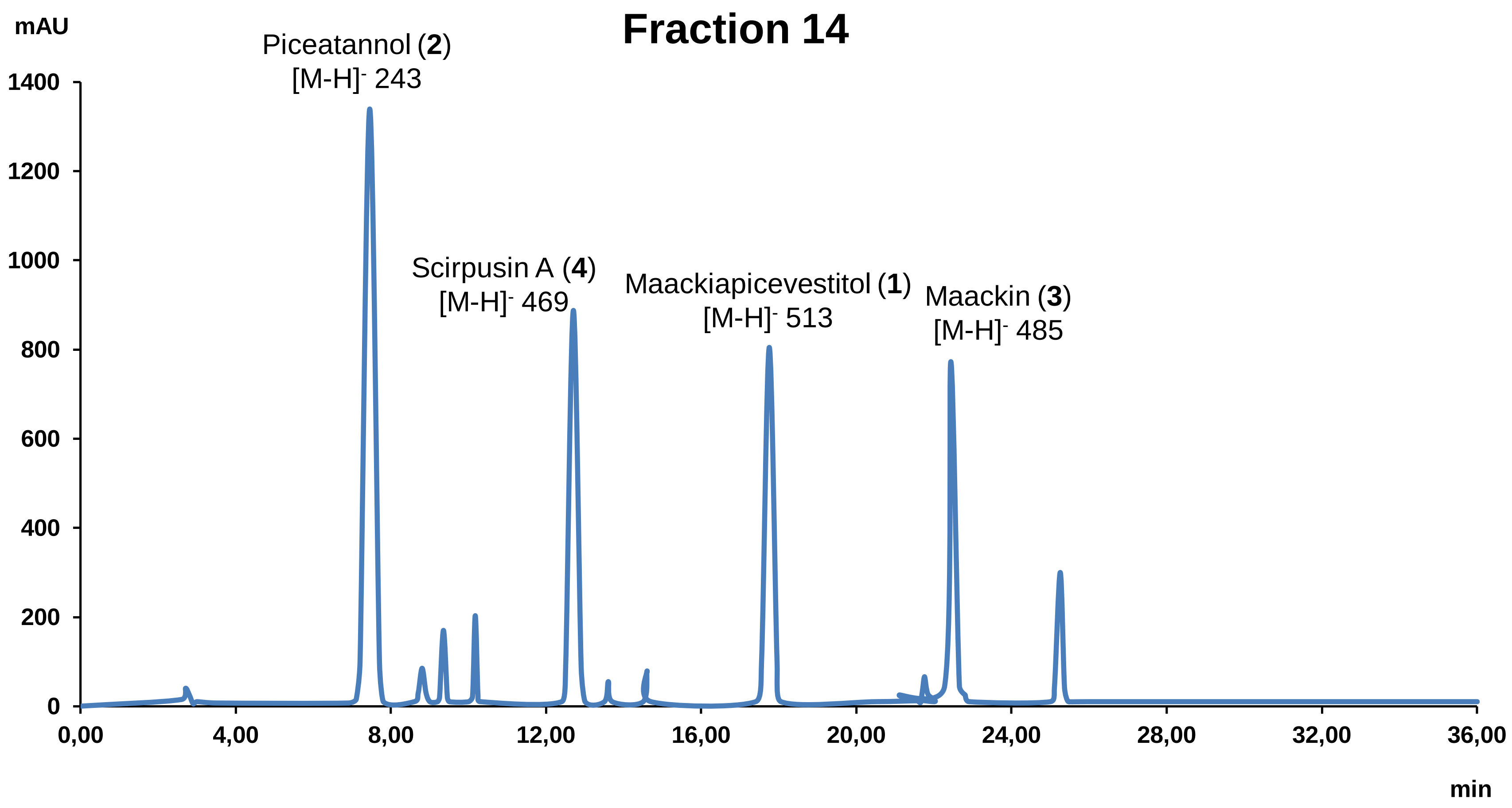


Figure S88. HPLC profile of fraction 1 obtained from *Maackia amurensis* heartwood extract after chromatography on a polyamide column.
